# Engineering Breast Cancer Microenvironments and 3D Bioprinting

**DOI:** 10.3389/fbioe.2018.00066

**Published:** 2018-05-24

**Authors:** Jorge A. Belgodere, Connor T. King, Jacob B. Bursavich, Matthew E. Burow, Elizabeth C. Martin, Jangwook P. Jung

**Affiliations:** ^1^Department of Biological and Agricultural Engineering, Louisiana State University, Baton Rouge, LA, United States; ^2^Department of Medicine, Section Hematology/Oncology, Tulane University, New Orleans, LA, United States

**Keywords:** extracellular matrix, tumor models, cancer microenvironments, 3D bioprinting, cell-ECM interactions, biophysical properties

## Abstract

The extracellular matrix (ECM) is a critical cue to direct tumorigenesis and metastasis. Although two-dimensional (2D) culture models have been widely employed to understand breast cancer microenvironments over the past several decades, the 2D models still exhibit limited success. Overwhelming evidence supports that three dimensional (3D), physiologically relevant culture models are required to better understand cancer progression and develop more effective treatment. Such platforms should include cancer-specific architectures, relevant physicochemical signals, stromal–cancer cell interactions, immune components, vascular components, and cell-ECM interactions found in patient tumors. This review briefly summarizes how cancer microenvironments (stromal component, cell-ECM interactions, and molecular modulators) are defined and what emerging technologies (perfusable scaffold, tumor stiffness, supporting cells within tumors and complex patterning) can be utilized to better mimic native-like breast cancer microenvironments. Furthermore, this review emphasizes biophysical properties that differ between primary tumor ECM and tissue sites of metastatic lesions with a focus on matrix modulation of cancer stem cells, providing a rationale for investigation of underexplored ECM proteins that could alter patient prognosis. To engineer breast cancer microenvironments, we categorized technologies into two groups: (1) biochemical factors modulating breast cancer cell-ECM interactions and (2) 3D bioprinting methods and its applications to model breast cancer microenvironments. Biochemical factors include matrix-associated proteins, soluble factors, ECMs, and synthetic biomaterials. For the application of 3D bioprinting, we discuss the transition of 2D patterning to 3D scaffolding with various bioprinting technologies to implement biophysical cues to model breast cancer microenvironments.

## Introduction

**Figure 1:**
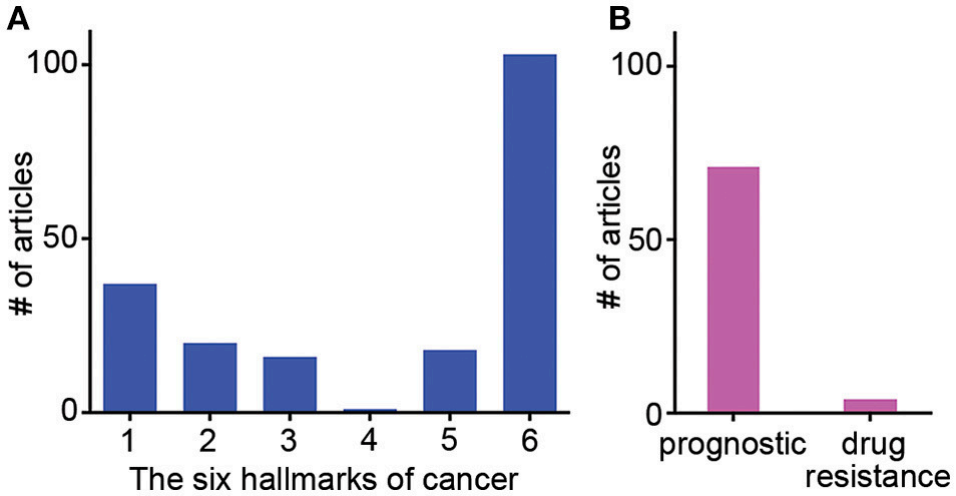
Frequency of studies published showing ECM gene influences on specific hallmarks of cancer, patient prognostics and drug resistance. Results of meta-review survey depicting **(A)** the frequency of publications showing individual ECM-related genes influencing specific hallmarks of cancer and **(B)** the frequency of publications reporting ECM-related genes as a tool for patient prognostic determinations and specific drug resistance.

**Figure 2:**
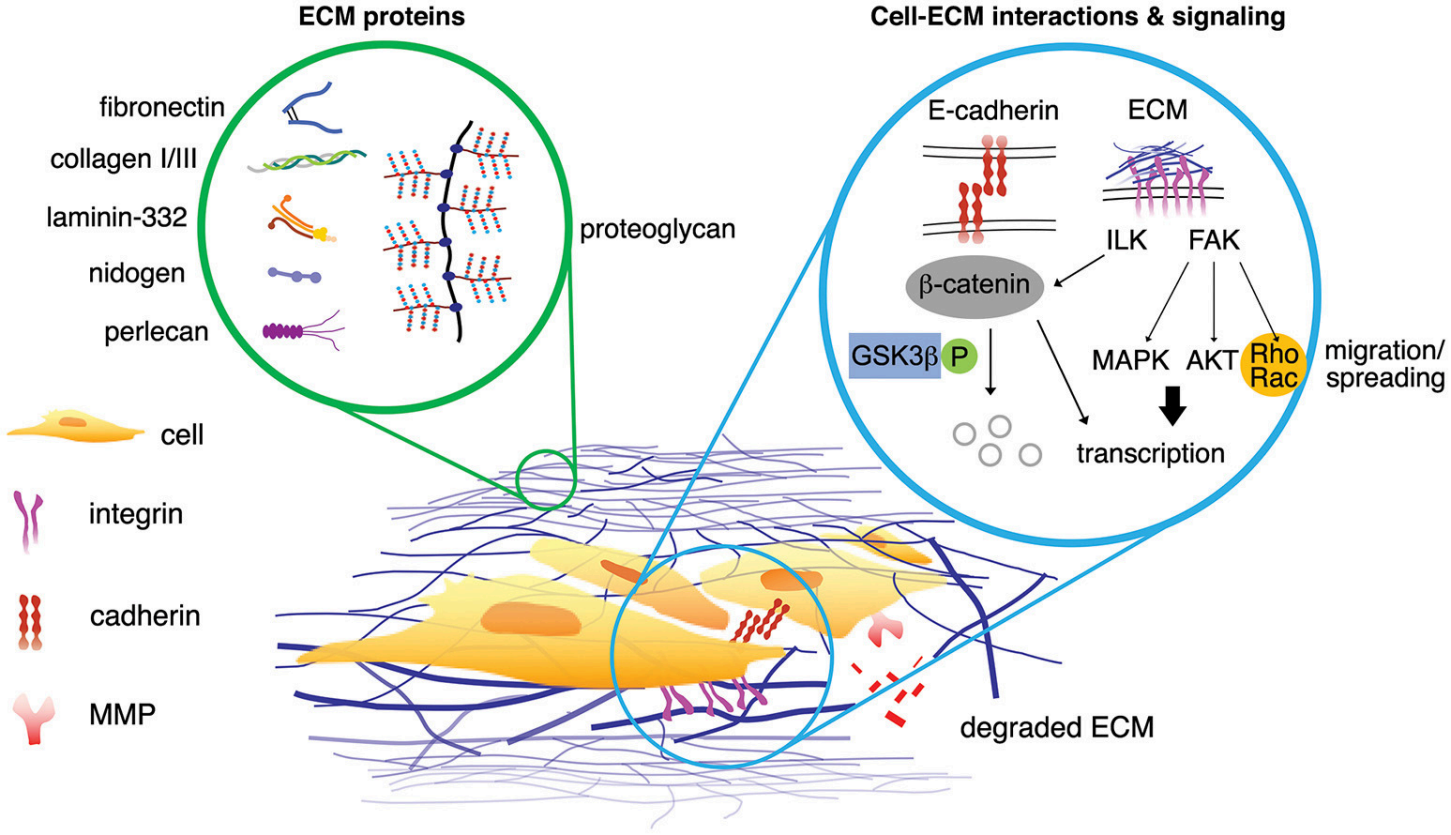
Interstitial matrix proteins and the basement membrane proteins are associated in the breast cancer microenvironments (left circle). The signaling from the ECM proteins propagates via multiple signaling pathways either simultaneously or independently (right circle). Possible scenarios of cell-ECM interactions can initiate signaling of breast cancer cells. Upon phosphorylation, β-catenin dissociates from E-cadherin within adherens junction and is degraded by proteasome. ECM proteins can phosphorylate ILK, which in turn inhibits phosphorylation of GSK3β and activate β-catenin (non-phosphorylated). ECM protein initiates phosphorylation of FAK, leading to enhanced translation of pro-survival and pro-proliferation genes associated with MAPK and AKT pathways. FAK also promotes Rho/Rac activity for actin cytoskeleton assembly, mediating cell migration and spreading. MMPs degrade ECM proteins, generating matrikine.

The breast cancer microenvironment is a combination of cells within the tumor and its stroma, extracellular matrix (ECM), and surrounding signaling molecules. A tumor's stroma is defined as the supportive tissue and associated blood vessels composed of tissue-derived stem cells, adipose tissue, endothelial cells, and fibroblasts. The role for the stroma in cancer is undisputed and the stroma is demonstrated to have tumor-promoting qualities for 5 of the 6 intracellular hallmarks of cancer (Figure 1A and Table 1). Cancer ECM consists of fibrous proteins, basement membrane proteins, proteoglycans, and polysaccharides. These matrix components crosstalk to regulate migration, invasion, proliferation, survival, and epithelial-mesenchymal transition (EMT) signaling cascades (Figure 2). ECM is broadly divided into two groups: the interstitial matrix, formed of fibrillar collagens, and the basement membrane, composed of non-fibrillar proteins and proteoglycans (Mouw et al., 2014). Collagens are the most abundant fibrous proteins within interstitial ECM, providing tensile strength, regulating cell adhesion, supporting chemotaxis, facilitating migration, and directing tissue development (Provenzano et al., 2008; Rozario and DeSimone, 2010; Conklin et al., 2011). The basement membrane is grouped into the basal lamina (collagens IV and VII, laminins, and heparan-sulfate proteoglycans) and the fibrillar reticular lamina (collagen I and III, fibronectin, and elastin). The basal lamina segregates tissue layers, provides a surface for cell adhesion, catalyzes cellular communication, and protects tissues from biochemical and biophysical insult. Proteoglycans are notable for their negative charge allowing growth factors (GFs) and cell surface receptors to sequester or tether GFs, establishing cell adhesion via the binding of essential cations and the movement of various molecules throughout the ECM.

**Table 1:** ECM-associated genes and proteins reported to influence specific hallmarks of cancer^a^.

	**1. Self-sufficiency in growth signals**	**2. Insensitivity to anti-growth signals**	**3. Evading programmed cell death**	**4. Limitless replicative potential**	**5. Sustained angiogenesis**	**6. Tissue invasion and metastasis**
Genes	ADAMTS1, COL/LOX, COL1, COL1A2, COL6A1, COL6A2, COL8A1, CTNNB1, ICAM1, ITGA6, ITGB1, LAMA1, LAMA2, LAMA3, LAMC2, LAMA67R, MMP10, MMP11, MMP12, MMP13, MMP14, MMP15, MMP16, MMP8, SELP, SPARC, TIMP2, TIMP3, VCAM1, VCAN	ADAMTS1, COL6A1, ICAM1, ITGB1, LAMA3, MMP10, MP11, MMP12, MP13, MMP8, SELP, TIMP2, TIMP3, VCAM1, VCAN	ADAMTS8, COL4A2, COL6A1, CTNNA1, CTNNB1, LAMA67R, LAMA1, LAMC1, MMP1, MMP10, MMP15, TIMP3, VCAM1	COL6A1	ADAMTS1, ADAMTS13, ADAMTS8, COL, COL15A1, HS, LAMB1, MMP1, PECAM1, SPARC, TIMP2, TIMP3, VCAN	ADAMTS1, ADAMTS8, ANTXR1, COL, COL1, COL12, COL12A1, COL16A1, COL1A2, COL4, COL4A2, COL4A5, COL6A1, COL6A2, COL8A1, CTHRC1, CTNNB1, ELN, FN, HAS1, HDAC, ICAM1, ITGA6, ITGB4, ITGB-α6β1, LAMA3, LAMC1, LAMC2, LAMA332, LAMA67LR, MMP1, MMP10, MMP11, MMP12, MMP13, MMP14, MMP15, MMP16, MMP2, MMP3, MMP7, MMP8, MMP9, SPARC, TIMP1, TIMP2, TIMP3, VCAM1, VCAN
Associated with Specific Cancers	BLADDER, BREAST, CERVICAL, COLON, ESOPHAGEAL, GASTRIC, GENERIC, HEAD AND NECK, LIVER, LUNG, OVARIAN, PROSTATE, UTERUS, ENDOMETRIAL	ENDOMETRIAL, BLADDER, BREAST, CERVICAL, COLON, ESOPHAGEAL, HEAD AND NECK, LIVER, LUNG, OVARIAN, UTERUS	BILE DUCT CARCINOMA, BREAST, CERVICAL, COLON, ESOPHAGEAL, LIVER, LUNG, OVARIAN, PANCREATIC, PROSTATE	PROSTATE	BRAIN, BREAST, COLON, GASTRIC, LIVER, LUNG, OVARIAN	BLADDER, BONE, BRAIN, BREAST, CERVICAL, COLON, ENDOMETRIAL, ESOPHAGEAL, GASTRIC, HEAD AND NECK, KIDNEY, LIVER, LUNG, ORAL, OVARIAN, PANCREATIC DUCTAL ADENOCARCINOMA, PROSTATE, SKIN, UTERUS
References	37	20	16	1	18	103

a *Methods for meta-review survey: The articles reported in Tables 1–4 were using Google Scholar and PubMed.; the searches were restricted to keywords within the title of the publications as well as to publications dated from 2010 to 2017. Table 4 shows the ECM-associated genes that were used as primary keywords for this table, specific cancers types (breast, liver, ovarian, and brain) were used as secondary keywords, an expansion of cancer types (prostate, head and neck, colon, oral) were used as tertiary keywords, and the generic keywords “tumor” and “cancer” were used as quaternary keywords. Search parameters began with a combination of individual primary keywords with individual secondary keywords. In the instance that combinations of individual primary and secondary keywords did not generate articles associating ECM-gene influence on the various hallmarks of cancer, the search was then expanded using individual primary and tertiary keywords. In the instance that this expansion did not assist in producing relevant articles, the search was further expanded using individual primary and quaternary keywords. On the rare occasion that no relevant articles were found using this search protocol, the restriction on publication date was removed and the search protocol was repeated. Finally, a few articles were also included in Tables 1–3 that either reported the influence of ECM-associated genes that were not listed in Table 4 or used generic descriptions of the ECM-associated genes listed in Table 4. An attempt was made to report a minimum of five articles per each individual ECM-associated gene. At the time that the search was performed, it became apparent that some ECM-associated genes from Table 4 had been investigated more thoroughly in the literature than others, which is reflected in the frequency of reported articles associated with those specific ECM-associated genes in Tables 1–3. Keywords: Primary: Specific ECM-associate genes; Secondary: breast, liver, ovarian, brain cancers; Tertiary: prostate, head and neck cancers, colon, oral cancers; Quaternary: tumor and cancer*.

Cancer cells decrease cellular adhesion to matrix as they become more invasive and metastatic. Supporting cells within the tumor microenvironment secrete signals that advance tumor progression, drug resistance, and enhance cancer cell invasion and metastasis. While the tumor microenvironment is influential on all aspects of cancer progression, current *in vitro* models fail to accurately recapitulate tumor microenvironments, both structurally and molecularly. In addition, the tissue specific differences in matrix composition and GFs that exist between the primary breast and organ systems that are sites of metastatic breast cancer seeding are not mimicked in current tumor models. Thus, it is imperative to develop a 3D culture model that mimics the human tumor matrix with structural and chemical definition while pursuing cancerous tissue specificity. In many cases, ECM protein-based scaffolds with self-assembling capability, such as Matrigel™ or collagen, are used as an accessible, primary means of recapitulating tumor models in 3D culture. However, a few fundamental pitfalls exist within this culture paradigm. The self-assembled ECM proteins only partially match the native and developing tumor ECM. For example, Matrigel™ lacks species specificity, displays batch-to-batch variability in biochemical and biophysical properties (Benton et al., 2014), and cannot be easily tuned for systematic studies (Asghar et al., 2015; Leggett et al., 2017); the mechanical resilience of collagen gel (primarily with collagen type I) is limited in comparison to the native and developing tumor; and cells of one tissue type are used while neglecting intratumor and stromal interactions from other cell types. This leaves the research community with a dearth of accessible, effective 3D culture systems.

As we enhance our understanding of tumor ECM and native-like breast cancer microenvironments, advanced biomaterials and 3D bioprinting (3DBP) are becoming rapidly accessible options to engineer 3D microenvironments. This provides an opportunity to create new, predictable 3D culture platforms that can precisely emulate the breast cancer microenvironment. Ultimately, the ability to design and reengineer the tumor matrix allows us to evaluate the individual contributions of tumor-associated ECM while providing a platform to identify and test novel anti-cancer therapeutic strategies by accurately modeling ECM proteins.

### Breast cancer microenvironments

#### Stromal component

The stromal component of breast tumors contains immune cells, fibroblasts, adipose tissue, endothelial cells, and tissue-derived stem cells. These stromal cells heavily influence how breast cancer progresses by secreting factors, altering phenotype, and reorganizing themselves. Hallmarks during breast cancer progression and cancer-specific interactions of stromal cells and ECMs are summarized in Tables 1–4. For instance, stromal remodeling of the ECM via MMPs (matrix metalloproteinases) and TIMPs (tissue inhibitor of metalloproteinases) is a critical factor to the definition of cancer hallmarks. While many hallmarks are associated with the ECM in the stromal components, “4-Limitless replicative potential” is reported only in reference to COL6A1 (Figure 1A). The 6th hallmark “Tissue invasion and metastasis” has 11% more publications than all five of the other hallmarks combined. This implicates that tissue invasion and metastasis is well appreciated in the stromal component, however the role for ECM in supporting the full spectrum of individual hallmarks should not be overlooked (Figure 1A). However, a larger-scale, meta-review survey is needed to provide substantial support for this specific claim.

Aside from matrix, the cellular milieu found in the tumor microenvironment should also be considered. As a whole, interactions between the immune system and the breast cancer microenvironment remain out of the scope of this review and have been well documented by others (Gajewski et al., 2013; Haabeth et al., 2014; Williams et al., 2016). However, it is of significance that immune cells play important roles by altering the stromal signaling and ECM composition of the tumor microenvironment. For instance, angiogenesis and inflammation are two phenomena which alter the matrix components of breast cancer microenvironments. Macrophages, neutrophils, and CD4^+^ T-helper cells (T_H_2, T_H_1, and T_H_17) are immune cells involved in both of these processes. Macrophages secrete inflammatory factors (TNFα, IL-6, IL-1β, IL-8), pro-angiogenic factors (VEGFA, CXCL12, FGF2), and ECM remodeling factors (MMPs, TGF-β). These factors help promote endothelial cell proliferation and can induce epithelial-to-mesenchymal transition (EMT) in cancer cells (Arnold et al., 2015; Williams et al., 2016; De Palma et al., 2017). Macrophage infiltration at the invasive front of human breast cancer was positively correlated with ECM stiffness (Acerbi et al., 2015). Neutrophils secrete (VEGFA, FGF2, MMP9) and promote inflammation, proliferation, invasion, and angiogenesis (Powell and Huttenlocher, 2016; De Palma et al., 2017). CD4^+^ T-helper cells secrete pro-angiogenic and inflammatory factors that recruit macrophages and neutrophils and modulate their phenotypes. T_H_2 cells secrete IL-4, IL-6, and IL-13; T_H_1 cells secrete IFN-γ; and T_H_17 cells secrete IL-17 (Borthwick et al., 2013; Mora-Solano and Collier, 2014; De Palma et al., 2017). Secreted factors IL-1β, IL-4, IL-13, and TNF-α are direct indications of ECM remodeling in liver and pulmonary fibrosis (Liu T. et al., 2012; Borthwick et al., 2013). In addition, decellularized colorectal tumors polarized macrophages to an anti-inflammatory phenotype (secretions of IL-10, TGF-β, and CCL18). These anti-inflammatory macrophages where then used to stimulate colorectal cancer cell invasion through Matrigel™ invasion assays. The altered macrophages increased cancer cell invasion when compared to macrophages differentiated in normal matrices (Pinto et al., 2017).

Fibroblasts in breast cancer are phenotypically altered to enhance tumorigenesis and termed cancer-associated fibroblasts (CAFs). When fibroblasts are recruited by cancer cells, CAFs exist permanently in the wound healing state while producing more matrix proteins, secreting pro-inflammatory and angiogenic factors (TGF-β, VEGF, IL-6, and SDF-1), and degrading matrix proteins by MMPs. CAFs secrete collagens type I, III, V, and VI to alter estrogen signaling in addition to reducing chemotherapy drug uptake (Mao et al., 2013). CAF co-culture with an estrogen receptor positive (ER^+^) breast cancer cell line reduced tamoxifen-induced apoptosis, indicating that CAFs can alter response to endocrine therapy as well (Martinez-Outschoorn et al., 2011). Within normal breast tissue, fibroblasts and myofibroblasts promote inflammation and angiogenesis. Following breast implant surgery, fibroblasts in patient samples of breast capsular tissue were found to secrete pro-inflammatory and pro-fibrotic signals stimulating differentiation among other fibroblasts and increasing collagen deposition (Segreto et al., 2017). Importantly, CAFs drive angiogenesis of tumors by secreting VEGFA and initiating pro-angiogenic paracrine loops with themselves by secreting PDGFC. The CAF secretome also helps recruit vascular endothelial cells to form new blood vessels (De Palma et al., 2017).

Adipocytes provide breast cancer with cellular energy substrate in the form of triglycerides, engage in various feedback loops with cancer, and secrete multiple tumorigenic, pro-inflammatory cytokines. Specifically, adipocytes are a major source of estrogen signaling for breast cancer cells due to native aromatase activity. Breast cancer cells then secrete factors that stimulate and amplify aromatase activity in adipocytes, initiating a positive feedback loop. Adipocytes secrete adipokines (LEP, ADIPOQ, IL-6, IL−1β, TNFα), MMPs, and PAI-1, to enhance cancer progression. Breast cancer cells show the ability to promote dedifferentiation among adipocytes, while increasing more stem-like cells in the stroma (Bielli et al., 2014; Hoy et al., 2017).

Endothelial cells are often recruited by native tumor immune cells, fibroblasts, and adipocytes to form new blood vessels through a large variety of secreted factors. Additionally, tumor native endothelial cells are often altered in phenotype, gene expression, and secretome. Typically, tumor native blood vessels have multiple fenestrations and loose intercellular junctions resulting in leakage. Interestingly, tumor endothelial cells have upregulated VEGF and EGF receptors. These endothelial cells also secrete TNFα which helps stimulate cancer cell secretion of CXCL1/2 resulting in increases in cancer cell survival (Bussard et al., 2016; De Palma et al., 2017).

Within the tumor, a small subpopulation of cells is identified as cancer stem cells (CSCs). Cancer stem cells are indicated by cell surface markers CD44^+^/CD24^low^ and CD44^+^/CD49f^+^/CD133/2^+^ as well as ALDH1 activity (Atkinson et al., 2013). Similar to adult mesenchymal stem cells (MSCs), CSCs self-renew and when dormant, exhibit resistance to toxic agents including those administered as primary therapy (Jeong et al., 2016). While the precise mechanisms of metastatic recurrence remain to be elucidated, CSCs reinitiate tumor formation following primary therapy. Recently, profiling of gene expression of human tumor metastasis and recurrence demonstrates that ECM-integrin interaction pathways are enhanced in that the ECM can dictate intracellular signaling of CSCs to evoke either a tumor suppressive or oncogenic effect on breast carcinomas, depending on the ECM composition (Wu et al., 2014). The fluid nature of CSCs in addition to their receptivity to the microenvironment poses as a powerful adversary in the treatment of triple negative breast cancer (TNBC). For example, an adjuvant therapy targets both the microenvironment and CSCs (Ye et al., 2014). One limiting and critical factor in this approach is that little is known about the microenvironment that remains following primary therapy. The characterization of the remnant microenvironment, which provides CSCs with external and intrinsic signaling, leads to novel methods for intervention. The remnant microenvironment will be discussed further in section Primary and Secondary Tumor Site Differences.

#### Cell-ECM interactions and activated targetable pathways

Signaling from tumor matrix acts in a tumor suppressive or oncogenic manner depending on its composition. Specifically, the binding of cells to cancer matrix induces AKT (protein kinase B)/MAPK (mitogen-activated protein kinase) proliferative and survival pathways through integrin mediated signaling. For example, β1-integrin binds to fibronectin, collagen, or laminin and undergoes a conformational change, which allows binding of key kinases such as FAK (focal adhesion kinase) and ILK (integrin linked kinase). Then, actin stress fibers and focal adhesion cytoskeletal proteins are assembled at the area of clustered integrins to amplify signals, forming a focal adhesion complex. These complexes of fibers and kinases activate phosphorylation cascades revving up signaling through Rho and MAPK pathways, further affecting proliferation, differentiation, polarity, contractility, and gene expression (Figure 2). Consequently, the matrix directs cell invasion and metastasis as cancer cells are guided by the fibril proteins into distal parts of tissue (Provenzano et al., 2008; Conklin et al., 2011). Furthermore, cell seeding on matrices with multiple discrete stiffness or with stiffness gradients becomes increasingly more prominent in studies evaluating drug resistance (Shin and Mooney, 2016). Clinical evaluation of breast tumor biopsies demonstrates that the stiffness of breast tumor increases both with induction of tumor formation and focal adhesion markers such as FAK (Levental et al., 2009; Schedin and Keely, 2011; Almstedt et al., 2017). While FAK is increased in all breast cancer patient samples, it specifically correlates with the ER^+^ luminal B subtype (Almstedt et al., 2017). Luminal B breast cancer by convention is ER^+^ with altered ER signaling pathways and enhanced proliferation. Characteristically, PGR (progesterone receptor) is repressed in luminal B breast cancer and there is enhanced resistance to endocrine therapies (Creighton, 2012). HER2 (epidermal growth factor 2) amplified tumors are resistant to endocrine therapy and have increased proliferation compared to luminal A subtypes. Increased collagen expression and integrin activation is observed in HER2 derived mouse tumors (Hanker et al., 2017). This advanced endocrine resistance in tumors arises through enhanced GF singling, however recent evidence suggest that it may be due in part to adhesion to matrix and targeting matrix adhesion may sensitize cancer cells to primary therapy (Lazaro et al., 2013; Hanker et al., 2017). In accordance with this, stiff matrix and FAK activation are correlated with increased p-ERK (phosphorylated-extracellular signal-regulated kinase), a key regulator of pro-survival and proliferative pathways commonly active in the acquisition of endocrine resistance and disease progression (Gangadhara et al., 2016). The presence of increased FAK in luminal A subtype was suggestive of needing more aggressive therapy to lower the risk of breast cancers when FAK was observed (Almstedt et al., 2017). Prior studies that focused on HER2 amplified breast cancers showed that FAK inhibition in combination with trastuzumab (a HER2 specific antagonist) resulted in suppression of cellular growth and increased response to HER2 inhibition (Lazaro et al., 2013). Inhibition of FAK phosphorylation was also demonstrated to inhibit receptor negative breast cancer survival and migration (Woo et al., 2017). There is also a correlation to increased matrix concentration and activation of the FAK/MAPK intracellular signaling cascades (Provenzano et al., 2009). Furthermore, seeding of breast cancer cells on 3D matrix induces drug resistance to both endocrine therapies and HER2 inhibition; this resistance is observed to correlate with enhanced MAPK signaling (Provenzano et al., 2009; Gangadhara et al., 2016). This evidence provides a mechanism for drug resistance that is not dependent on GF signaling, as many integrin based and matrix-based therapies were developed and recently reviewed (Raab-Westphal et al., 2017). Despite the identification of integrin and matrix adhesion as targetable pathways, translating these adjuvant therapies to the clinic remains difficult. Many of these initial drug screens were tested on 2D culture and fail to incorporate native-like tumor microenvironments due to technical limitation. Thus, therapies should consider the role of ECM in cellular crosstalk and cancer cell interactions with their surrounding environment for disease outcome. Ideally, a correlation between known ECMs found in tumor tissue and activation of survival and proliferative pathways should be identified for better targeted therapies.

#### ECM-associated molecular modulators

Matrix architecture, varying degree of stiffness, traction force conferred by cytoskeleton and porosity of a tumor contribute to altered cancer cell behavior (Liu J. et al., 2012) via signaling cascades triggered by cell-ECM interactions. In addition to mechanotransduction in cancer microenvironments, a substantial number of soluble factors critical in tumor progression exist. MMPs aide cancer progression by degrading matrix proteins in stromal and basement membrane components, which act as signaling molecules upon sequestration (Figure 2, called matrikines; Akthar et al., 2015). TIMPs (tissue inhibitors of metalloproteinases) maintain the homeostasis of ECM by regulating the activity of MMPs. Additionally, collagen stiffness, concentration, and cross-linking were shown to regulate MMP activity in pancreatic cancer cells. Although it is not the case of breast cancer specifically, mechanotransduction pathways in cancer can alter MMP activity through integrin engagement and heparan sulfate proteoglycans (Haage and Schneider, 2014). Increased levels of TIMP-1 in primary tumors from a cohort of 176 patients correlated with a shorter overall survival in patients treated with chemotherapy prior to surgery (Dechaphunkul et al., 2012). In general, increased levels of TIMP-1 predict poor response to therapy and shorter patient survival. TIMP-3 expression is often blocked in cancers, thus increased levels of TIMP-3 resulted in better response to chemotherapy (Jackson et al., 2017).

Other molecular modulators include ECM proteins like fibronectin and hyaluronic acid (HA), which are less commonly evaluated but possess clinical prognostic and diagnostic correlations. FN isoform expression is poorly correlated with metastasis-free, overall survival of breast cancer patients (Bae et al., 2013; Fernandez-Garcia et al., 2014). HA is in higher amounts in the serum of patients with metastatic breast cancer compared to patients without signs of metastasis, clinically correlating HA with an aggressive breast cancer phenotype (Bae et al., 2013; Fernandez-Garcia et al., 2014; Karousou et al., 2014). In Table 1, we group ECM genes and proteins in association with individual hallmarks reported in the literature (See references in the Supplementary Information). Of note, while conventional tumor ECMs are primarily prepared with collagen type I-based scaffolds, collagens (types IV and VI), laminins (laminin-332 or other than Matrigel™-derived basement membrane proteins) and elastin (including elastin-derived polypeptides) are demonstrated to contribute to multiple hallmarks. In addition, further details in Tables 2–4 show the frequency of publications linking specific ECM-associated genes and proteins with both patient prognostic evaluations and certain drug resistances (Figure 1B).

**Table 2:** ECM-associated cohorts reported to influence patient prognostics and drug resistance^a^^,^ ^b^.

**ECM gene of interest**	**EXP(↑) positive outcome**	**Dependent on other cellular components with prognostic evaluation**	**Cancer type**
ADAMTS8	N	+ADAMTS8, −ADAMTS15	Breast
COL11A1	N	AEBP1, COL11A1, COL5A1, COL6A2, LOX, POSTN, SNAI2, THBS2, TIMP3, VCAN	Ovarian
COL12A1	Y	+ITGB1, +COL12A1	Breast
COL12A1	Y	TNKS1BP1, CPSF7, COL12A1	Breast
COL12A1	Drug resistance	COL1A1, COL5A2, COL12A1 and COL17A1	Ovarian
COL15A1	MUT = N	COL15A1, SRGAP1, SURF6, ABO	Ovarian
COL15A1	Drug resistance	ITGB1BP3, COL3A1, COL5A2, COL15A1, TGFBI, DCN, LUM, MATN2, POSTN and EGFL6	Ovarian
COL16A1	Drug resistance	COL1A2, COL12A1, COL21A1, LOX, TGFBI, LAMB1, EFEMP1, GPC3, SDC2, MGP, MMP3, and TIMP3	Ovarian
COL18A1	N	COL4A2, COL6A2, COL6A3, COL18A1	Liver
COL6A2	N	AEBP1, COL11A1, COL5A1, COL6A2, LOX, POSTN, SNAI2, THBS2, TIMP3, VCANS	Ovarian
CTHRC1	N	CTHRC1, Periostin	Ovarian
CTNNA1	N	CD97, CTNNA1, DLC1, HAPLN2, LAMA4, LPP, MFAP4	Breast
FN1	N	FN, MMP7, MMP9, MMP11, TIMP1, TIMP2	Breast
HAS1	N	HYAL1, HAS1	Bladder
HAS1	Y	HAS1, HAS2	Skin
LAMA1	N	LAMA1, LAMA2, LAMA3B, LAMA4, LAMB1, LAMC3	Breast
LAMA1	MUT = N	LAMA1, LAMA3, LAMB1, LAMB4	Gastric
LAMA2	AB. METH = N	GABRA1, LAMA2	Colon
LAMA3	METH = N	LAMA3, LAMB3	Bladder
LAMA3	Y	LAMA3, LAMB3, LAMC2	Prostate
MMP14	N	FSCN1, MMP14	Esophageal
MMP14	N	MMP2, MMP14, MMP9, MAXND	Oral
MMP15	N	MMP9, MMP15	Breast
MMP15	N	MMP15, MMP19	Colon
MMP2	Y	TIMP2, MMP	Colon
MMP7	N	GDF15, MMP7	Gastric
MMP9	N	TIMP2, MMP	Colon
PECAM1	N	TOP2A, GGH, PECAM1	Gastric
SELP	N	SELP, AKT1	Pancreatic
SPARC	Y	+NDRG1, -SPARC	Breast
SPG7	N	NOTCH2, ITPRIP, FRMD6, GFRA4, OSBPL9, CPXCR1, SORCS2, PDC, C12ORF66, SLC38A9, OR10H5, TRIP13, MRPL52, DUSP21, BRCA1, ELTD1, SPG7, LASS6, DUOX2	Colon
TIMP1	N	LCN2, TIMP1	Pancreatic
TIMP2	N	TIMP2, MMP	Colon
TIMP2	Y	TET1, TIMP2, TIMP3	Prostate, Breast
TIMP3	N	TSHR, RASSF1A, RARB2, DAPK, HMLH1, ATM, S100, P16, CTNNB1, GSTP1, CALCA, TIMP3, TGFßR2, THBS1, MINT1, CTNNB1, MT1G, PAK3, NISCH, DCC, AIM1, KIF1A.	Thyroid
VCAN	N	LCAN, VCAN	Colon^b^
Collagen type I	Drug resistance	MT1-MMP	Pancreatic
Collagen type IV	N	ELN-derived MMP12, COL4	Breast
Collagen type IV	N	COL4, ELN-derived peptides	Breast

a *Refer footnote from Table 1*.

b *EXP(↑) Positive Outcome, positive expression of the ECM gene of interest correlates with a positive patient outcome; MUT, mutation; AB. METH, abnormal methylation; METH, methylation; ±, expression up/down required for predicted patient outcome*.

**Table 3:** ECM-associated individual genes and proteins reported to influence patient prognostics and drug resistance^a^^,^ ^c^.

**Gene name**	**EXP(↑) positive outcome**	**Cancer type**
ADAMTS13	N	Liver
COL15A1	N	Liver
COL6A1	OXI. MOD = N	Ovarian
FN1	N	Renal
FN1	N	Head, Neck
HAS1	N	Breast
HAS1-3	N	Breast
ICAM1	N	Breast
ICAM1	N	Esophageal
LAMA1	N	Colon
LAMA2	Y	Breast
LAMA2	Y	Liver
LAMB1	N	Colon
MMP10	N	Colon
MMP16	N	Colon
MMP16	N	Gastric
MMP3	N	Breast
MMP3	N	Head, Neck
MMP3	N	Lung
MMP3	N	Ovarian
MMP3	N	Pancreatic, Breast, Lung
MMP7	N	Colon
MMP9	N	Colon
Myofibroblasts	Y	Pancreas
TIMP1	Y	Brain
TIMP1	N	Breast
TIMP1	N	Liver
TIMP2	N	Fibrosarcoma
TIMP3	HMETH = N	Gastric
VCAM1	N	Ovarian
VCAN	N	Colon
VCAN	N	Colon
VCAN	N	Ovarian^c^
Collagen type I	N	Breast
Collagen type I	N (STAGE DEP)	Colorectal

a *Refer footnote from Table 1*.

c *EXP(↑) Positive Outcome, positive expression of the ECM gene of interest correlates with a positive patient outcome; OXI. MOD, Oxidative modification; HMETH, hypermethylation; STAGE DEP, positive/negative expression of gene correlation with patient outcome dependent on the stage of tumor*.

**Table 4:** ECM-associated genes used as primary keywords in search parameters^a^.

**ECM protease**	**BM**	**ECM protease inhibitor**	**Transmembrane molecules**	**Collagens/Cell adhesion**
ADAMTS1	COL4A2	TIMP2	HAS1	COL12A1
ADAMTS13	LAMA1	TIMP3	ICAM1	COL15A1
ADAMTS8	LAMA2		MMP14	COL16A1
MMP1	LAMA3		MMP15	COL7A1
MMP10	LAMB1		MMP16	VCAN
MMP11	LAMC1		PECAM1	CTNNA1
MMP12	SPARC		SELE	CTNNB1
MMP13	COL6A1		SELL	
MMP2	COL6A2		SELP	
MMP3	COL8A1		SPG7	
MMP7			VCAM1	
MMP8				
MMP9				
SPG7				
TIMP1				

a *Refer footnote from Table 1*.

### Breast cancer microenvironments *in vitro* and *in vivo*

Despite an overwhelming amount of pre-clinical research on breast cancer microenvironments and chemotherapies, the likelihood of approval of an oncology drug after completing Phase 1 trials was roughly 5% during 2006–2015 (Thomas et al., 2016). This low efficiency is due in part to the inherent discrepancy between the current preclinical cancer models and their ability to mimic the native breast cancer microenvironment in patients. To date, no current preclinical model fully recapitulates the stromal, immune, architectural, physical, and molecular components of the native breast cancer microenvironment. Initial studies that strive to mimic the native breast cancer microenvironment utilized communication between breast cancer cells and the stroma through methods of co-culture. Co-culture methods are often limited to communication between only two cell types, which is too little to understand stromal communication. The immune component of 3D microenvironments is largely neglected when creating culture systems. The architecture and mechanical nature of the breast cancer microenvironment is only partially integrated, as many culture systems utilize ECM proteins in a spatially random manner. Rather, attempts were focused to attain similar tumor stiffness via modulating scaffold stiffness, while other biophysical properties (pore size, fiber alignment, etc.) and associated changes in ECM protein composition are rarely considered. Identification for differences in primary tumor matrix compared to metastatic seed matrix is rarely considered.

The widely used, practical but incompetent platform is the standard 2D culture with tissue-culture polystyrene (TCPS). This model has been a great starting point by providing a low-cost way to rapidly screen drugs and to correlate directly attributable changes with breast cancer cells *in vitro*. By simply taking 2D to 3D cell culture via gels of collagen type I or Matrigel™, noticeable changes are observed in cell morphology, growth rates, metabolism, and drug sensitivities (Gurski et al., 2017). Enhancing drug efficacy between 2D and 3D cultures is imperative as chemotherapies (paclitaxel, doxorubicin, and 5-fluorouracil) are less effective in drug sensitivity studies by simply changing the dimensionality of the platform (2D to 3D) or from *in vitro* to *in vivo* experiment (Imamura et al., 2015). Nonetheless, these rather simple 3D platforms cannot provide an appropriate match to physiologically relevant models (Breslin and O'Driscoll, 2016).

Physical properties of breast cancer cell microenvironments are also of importance to build predictable models to enhance anti-cancer therapeutics. Stiffness of a tumor is correlated to survival in patients and is key aspects of proliferation and metastasis in breast cancer (Schrader et al., 2011). The alignment of fibrous ECM proteins in the breast cancer microenvironment aids in the metastasis of cancer by providing a “highway” for cancer cells to migrate on (Egeblad et al., 2010). Increases in the amount of fibrous tissue in the breast increases breast density physically, which frequently appears in mammograms. Increased radiological density observed in mammograms is one a risk factor for developing breast cancer (Maskarinec et al., 2010). Currently, the most advanced preclinical models are patient derived xenograft (PDX) models, which involves the propagation of patient tumor biopsy in immunocompromised mice. PDXs include an intact ECM architecture and stromal component, making PDXs a remarkably powerful tool to predict cancer therapeutics (Cassidy et al., 2015). Despite such advantages, the stromal invasion by mouse cells over time leads to altered ECM composition and PDX models are incapable of discerning the individual contribution of cancer cells, stromal cells, and ECM (Cassidy et al., 2015). A recent study on genomic rearrangements (CNV, copy-number variation) in 543 PDX models representing 24 classes of cancer showed that expansive regions of CNVs were introduced into 60% of the PDXs after one tumor passage. After four tumor passages, 88% of PDX models displayed large CNV regions. PDX models undergo mouse-specific tumor evolution and their genome differs from the original patient tumor sample after extensive passaging (Ben-David et al., 2017). Thus, the goal of this review is to discuss creation of predictable preclinical models for breast cancer research by evaluating engineered extracellular microenvironments with molecular definition and modulation of physical properties through 3DBP.

### Other physicochemical and biological factors to mimic native breast cancer microenvironments

With new technologies emerging in the field of tissue engineering, engineers and cancer biologists collaborate to enhance current culture platforms to better mimic native-like breast cancer microenvironments. Technologies developed and emerged to recapitulate breast cancer microenvironments *in vitro* are summarized in Table 5.

**Table 5:** Comparison of 3D *in vitro* model platforms of breast cancer microenvironments.

**Model**	**Defining feature**	**Advantages**	**Disadvantages**	**Areas of interest**	**References**
Natural matrices	Matrix composed of naturally derived ECM proteins (collagen, laminin, HA, Matrigel™, fibrin) or polysaccharides (alginate, chitosan)	High biocompatibility, high adhesion properties, remodeled and modulated by cells, variable stiffness, including secreted ECMs	Batch-to-batch variability, complex molecular composition, uncontrolled degradation, spatially random without proper care	Fiber alignment, stiffness, multi-culture, hypoxia, formation of spheroids, invasion, migration, angiogenesis	Gu and Mooney, 2016; Pradhan et al., 2016; Regier et al., 2016; Roudsari and West, 2016
Synthetic matrices	Matrix composed of synthetic polymers (PEG, PLGA, PCL, polyurethane to name a few)	Highly tunable biophysical and biochemical properties	Poor cell adhesion, often difficult for cells to degrade, cytotoxicity	Fiber alignment, stiffness, co-culture, formation of spheroids, EMT, CSC generation, migration, angiogenesis	Gu and Mooney, 2016; Morgan et al., 2016; Pradhan et al., 2016; Roudsari and West, 2016; Samavedi and Joy, 2017
Composite matrices	Matrix composed of both synthetic and natural materials	Maintains high tunability of biophysical and biochemical properties with adjusted biocompatibility	Cytotoxicity, batch-to-batch variability, complex molecular composition, custom systems which promotes inaccessibility	Porosity, stiffness, co-culture, hypoxia, formation of spheroids, invasion, migration	Gu and Mooney, 2016; Pradhan et al., 2016; Samavedi and Joy, 2017; Yue et al., 2018
Spheroids	Self-arrange/assembly and proliferation into spherical shapes	Recapitulating early development of *in vivo* conditions, producible in other models	Reliance on spontaneous cell interaction	Multi-culture, vasculature, migration	Gu and Mooney, 2016; Morgan et al., 2016; Regier et al., 2016; Roudsari and West, 2016
3D microfluidics	Precise control over fluids, structure, and cells on the submillimeter scale	Very high spatial and temporal control, reduced sample volume, fluidic patterning of cells and matrix allowing close cell-cell contacts and complex geometries	Difficulty in maintaining continuous fluid flow, exaggeration of certain fluidic properties, advanced systems are inaccessible to most	Porosity, stiffness, multi-culture, formation of spheroids, invasion, chemotaxis, tissue patterning, vasculature, metastasis (extravasation, intravasation), “on-a-chip” technologies	Zervantonakis et al., 2012; Sackmann et al., 2014; Sung and Beebe, 2014; Gu and Mooney, 2016; Morgan et al., 2016; Regier et al., 2016
Perfusable tumor model	Introduction of continuous fluid flow akin to vasculature (incorporating multiple forms of bioreactors)	Ameliorating issue with transport problems in traditional culture by removing wastes and supplying oxygen and nutrients to cells	Lack of complete controls to transport problems	Co-culture, recellularization of scaffolds, vasculature	Mishra et al., 2015; Guller et al., 2016; Pence et al., 2017; Kulkarni et al., 2018

#### Perfusable tumor models

Incorporating a porous, perfusable scaffold to an *in vitro* bioreactor creates a new type of culture system named a perfusable tumor model (Fong et al., 2014). These models are used to effectively produce tumor-like structures, further developed to provide better insight into metastatic mechanisms. Perfusion-based tumor models offer tumorigenic cells cultured on scaffolds with a continuous flow of nutrients and oxygen while removing unnecessary byproducts of cell metabolism. Thus, these bioreactors are systematically tuned and automated to ensure cells experience optimal growth conditions. In perfusion-based 3D cancer cell culture, gene expressions of apoptosis and hypoxia are found to be comparable to tumor xenografts (Ma et al., 2012; Hirt et al., 2015). Perfusable tumor models were also used to simulate metastatic seeding of breast cancer cell lines in an acellular lung scaffold. Tumor nodules formed quickly within the perfused lung, showing high percentages of Ki-67-positive cells (a proliferation marker) and low percentage of Caspase 3-positive cells (an apoptosis marker). Histology of the tumor nodules matched the primary tumor type (Pence et al., 2017). MCF-7 and MDA-MB-231 spheroids were cultured on a perfusable tumor model and treated with a combination of hyperthermia and chemotherapy. The perfused spheroids displayed increased resistance to both the hyperthermia and combination therapies when compared to the spheroids cultured on the same scaffold, but not perfused. This increased resistance to therapy indicates the importance of constant perfusion in tumors (Kulkarni et al., 2018).

#### Composite biomaterials to modulate stiffness and biocompatibility

Stiffness is defined as the resistance of an object to deformation when a force is applied. As *in vitro* technology advances, creating scaffolds or tumor models with defined stiffness or matching stiffness of known tumor tissue become attractive strategies. Frequently, stiffness is recapitulated by incorporation of a natural, synthetic, or composite hydrogel. Natural matrices utilize ECM proteins such as collagen, laminin, HA, Matrigel™, fibrin or polysaccharides (alginate, chitosan) and synergize well with cells and are able to be degraded (Gu and Mooney, 2016; Pradhan et al., 2016; Regier et al., 2016; Roudsari and West, 2016). Synthetic matrices are often hydrogels composed of synthetic biocompatible polymers, polyacrylamide (McGrail et al., 2014), polycaprolactone (PCL) (Guiro et al., 2015), poly(ethylene glycol) (PEG) (Pradhan et al., 2017) to name a few, whose stiffness can be precisely tuned by concentration and multiple cross-linking chemistries. Synthetic matrices, however, can be cytotoxic in varying degrees to seeded or encapsulated cells due to the presence of initiators, catalysts or by-products and inherently incompatible with cells (Gu and Mooney, 2016). To increase biocompatibility of such synthetic polymers, whole-molecule ECM proteins are conjugated (termed composite matrices) to promote adhesion of cancer cells while maintaining stiffness of macroscopic scaffold (Gu and Mooney, 2016).

#### Direct and indirect communication via co-cultures

Ample evidence shows that the native breast cancer microenvironments are supported by stromal, vascular and immune components to aid proliferation and tumor progression (see section Stromal Component). One of the most accessible forms of co-culture *in vitro* utilizes conditioned media. Conditioned media contains the secretome of an effector cell type that is transferred onto a responder cell type (Regier et al., 2016). Another method is to utilize a permeable membrane (Slater et al., 2011) between two separate cultures allowing to communicate between the two different and separate cell types with or without direct contact. A trans-well assay is a relatively well-established co-culture model with a permeable membrane that physically separates two different cell types, while allowing to communicate via secretomes (Majety et al., 2015).

To effectively culture separate cell types together, microfluidic or spheroid models are used. Spheroids are in essence cells that self-assemble into a spherical shape. Multiple cell types can be incorporated into one spheroid creating a simple, robust 3D co-culture model (Gu and Mooney, 2016; Morgan et al., 2016). Spheroids of SUM159 breast cancer cells and 293T fibroblasts were created using the magnetic levitation method and treated with doxorubicin. The heterospheroids displayed increased doxorubicin resistance when compared to 2D control cultures. Additionally, the heterospheroids treated with doxorubicin displayed decreases in tumor size and density similar to xenograft-based tumors treated with doxorubicin (Jaganathan et al., 2014). The degree of spatial and temporal control offered by microfluidic models allows close cell-cell contacts and complex geometries to be constructed. With such complex patterns, multiple cell types are able to communicate and influence each other (Sung and Beebe, 2014; Regier et al., 2016). For instance, a microfluidics chamber is constructed to co-culture cancer cells and fibroblasts, demonstrating that the co-culture system enhances proliferation, paclitaxel resistance, and fibronectin expression relative to a mono-culture system (Jeong et al., 2016). Utilizing the precise patterning and control of microfluidics, 3D vascular networks can be constructed to examine how cancer cells extravasate (Chen et al., 2013; Jeon et al., 2015) and intravasate (Zervantonakis et al., 2012) during metastasis. These complex microfluidic models represent “tumor-on-a-chip” technologies which aims to mimic the cellular heterogeneity and structure of tumors (Sung and Beebe, 2014; Tsai et al., 2017).

Vascularization remains a very important step in tumor progression and there are 3D models available which seek to replicate this phenomenon. Utilizing a bioprinting method called laser direct-write, MDA-MB-231 breast cancer cells were co-cultured with fibroblasts and MCF-7 breast cancer cells by printing onto live rat mesentery. The *ex vivo* rat mesentery contained live blood vessels, lymphatic vessels, and interstitial cells. The thin slices of mesenteric tissue facilitate viewing with confocal microscopy. After 5 days of culture, mesentery sections where MDA-MB-231 were printed showed increased number of new capillary sprouts in comparison to mesentery sections without any printing (Phamduy et al., 2015; Burks et al., 2016). Vascularization during tumor progression was also modeled in a hydrogel composed of an internal collagen/colorectal cancer cell core and an external stromal cover composed of laminin, fibroblast, and endothelial cells. Vascular networks during co-culture formed cobblestone patterns with longer, but less interconnected vascular branches compared to vascular networks during stromal culture alone (Magdeldin et al., 2017).

Despite this ample evidence, most of recapitulated breast cancer microenvironments were formed by rather single constituent of ECM protein and simple alteration of scaffold stiffness. Animal models (e.g., PDXs) also showed to evolve over several passages. Attempts to build better breast cancer microenvironments included perfusable tumor models, modulating stiffness and biocompatibility and communication via cell-cell contact or secretomes. The next section adds more emerging technologies to better mimic the native breast cancer microenvironments.

## Revisiting the underdeveloped cues of breast cancer microenvironments

### Tumor and matrix stiffness

**Figure 3:**
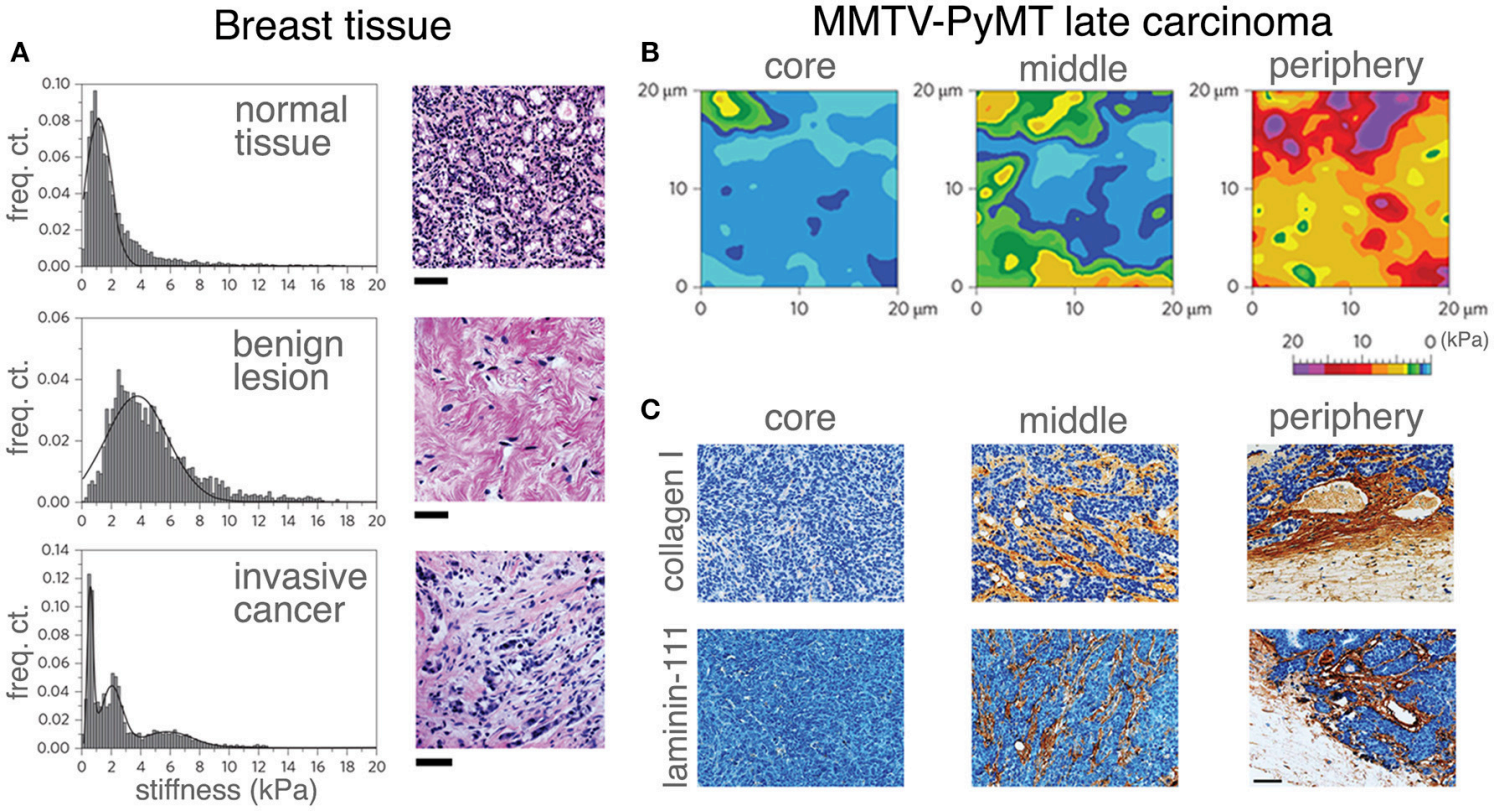
Correlating mechanical properties and ECM reorganization during human breast cancer progression. Stiffness distribution and respective H&E stained sections **(A)** of normal mammary gland tissue (top), benign lesion (middle) and malignant tumor (bottom). Stiffness distribution of normal breast tissue is unimodal and the histology shows the terminal ductal lobular unit of a normal mammary gland fenced by interstitial connective tissue. A benign lesion reveals a unimodal, but broader stiffness distribution with an increase in stiffness compared with the healthy sample. The histology of benign lesions reveals extensive fibrotic stroma interspersed with fibroblasts typical for fibroadenoma. Invasive cancer shows heterogeneous stiffness distribution with a characteristic soft peak, where the histology shows an invasive breast carcinoma with infiltrating nests of cancer cells that have evoked a dense fibrous tissue response. Scale bar applies to all images, 50 μm. The distinct ECM stiffness and structure of late MMTV-PyMT cancer was probed by atomic force microscopy **(B)** and immunohistochemistry **(C)**. Gradual stiffening from the core to the periphery was observed. Mechanical heterogeneity increased and is most extensive at the periphery **(B)**. While collagen type I and laminin-111 were virtually absent in soft tissue (the core), the heterogeneous presentation (brown staining) of collagen type I and laminin-111 was increased at the periphery as evidenced in **(C)**. Scale bar applies to all images, 50 μm. (**A–C** reproduced with permision from Plodinec et al., 2012).

The majority of stiffness studies are often performed using rat tail collagen type I (Mason et al., 2013; Branco da Cunha et al., 2014; Wei et al., 2015). The most common results are that increased matrix stiffness promotes an aggressive, more metastatic cancer cell. This is largely contributed to an increase in ECM deposition along with ECM cross-linking, usually by the lysyl oxidase family (Egeblad et al., 2010). Matrix stiffness modulation activates MAPK and AKT/PI3K pathways (Figure 2), which are both proliferation and survival pathways, respectively. Increased stiffness prompts cancer cells out of senescence and also promotes the TAZ (transcriptional coactivator with PDZ-binding motif) pathway, resulting in proliferating CSCs. Clinically, the stiffness of breast tissue can be used clinically as diagnostic and prognostic predictive measures. Of a cohort of 362 with breast cancer and 656 healthy patients, breast tissue stiffness was found to correlate with breast cancer risk (Boyd et al., 2014). This study was performed by estimating breast tissue stiffness from mammogram data. Additionally, a cohort of 55 patients with varying degrees of breast tissue stiffness was treated with chemotherapy and their response was measured. Patients with less stiff breast tissues responded better to chemotherapy than patients with stiffer breast tissues, indicating that stiffness can be a predictive tool for breast cancer patients (Hayashi et al., 2012). As tumors progress, cancer cells themselves begin to soften while increasing ECM content to contribute to a higher stiffness overall (Plodinec et al., 2012). During tumor progression, tissue stiffness is altered and expressions of various ECM proteins vary depending on location within the tumor (Figure 3). The MMTV-PyMT mouse model generates spontaneous breast cancer and spontaneous lung metastases and when injected with fibroblasts secreting CXCL12 can initiate matrices with varying degrees of stiffness within the tumor. The tumors were resected and the matrix stiffness was evaluated. The mice were continuously monitored to examine the effects of tumor stiffness on local and metastatic recurrence. Of note, metastasis was inversely correlated with tumor stiffness, suggesting that ECM properties and content beyond matrix stiffness contribute to metastatic progression (Fenner et al., 2014). In another report (Chaudhuri et al., 2014), alginate and reconstituted basement membrane composite scaffold can change stiffness and composition in a modular fashion. Stiffness alone can induce normal mammary cells into malignant, invasive, and non-growth restricted. The combination of stiffness and composition can modulate mechanotransduction pathways as well. In addition, cancer cells altered with an increasing stiffness profile may alter stromal interactions within the tumor as demonstrated recently using a co-culture of pre-adipocytes and breast cancer cells on a microwell array with variable stiffness. ECM stiffness increased as a direct result of this co-culture and the differentiation potential of the pre-adipocytes was decreased on stiffer hydrogels (Yue et al., 2018). While the role for stiff matrix has been well defined, alterations in matrix stiffness between the primary tumor and sites of metastasis are less well understood. In general, metastatic cells move from a stiff primary tumor to a soft tissue. Cellular adjustments to these new mechanical cues warrant investigation as they may provide insight to cell dormancy, drug resistance and time to recurrence. Preliminary studies evaluating this are seen in metastatic ovarian cancer where the cells prefer a softer matrix soil, demonstrating advantages of which cancer cells grow on a less stiff matrix (McGrail et al., 2014). For ovarian cancer, this mechanosensitivity is controlled by the Rho-ROCK pathway—a key actin rearrangement pathway. These data suggest that cancer cells have a mechanical preference depending on the stage, type, and subtype of cancer.

### Tumor density and porosity

**Figure 4:**
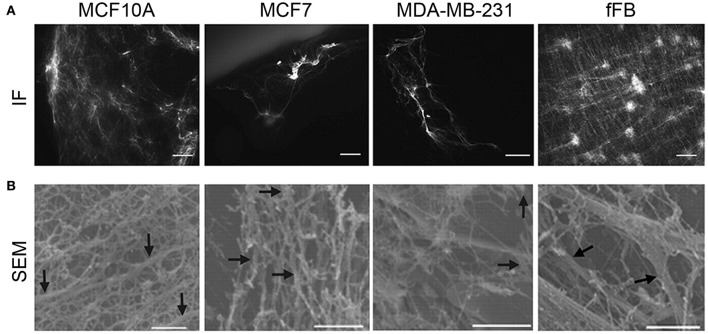
Correlating cell type and ECM architecture after decellularization. **(A)** Immunofluorescence (IF, scale bars, 50 μm) staining showed sparse distribution of ECM from multiple breast cancer cell lines, while abundant ECM deposition by human neonatal foreskin fibroblast (fFB). **(B)** To further visualize the deposition of ECM from the breast cancer cell lines, scanning electron microscopy (SEM, bottom row, scale bars 1 μm) was utilized. MCF10A shows organized, interconnected fiber morphology of ECM; MCF7 has a less organized arrangement of ECM fibers; MDA-MB-231 has a thin, sparse fiber morphology; fFB has a copious monolayer of ECM containing both large and thin-diameter fibers. ECM fibers (0.1- to 0.5 μm diameter) indicated by arrows. (**A,B** reproduced with permission from Hielscher et al., 2012).

As cancer progresses, tumor density increases similar to tumor stiffness. In breast cancer, tumor density affects estrogen receptor alpha (ERα) responses. When ERα-positive cells were cultured on high density matrices, the cross-talk with prolactin receptor pathway resulted in enhanced growth and insensitivity to hormone antagonists, while initiating collagen reorientation on the substrates (Barcus et al., 2015). Similar outcomes were found when this study was translated *in vivo* using a mouse model. Mice treated with an isoform of collagen type I displayed increased circulating tumor cells (CTCs), an increase in the number of lung metastases and reorientation of collagen type I (Barcus et al., 2017). Tumor density also correlates to changes in the metabolism of cancer cells. When cancer cells were grown on high density matrix of collagen type I, the attenuation of the oxygen consumption and glucose metabolism were observed (Morris et al., 2016). Breast tissue density is often found to be a risk gauge for developing breast cancer (Morris et al., 2016). As a tumor progresses and the ECM increases with in deposition and cross-linking, the density of a tumor also increases. Figure 4 shows immunofluorescent and scanning electron microscopy (SEM) results of ECM network organization (Hielscher et al., 2012). The immunofluorescent staining (Figure 4A) shows the distribution of ECM networks within various breast cancer cell lines, including MCF10A, MCF7, and MDA-MB-231, and compares it to a large deposition of ECM networks found with neonatal foreskin fibroblasts (fFB). Using SEM (Figure 4B), fibers in the ECM of the breast cancers and fFBs are both thin- and large-diameter ranging from 0.1 to 0.5 μm. The ECM fibers of MCF10A show a relatively higher organization with a variety of interconnected fibers while those of MCF7 show lower organization with less interconnection between the fibers. The ECM architecture of MDA-MB-231 is a relatively spare fiber network with thin-diameter fibers. In contrast, the ECM of fFB exhibits higher organization with both interconnection and a wider range in the diameter of the fibers. Features of the ECM have been known to affect tumor progression (Sadlonova et al., 2009), in which normal breast-associated fibroblasts and CAFs have a differential impact on breast cancer progression. CAFs have a more significant impact on the structure of the surrounding ECM (Fullar et al., 2015). Another study took a step further and showed which specific hallmarks of cancer are influenced by CAFs (Shiga et al., 2015). Investigating the influence of CAFs provided a glimpse into certain mechanisms involved in tumor progression, and further study on the influence of a larger range of ECM proteins and genes may end up providing great insight into multiple aspects of tumor progression. Another area of research that may generate greater understanding on tumor progression is on the effect of ECM porosity. It stands to reason that the porosity of a tumor decreases because of increased ECM deposition (Wozniak et al., 2003; Haeger et al., 2014), while the effect of tumor pore size has yet to be evaluated alone. The importance of porosity is suggested to demonstrate that the ability of a matrix to provide optimal cell-cell contact and to results in differences in secreted factors (Qazi et al., 2017) and the ability to control spatial regulation of cancer cells of understated factors such as CSC phenotype and cadherin-mediated effects.

### Cell migration and morphology in 3D ECM

Collagen constitutes a sizable portion of protein in the body, it is no surprise that many changes of collagen composition and orientation occur during cancer progression. Specific collagens of interest are fibrillar collagens type I, III, and V as well as non-fibrillar collagen type IV. Typically, collagens type I and III are increased and collagen type IV is decreased as cancer progresses. As cancer progresses, cancer cells will degrade the basement membrane of a tissue as cancer cells become more invasive, which explains the decrease in collagen type IV (Cavallo-Medved et al., 2009). With the increases in fibril collagens, a change in the alignment of collagens occurs in that normal curly/anisotropic fibril collagen thicken and linearize as tumors progresses in breast tissue (Provenzano et al., 2008; Conklin et al., 2011). This change increases the stiffness of the ECM, leading to the progression of breast cancer via activation of stiffness associated pathways (Egeblad et al., 2010). It can be inferred that linearized or aligned fibers provide roadways for invasive, aggressive breast cancer cells to migrate. In fact, this idea is also used to create so called apoptosis-sink for glioblastoma multiform (Jain et al., 2014). The sink is an extracortical drug-conjugated hydrogel with an intracoritcal PCL-nanofiber roadway for glioblastoma cells to migrate along. Many of the glioblastoma cells that had entered the engineered hydrogel underwent apoptosis *in vitro*. Implantation of the hydrogel sink into mice brain with glioblastoma tumors led to a decrease in tumor volume *in vivo*. While not breast cancer specifically, other engineering 3D platforms have shown cancer cell line changes in drug resistance as well (Fong et al., 2013). Of particular interest are Ewing sarcoma cells cultured and drugged on 3D electrospun PCL scaffolds, where Ewing sarcoma cells showed an increase in drug resistance to doxorubicin compared to *in vivo* xenograft models of the cell line.

Morphologies of breast cancer cells differ on aligned fibers vs. randomly assorted fibers. When breast cancer cells were cultured on electrospun PCL-scaffolds either with an aligned or a random orientation, the proliferation of MDA-MB-231 (a very aggressive, chemoresistant cell line) was attenuated on both aligned and random orientation scaffolds of electrospun fibers in comparison to standard 2D culture methods (Guiro et al., 2015). However, when MDA-MB-231 cells were cultured on randomly assorted fibrous scaffolds, the breast cancer cells displayed a more rounded morphology distinguished from the spread, spindle-like morphology of MDA-MB-231 in 2D culture. The spindle-like morphology of MDA-MB-231 was more conserved on aligned fibrous scaffolds. These data indicate that fiber alignment can dictate cell movement within tumors and cell morphology.

### ECM proteins–collagens, glycoproteins, and proteoglycans

The most common components of all ECMs are collagens with a total of 28 different isoforms. As discussed earlier in section Tumor and Matrix Stiffness. (Tumor and matrix stiffness), collagen stiffness is higher in breast cancer as breast cancer progresses to expedite invasion and movement of cancer cells (Wyckoff et al., 2007). Fibronectin is a glycoprotein that is strongly upregulated in breast cancer (Sottile and Hocking, 2002), specifically in metastasis and invasion. Fibronectin is expressed by CTCs (Raimondi et al., 2011), and promotes invasion via STAT3 pathway and metastasis via MAPK pathway (Qian et al., 2011; Balanis et al., 2013; Figure 2). Laminin consists of the majority of the non-collagenous basement membrane. Several laminin isoforms promote the progression of cancer often through affecting cancer cell adhesion, specifically integrin binding and E-cadherin expression (Zahir et al., 2003; Kwon et al., 2012). E-cadherin adhesion and matrix binding are closely linked cellular processes (Figure 2). When β-catenin dissociates from E-cadherin, phosphorylated β-catenin is degraded by proteasome. In contrast, non-phosphorylated β-catenin is translocated to the nucleus and functions as an activator for T-cell factor (TCF) transcription factors, resulting in adhesion and tumor development. ECM proteins or Wnt ligand can phosphorylate ILK, which in turn inhibits phosphorylation of GSK3β and activate Wnt target genes. Stabilizing mutations in the β-catenin N-terminal sequence were found in 25% of metaplastic breast cancers (Hayes et al., 2008). Increased cytoplasmic and nuclear β-catein levels were observed in 40% of primary breast cancers and correlated with poor prognosis and worse patient survival (Sormunen et al., 1999; Lin et al., 2000; Karayiannakis et al., 2001; Ryo et al., 2001; Ozaki et al., 2005; Prasad et al., 2008).

In addition to collagen, fibronectin and laminin, relatively underutilized ECM proteins, such as glycosaminoglycans, proteoglycans, and matricellular proteins, influence breast cancer development and progression. Syndecan (SDC) is a member of heparan sulfate proteoglycan (HSPG) family, where SDC-1 promote cancer cell proliferation (Maeda et al., 2004; Baba et al., 2006) and integrin-mediated binding for cell adhesion and migration (Beauvais and Rapraeger, 2003). The expression of SDC-1 changes from within the cancer cell to ectopically during EMT (Loussouarn et al., 2008), indicating that available SDC-1 within a scaffold could be useful for investigating cell migration. Another HSPG of interest are glypicans (GPC) whose isoforms have opposite effects. The most well-researched glypican is GPC-3, specifically for its tumor suppressor effects by inhibiting Insulin-like GF (IGF)/Wnt (Wingless-type MMTV integration site family) signaling and phosphoinositide 3-kinase (PI3K)/AKT signaling (Buchanan et al., 2010). Ectopic expression of GPC-3 shows decreases in proliferation of breast cancer cells both *in vitro* and *in vivo* (Peters et al., 2003), while GPC-1 is highly expressed in breast tumor compared to regular breast tissue. Lumican is a member of the small leucine-rich repeats proteoglycans (SLRPs). Specifically, low levels of lumican correlate to poorer outcome for patients treated with endocrine therapy (Troup et al., 2003). This could be due to lumican's suppressing effects on cancer cells through regulating ER functions. Lumican alters cancer progression by suppressing proliferation, migration, and invasion as well as upregulating epithelial markers and downregulating mesenchymal markers in an ER-independent fashion (Karamanou et al., 2017). Lumican is not likely to be incorporated into a synthetic scaffold but can be found on decellularized scaffolds (Jadalannagari et al., 2017; Muhamed et al., 2017). Lysyl oxidases (LOX) are enzymes which catalyze the cross-linking of fibrillar collagens and elastin. Increasing tumor cross-linking is directly related to increases in tumor stiffness, thus LOX is a key factor found in tumor progression. Clinically, LOX expression is correlated with a poor prognosis (Sakai et al., 2009). Direct inhibition of LOX yields reduced tumor size and stiffness, resulting in a delay in tumor progression (Levental et al., 2009). LOX-like protein 2 (LOXL2) is albeit non-traditional ECM cross-linking reagent in breast cancer metastasis to the lung. The LOXL2 association with EMT regulatory transcription factor Snail1 stabilizes Snail1 expression, promoting breast cancer cells to undergo EMT. Additionally, LOXL2-overexpressed cell lines demonstrate increased metastatic seeding from tail vein injection (Salvador et al., 2017). Betaglycan or transforming growth factor (TGF)- βIII receptor promotes tumor growth in TNBC (Jovanovic et al., 2014) and the shedding of its ectodomain suppresses TGF-beta signaling and breast cancer migration (Elderbroom et al., 2014).

One way to incorporate a majority of these obscure ECM proteins in a breast cancer microenvironment is to decellularize tumor tissue. Decellularization is a technique which aims to remove native cells within a tissue while maintaining the specific architecture and structural proteins. By decellularizing tumors, 3D models are created that can accurately replicate the complexity of *in vivo* ECM proteins with similar material properties (stiffness, density, pore size, fiber alignment, etc.) (Lu et al., 2014). When repopulating an acellular scaffold derived from a xenograft of pulmonary adenocarcinoma, MCF-7 proliferation was altered similar to *in vivo* rates. Additionally, secretions of IL-8, bFGF, and VEGF by MCF-7 were increased on the decellularized scaffold compared to cultures on 2D TCPS and Matrigel™ (Lu et al., 2014). A recent report from Dunne et al. shows that in decellularized adipose tissue scaffolds, MCF-7 and BT474 breast cancer cells lines proliferate at a slower rate similar to *in vivo* models and have increased drug resistance when compared to 2D models. These findings suggest decellularized ECM may provide a strong *in vitro* matrix model for screening drugs and profiling cancer (Dunne et al., 2014). Strangely, somewhat opposite results were found when culturing T47D and BT474 breast cancer cell lines on hydrogels consisting of decellularized, lyophilized mouse breast tissue, named tissue matrix scaffold (TMS). The proliferation rates of breast cancer cells on TMS were faster than a 3D collagen matrix, 3D laminin-rich matrix, and a 3D synthetic poly (lactic-*co*-glycolic acid) (PLGA) scaffold. Additionally, drug resistance was decreased on TMS when compared to the other 3D scaffolds (Rijal and Li, 2017). A perfusable decellularized murine lung model was used to grow various lung cell lines successfully as evidenced by forming nodules when perfused through a bioreactor connected to the decellularized lung. These nodules display similar qualities to both typical lung cancer and metastatic lung cancer (Mishra et al., 2015). Despite positive outcome, tumor decellularization remains an incomplete and very inaccessible option. Decellularized matrix cannot provide the stromal cues and all of the molecular factors that are part of the native tumor microenvironment. Tumor decellularization is dependent on access to relevant tissues and which comes with an inhibiting cost. Access to patient samples is inherently limited since the standard of care methods ensure any tissue removed during patient biopsy is used for treatment purpose, specifically pathological review (Morgan et al., 2016). The cost of relevant decellularized tumor is also due to the high cost of maintaining colonies of mice with PDXs (Pompili et al., 2016).

The proteins covered in this section are a small selection of ECM proteins that have been found or used in an *in vitro* 3D model for cancer research purposes. For a more detailed description of ECM proteins in breast cancer, readers are recommended to read the following two reviews (Hoye and Erler, 2016; Insua-Rodriguez and Oskarsson, 2016).

### Primary and secondary tumor site differences

By the time metastasis occurs, the primary tumor site becomes a hard place to survive and grow. The tumor and surrounding areas become exceedingly stiff as a result of desmoplasia. Little vascularization results in hypoxic conditions, leading to more vascularization and pathways involved with invasion and metastasis. Collagen fibers begin to orient themselves in different ways, specifically curly and anisotropic fibers begin to linearize and orient themselves perpendicular to the tumor boundary facilitating invasion and metastasis (Provenzano et al., 2008; Egeblad et al., 2010; Conklin et al., 2011). Aside from the normal structure and function of the tissue, the primary tumor often secretes factors into exosomes that can prime the secondary site for metastasis. These factors alter ECM composition at the secondary site, creating a favorable environment for metastatic seeding (Hoye and Erler, 2016). Proteomic data provided by the Matrisome Project have highlighted differences of ECM composition between primary and secondary tumor sites. Proteomic profiles of differential metastatic potential are included in the following references (Naba et al., 2012; Gocheva et al., 2017) with human colon, liver, and corresponding tumors (primary in colon, metastatic lesion in liver Naba et al., 2014). The samples in these cases were decellularized in milder conditions leaving a minimal amount of intracellular protein.

As cancer progresses in the tumor microenvironments, such progressive changes are distributed farther to the margins of tumors. These changes may prime the area for recurrence following treatment (mastectomy, radiation, etc.). The altered breast cancer microenvironment following these therapies is referred to as the remnant microenvironment. It is important to note that these changes can potentially be picked up by utilizing cell-specific prognostic biomarkers in a recent report (Casbas-Hernandez et al., 2015). Adjacent tissue can be altered to CSC-like phenotypes, specifically TNBC being the most altering based on patient tumor data (Atkinson et al., 2013). Considering CSCs reinitiate tumor formation following primary therapy, CSCs play a crucial role in the remnant microenvironment. For example, when hepatocellular carcinoma cells are treated with chemotherapy and cultured on either stiff or compliant matrices, cancer cells cultured on soft substrates show a clone-initiating ability and present a CSC phenotype, as evidenced by significant increases of pluripotency transcription factors OCT4 and NANOG. This suggests that following chemotherapy, a stem-like quality of metastatic tumor cells found in less stiff microenvironments (Schrader et al., 2011).

Tumor stiffness is correlated with cancer progression and *in vitro* studies with collagen-only scaffolds at increasing stiffness shows higher drug resistance and proliferation. However, this is a simplistic view in that such increased stiffness does not account for cell migration from primary tumor (stiff) to new metastatic site (soft), where a soft matrix may induce cell dormancy and facilitate CSC phenotype, leading to recurrence and loss of response to primary therapy at metastatic sites. A few preliminary studies have initiated these evaluations in organs outside of breast tissues. For instance, metastatic ovarian cancer cell lines (e.g., SKOV-3) showed a correlation between drug resistance and decreased focal adhesion formation (McGrail et al., 2015), where decreased focal adhesions are indicative of cells cultured on a softer matrix (Yeh et al., 2017). These results support the claim that metastatic ovarian cell lines prefer a softer substrate (McGrail et al., 2014). Together, the movement of cells from primary tumor to soft surrounding matrix may lead to loss of focal adhesion and provide insight into novel mechanisms of drug resistance at metastatic sites (McGrail et al., 2014). For instance, breast cancer cells often metastasize to the brain, lung, and liver. All of these metastatic sites are softer than primary breast tumors. While not yet demonstrated, the movement of breast cancer cells from a stiff environment to a soft tissue may provide mechanism for cell dormancy and drug resistance. The exception to this scenario is breast cancer metastasis to bone, where drug resistance may be facilitated by the milieu of growth factors and more rigid matrix in the new environment (Cox and Erler, 2011).

In this section, we reviewed the biophysical microenvironments (stiffness, density and porosity) of breast tissue of patients were altered during breast cancer progression and chemotherapy. This alteration is a risk gauge for breast cancer stages. As breast cancer progresses, cells in primary and secondary (metastatic lesion) experienced different stimuli and responses. In the following two sections, we will focus on breast cancer microenvironments with biochemical factors (section Biochemical Properties to Further Develop Breast Cancer Microenvironments) and with 3DBP (section 3D Bioprinting).

## Biochemical properties to further develop breast cancer microenvironments

In breast cancer microenvironments, biochemical factors are stimulated by tissue-resident cells to secrete soluble factors. For a synthetic scaffold to properly mimic the breast cancer microenvironment, the proper composition of the soluble factors needs to be added to guide breast cancer cell behavior. The main biochemical factors include ECM proteins along with enzymes responsible for ECM remodeling (MMP or TIMP) and soluble factors accompanied with ECM proteins (GFs).

### Matrix-associated proteins responsible for remodeling breast cancer microenvironments

Exploring the critical ECM and matrix-associated proteins aids in understanding why they should be an integral component in an *in vitro* matrix. Cancer-associated inflammatory cells produce cytokines, chemokines, MMPs, and other factors to mediate carcinogenesis and metastasis in lungs (Mao et al., 2013; Shi et al., 2015). A biomimetic scaffold devoid of key factors results in an inaccurate model for *ex vivo* experiments. For example, cytokines are important in intercellular communications and regulate CSC phenotype (Ye et al., 2014). Chemokines are key components of cancer-related inflammation via leukocyte recruitment and function, cell senescence, tumor cell proliferation and survival, tumor progression, invasion, and metastasis (Camnitz et al., 2012). Some examples include TGF- α/β guiding tumor-adipose cell interactions and CCL2/5 for tumor-tumor-associated macrophage interaction. MMPs are involved in all stages of cancer progression, including tumor proliferation, adhesion, migration, and differentiation to name a few (Siefert and Sarkar, 2012; Mao et al., 2013). Activity of MMPs plays an important role in the invasion and extravasation of breast cancer cells, serving as biomarkers of progression and metastasis for tumor cells to invade the surrounding tissues. MMP-7/9/12 (tumor-associated macrophages), MMP-9 (embryonic cells), MMP-11 (adipose cells) and a myriad of MMPs are known to interact with CAFs, while also causing a transition of mature stromal cells to tumor cells (Mao et al., 2013). The large amount of research into the interaction between MMPs and various cells located in the breast cancer microenvironment impresses the importance of the addition of signaling between key stromal cells into an engineered native-like breast cancer microenvironments.

### Soluble factors to direct breast cancer cell behaviors

Hormones that influence tumor growth are delivered via the blood stream and create a gradient within the microenvironment. These hormones include estrogens, which strongly influence breast cancer development, and testosterones, which target prostate carcinoma progression (Thoma et al., 2014). Some immunosuppressive myeloid lineages promote angiogenesis through the secretion of soluble GFs. Vascular endothelial growth factor (VEGF), fibroblast growth factor (FGF), and TGF-β are three important GFs that promote this angiogenesis (Motz and Coukos, 2011). To gain trans-endothelial entrance, cells secrete MMP-1/2/3/10, TGF-β, and VEGF. Leading cancer cells enhance vasculature permeability particularly at the site of extravasation, where normal endothelial cells are organized (Wan et al., 2013). In the case of TNBC, cancer metastasis involves tumor microenvironment factors as peripheral signals including epidermal growth factor (EGF) and insulin-like growth factor I (IGF-I) at distant tumor sites (Castano et al., 2013). To increase the expression of transcription factors associated with pluripotency, proliferation, and phenotypic transition, EGF and IGF-I must be available in the tumor microenvironment. Combinatorial therapy to target EGF and IGF-I signaling prevents metastatic growth, suggesting plasticity and recurrence rates are dictated by host systemic factors and potentially a candidate therapy for patients (Castano et al., 2013).

Establishing the importance of the biochemical factors found in the breast cancer microenvironment expresses the need for a more accurate *in vitro* model. The complexity of the breast cancer microenvironment, paired with the lack of accurate models further proves why more research is required. All the various biochemical components have their own role in the tumor life-cycle, thus finding ways to effectively apply them to a synthetic scaffold should be the focus moving forward.

### Natural and synthetic biomaterials to modulate breast cancer microenvironments

To engineer breast cancer microenvironments, natural and synthetic biomaterials are required to create a scaffold that mimics the breast cancer ECM. Natural biomaterials such as alginate, collagen type I, reconstituted basement membrane (Matrigel™), laminin, and HA were the first and most commonly used materials due to their cytocompatibility, intrinsic cell adhesion properties, and ease of remodeling (Elsdale and Bard, 1972). Synthetic materials include polyacrylamide (PA), polydimethylsiloxane (PDMS), poly(ethylene glycol) (PEG), and poly(lactide-co-glycolic acid) (PLGA) provide more precise control of mechanical properties but lack cell adhesion and cytocompatibility (Gu and Mooney, 2016). In a recent review (Leggett et al., 2017), models for breast cancer ECM can comprise of individual ECM component to build the scaffolds, but more commonly includes a combination of multiple ECM components. For example, collagens and reconstituted basement membrane are commonly used together (Nguyen-Ngoc et al., 2012; Ahmadzadeh et al., 2017; Carey et al., 2017; Guzman et al., 2017). It is already well documented that traditional 2D and *in vivo* animal models do not adequately capture the breast cancer ECM (Infanger et al., 2013; Gill and West, 2014; Song et al., 2014). The inability to properly model the breast cancer ECM created a gap in the modeling of breast cancer ECM.

To confer biological features onto scaffolds, several biomaterials are functionalized with biochemical cues or presented to breast cancer cells in the engineered microenvironments. PEG hydrogels are functionalized with integrin-binding arginine-glycine-aspartic acid (RGD) peptides to enhance breast cancer cell attachment and with MMP-sensitive peptides to promote 3D epithelial morphogenesis of lung adenocarcinoma (Gill et al., 2012). Additionally, collagen and reconstituted basement membrane hydrogels are employed to study tumor-induced angiogenesis by co-culturing various types of cancer cells with human umbilical vein endothelial cells (Cross et al., 2010; Seano et al., 2013). Alternatively, it was shown that melanoma cells cultured in 3D models become more resistant to immune cells, compared to 2D models (Hirt et al., 2014). Other studies include alginate-chitosan hydrogels to study the interactions between prostate cancer and lymphocytes (Florczyk et al., 2012, 2013). Using temperature-sensitive Pluronic F127 and gelatin methacrylate hydrogels, angiogenesis is mimicked within vascularized tissue models (Kolesky et al., 2014). With RGD-modified alginate hydrogels and collagen type I within microfabricated PDMS scaffold, hypoxic 3D hydrogels were created to assess tumor angiogenesis (Verbridge et al., 2010). To better understand the changes of breast cancer microenvironments due to tumor progression, Matrigel™-collagen hydrogels were initially constructed to determine the correlation between matrix stiffness and an induced malignant phenotype (Levental et al., 2009). Recent improvements allow for the control of the hydrogel stiffness using alginate instead of collagen. Because alginate can be ionically crosslinked with CaCl_2_, the stiffness could be controlled simply by altering alginate or CaCl_2_ concentration (Chaudhuri et al., 2014) without reinventing cross-linking chemistry.

While the presented studies have successfully improved the breast cancer ECM model, they still lack the ability to spatially control biophysical properties of candidate scaffold for suitable breast cancer microenvironments. It is already well established that the breast cancer ECM is highly heterogeneous and the inability to control the spatial deposition of biomaterials reduces the accuracy of any model. The highly-organized patterning of vasculature, cells in their stroma, corresponding architecture of ECMs are yet readily available. With specific patterning via high-precision fabrication or 3DBP technologies, the growth and recapitulation of breast cancer microenvironments with the highly-organized manner found in natural tissue is feasible.

## 3D bioprinting

### 2D to 3D scaffolding

While 2D modeling is a common starting point for most cancer models, its simplicity limits applicability for complex, native-like breast cancer microenvironments. From an engineering point-of-view the paradigm shift from 2D to 3D can be challenging but allows for more physiologically relevant models. A 3D microenvironment provides a physical barrier to processes such as spreading, proliferation, invasion, and migration that is not outstandingly present during culture on 2D surfaces (Lee and Chaudhuri, 2017). Traditionally, modeling 3D microenvironments refers to using *in vitro* scaffolds or *in vivo* animal models. While using 3D scaffolds or animal models are more accurate in modeling a breast cancer ECM than 2D surfaces, there are still disadvantages in their use. In a recent review, the authors produce a comprehensive view on *in vitro* tumor models providing advantages and disadvantages for selecting the right platform (Katt et al., 2016). For example, the impact of the dimensionality is evidenced by the data showing that cells cultured in 2D vs. 3D showed drastic changes in gene expression (Breslin and O'Driscoll, 2016). Initial attempts to create 3D models included spheroid formation, simple formation of a matrix in a spherical shape to encapsulate cells (Wang et al., 2014; Asghar et al., 2015; Zhang et al., 2016), hanging-drop method (HD) using mechanical or automated methods (inkjet printing), and electrospinning. HD, inkjet printing, and electrospinning print 3D architectures that allows cells to grow and mature based on the surrounding environment. Each of the different methods utilizes different techniques and feeds material to produce the 3D structures. Comparison between some of these technologies is detailed here (Knowlton et al., 2015; Peng et al., 2016).

One of disadvantages of HD, inkjet printing, and electrospinning, is the lack of control over the structural architecture of the 3D microenvironments. This limitation results in stiffness, pore size, and other physical characteristics having to be adjusted solely based on chemical composition and cross-linking of feed biomaterials (Peng et al., 2016). In addition, the HD method is found to have a high level of difficulty in fabrication because the spheroids are initially formed by manually pipetting. Furthermore, the pipetting also makes it difficult to have a significant level of replicability in the spheroid size and shape. Eventually the HD method is automated but the replicability and applicability is still undesirable when compared to other methods (Ozbolat et al., 2017). From the basic understanding of the HD method, advancements in the delivery of the droplets lead to the inkjet method. The inkjet method utilizes computer-aided programs to deposit spheroid droplets on a previously designed pattern with great accuracy (Boland et al., 2006). Electrospinning also has similar issues with difficulty of use and the applicability of the produced biomaterials. Concerns over toxicity from the spinning processing create a major drawback for clinical application. Due to the need for high electrical fields and harsh solvents, it is a challenging method to maintain native protein function (Hinderer et al., 2016). Very few publications focus on functional electrospinning of ECM molecules (Hinderer et al., 2012) while a clinical translation has yet to be demonstrated. HD, inkjet printing, and electrospinning lack the potential for scalability and ease of use in future biomedical applications, making it difficult for them to succeed. The disadvantages inspire researchers to develop more efficient and controllable bioprinting methods with 3D context.

### Types of 3DBP applicable to fabricate breast cancer microenvironments

3D printing is a widely used additive manufacturing process that deposits materials on a layer by layer basis to produce complex 3D architecture. Historically, 3D printing has been around since the early 2000's and advancements in printer design increase their use and range of applications (Svensson et al., 2011). Traditional 3D printing methods utilize specialty plastics/polymers and computer-aided software to design and create objects. From this platform, the existing electrical and mechanical components are adapted for the extrusion or delivery method, which leads to the development of 3DBP. While there are many different types of 3DBP, the extrusion or delivery method distinguishes each method from one another. The three widely used methods are extrusion-based, inkjet-based, and laser-assisted bioprinting. Each of the three methods is not equal but is application specific. In Table 6, common bioprinting technologies are compared using metrics that are key to determine which technology would be best suited for developing a breast cancer microenvironment model (Knowlton et al., 2015; Peng et al., 2016). From the table, there is no clear favorite bioprinting technology, but choosing a desirable bioprinting method depends on the limitations of system being developed and available biomaterials for the chosen 3DBP method.

**Table 6:** Comparison of common 3DBP technologies^d^.

**Performance metric**	**Extrusion-based bioprinting (EBB)**	**Laser-assisted bioprinting (LAB)**	**Inkjet-based bioprinting (IBB)**	**References**
Throughput	Medium	Low to Medium	High	Tasoglu and Demirci, 2013
Droplet size	5 μm to millimeters wide	>20–80 μm	50–300 μm	Guillemot et al., 2010; Tasoglu and Demirci, 2013; Murphy and Atala, 2014
Spatial Resolution	Medium	Medium to high	Medium	Tasoglu and Demirci, 2013
Material/hydrogel viscosity	30 mPa.s to >600 kPa.s	1–300 mPa.s	<10 mPa.s	Chang et al., 2011; Murphy and Atala, 2014
Gelation method	Chemical, ionic, enzymatic, photocrosslinking, shear thinning, thermal, pH	Ionic	Ionic, enzymatic, photocrosslinking, thermal	Malda et al., 2013
Gelation speed	Medium	High	High	Malda et al., 2013
Print/fabrication speed	High	Low	Medium	Malda et al., 2013
Printer cost	Medium	High	Low	Murphy and Atala, 2014

d *Table adapted with permission from Knowlton et al. (2015)*.

#### Extrusion-based bioprinting

Extrusion-based bioprinting (EBB) generally uses more viscous bioinks because syringes dispense the bioink into a cross-linking chemical or a collector plate. Generally, bioinks used for EBB have three stages of viscoelastic nature: a) initial bioink formulation of polymers, cells, and cross-linkers, b) lightly cross-linked bioink, and c) finally a robust heavily cross-linked gel (Chimene et al., 2016). Ideal properties for partially cross-linked (Skardal et al., 2010; Boere et al., 2015; Rutz et al., 2015) and shear-thinning (Cohen et al., 2006; Loozen et al., 2013) gel-phase inks are already identified. EBB uses a mechanical mechanism dispensing the ink, layer-by-layer, to produce the 3D construct. The three most used EBB methods are pneumatic (pressure), piezoelectric (solenoid), and screw-driven (motor). All the methods provide the same product but utilize different mechanical methods to extrude the bioink. While most of the methods is practically interchangeable, personal preference can be a deciding factor. EBB prints layered structures with a greater deposition, providing print speed suitable for easier scalability in a short period of time, when compared to other printing methods (Ozbolat and Hospodiuk, 2016). The use of EBB is relatively easy to implement because it uses CAD–ssisted modeling to develop and print complex structures. This allows for individuals with limited training to operate the instrument efficiently. The main limitation of EBB is the slow print speeds and long print times associated with printing scaffolds.

#### Laser-assisted bioprinting

Laser-assisted bioprinting (LAB) is initially introduced as stereolithography and is similar to inkjet-based bioprinting (IBB) since LAB uses a less viscous bioink to create the spheroids or to directly write onto the tissue (Burks et al., 2016). The broad label of LAB can be further defined by laser-guided direct writing (Odde and Renn, 1999, 2000) or modified laser-induced forward transfer (Ringeisen et al., 2002, 2004). The process is still expensive because of the need for laser equipment and the application of the laser. Furthermore, the overall complexity of the instrument makes it difficult for any user to easily setup and operate in any setting. The example of laser direct-write onto living tissues explains a complicated method of bioprinting that takes a long time to complete the final product while posing potential threats to patient and user safety. While picking the optimal bioprinting method is not trivial, choosing the right materials and their compositions is more challenging. Based on previous reviews on bioprinting (Zhang et al., 2016), selecting a right formulation and biophysical properties of the bioink is a critical step toward the success of 3D bio-printed cancer microenvironments. While each of the printing methods has their advantages and disadvantages, the bioink employed in a specific printing method can determine the survivability and the key hallmarks (Figure 1) for specific types of cancer.

#### Inkjet-based bioprinting

Inkjet-based bioprinting (IBB), based upon inkjet printer technology, requires a less viscous bioink to prevent clogging of the cartridges. IBB is also regarded as the advancement of the HD method originally used to develop spheroids. Using this precursor method, users can print scaffold structures by layering the individual spheroids into a scaffold. Like EBB, IBB uses CAD–assisted modeling to print the desired object. However, the inkjet method is shown to struggle with complex 3D structures and the ability to layer bioink on top of the previous layer. Because of this issue, inkjet bioprinting produces a complex 2D object rather than a true 3-D scaffold, when compared to EBB.

### 3DBP in modeling breast cancer ECM

Information and research into biomechanical properties of the ECM are limited when synthetic scaffolds are employed for modeling. Biomechanical properties are shown to play a very important part in modulation the ECM and cell progression (Hinton et al., 2015; Laronda et al., 2017; Trachtenberg et al., 2017). Due to the limitations of previous bioprinting methods (Knowlton et al., 2015), the architecture of the scaffold is inadvertently neglected because methods such as spheroid formation and inkjet printing have limited capabilities. 3DBP methods, such as EBB, regulate the pore size in each layer printed for a scaffold, allowing for the fabrication of 3D gradient structures (Sobral et al., 2011; Trachtenberg et al., 2014). Due to their complex organization of cells and varying intratumoral permeability, diffusion barriers for mass transport and drug delivery varies (Paulsen and Miller, 2015). Thus, careful design of the architecture is required to accurately model breast cancer ECM. Of the 3DBP methods, EBB shows the most potential for precise architecture control. Factors of fluid shear stress and macro-scale architecture have synergistic effects on tumor phenotype, protein expression, and cell proliferation (Trachtenberg et al., 2017). Furthermore, previous studies show that hydrogel architecture including fiber density, size (length and width), and stress relaxation in addition to hypoxic gradients increases sarcoma cell migration (Lewis et al., 2017). Stress relaxation and elastic modulus of the ECM also regulate cellular responses (McKinnon et al., 2014) including cell motility (Lo et al., 2000), MSC differentiation (Chaudhuri et al., 2016), and ECM sensing capability of the cells (Carey et al., 2016).

#### Examples of 3DBP to model breast cancer ECM

**Figure 5:**
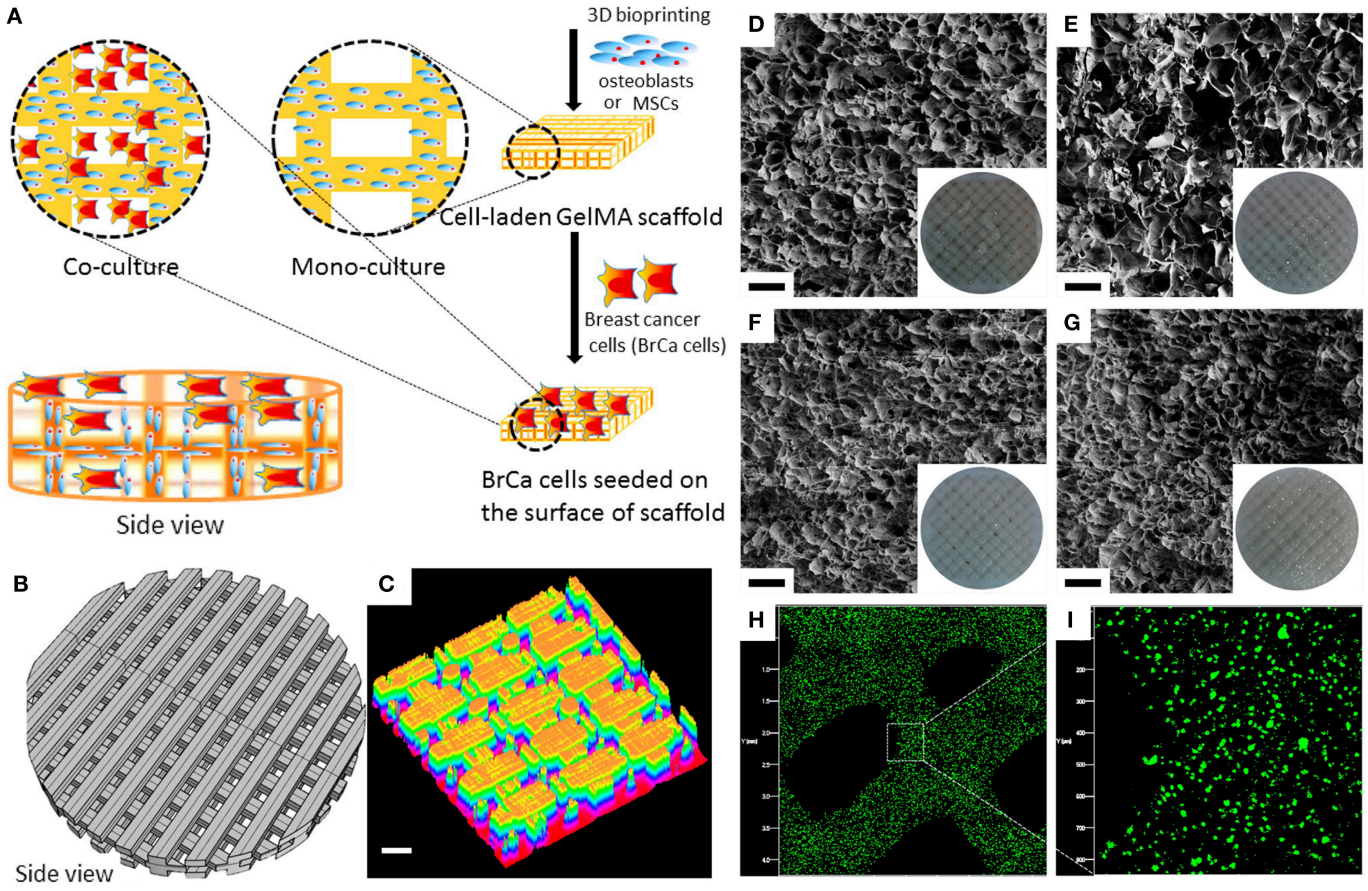
An example of 3DBP breast cancer microenvironments. **(A)** Schematic diagram of direct, 3D bioprinted, cell-laden bone matrix as a biomimetic model for a breast cancer metastasis study. **(B)** CAD model of the 3D matrix. **(C)** 3D surface plot of the bioprinted matrix. Scale bar: 200 μm. **(D–G)** Scanning electron micrographs (cross-sectional view) of porous matrices: **(D)** 10% GelMA, **(E)** 10% GelMA + nHA, **(F)** 15% GelMA, and **(G)** 15% GelMA+nHA, respectively. Scale bar: 100 μm. The inset images are photographs of the corresponding matrices. **(H,I)** Fluorescence micrographs of the 3D bioprinted MSC-laden 10% GelMA matrix; 3D bioprinted cells were prelabeled by Cell Tracker Green CMFDA dye. GelMA; gelatin methacrylate (**A–I** reproduced with permission from Zhou et al., 2016).

In a recent study (Figure 5), 3DBP was utilized to model interactions between breast cancer cells and bone stromal cells (Zhou et al., 2016). Scaffolds were developed using a tabletop stereolithography 3DBP and gelatin methacrylate hydrogel with nanocrystalline hydroxyapatite (nHA). This study printed MSCs or human osteoblasts-laden bioinks to develop the cell-laden scaffold and initiated co-cultures with breast cancer cells. Co-cultured MSCs/osteoblasts and breast cancer cells on the 3DBP scaffold showed increased VEGF secretion and enhanced growth of the breast cancer cells, while inhibiting proliferation of MSCs/osteoblasts. The group did not specify controlling the pore size or layer thickness of the scaffolds but altered the scaffold composition (10 and 15% of gelatin methacrylate with and without nHA (Figure 5). Nowhere did they report the response due to varying the size parameters and only focused on the swelling ratio and rheological properties. While the two properties are a function of pore size and layer height, it would be interesting to see further breast cancer cell response or metastatic behavior in bone microenvironments based on the biophysical properties.

A recent review summarized 3D printed *in vitro* cancer models, highlighting advancements made using spheroids, mono-culture, and co-culture applications (Samavedi and Joy, 2017). Ling et al. investigated tumor spheroid formation using MCF-7s encapsulated in sacrificial gelatin arrays. Later, the arrays were used to fabricate PEG-dimethacrylate concave wells (Ling et al., 2015). The combination of gelatin and PEG allowed for a single tumor spheroid formation step, eliminating the need for an additional seeding step. In another recent study, concave PEG-diacrylate hydrogel microstructures enabled the development of single BT474 breast cancer spheroids using a 3D projection printing technology (Hribar et al., 2015). In both studies, hypoxic cores and necrosis was demonstrated. Dai et al. showed discrepancies between a 2D and 3D drug resistance model for temozolomide. By forming glioma spheroids using a gelatin-alginate-fibrinogen bioink, breast cancer cells in 3D observed a higher drug resistance than those in 2D culture (Dai et al., 2016). Two other studies used PEG-based bioinks to create hydrogels with tunable stiffness showing a significant difference between 2D and 3D microstructures (Soman et al., 2012; Huang et al., 2014). While there are many examples of 3D printing synthetic bioinks for breast cancer modeling, there are still very few 3DBP models that successfully use natural bioinks. Matrigel™ (Rizvi et al., 2011; Xu et al., 2011), gelatin (Zhao et al., 2014; Dai et al., 2016), or alginate (Zhao et al., 2014; Dai et al., 2016) are commonly used for 3DBP models, but a gap exists in reported 3DBP employing important breast cancer-associated ECM proteins such as collagens type I, III, fibronectin, laminin, other glycoproteins, and proteoglycans. Incorporation of the breast cancer-associated ECM proteins as biochemical cues with the spatial acuity controlled by 3DBP will fill the current gap of bioinks to recreate native-like breast cancer microenvironments. Furthermore, the adaptability of 3DBP allows for future studies to investigate breast cancer cell behaviors and post-metastatic progression using scaffolds with precisely engineered biophysical properties with an easy modification of the native-like breast cancer microenvironments.

3DBP offers the ability to easily tailor scaffolds for *in vitro* modeling of the breast cancer ECM. Adjusting the pore size, fiber density, and orientation of the layers shows to alter cellular responses responsible for tumor progression (Laronda et al., 2017; Lewis et al., 2017). A current 3DBP technology, freeform reversible embedding of suspended hydrogels (FRESH), is important for printing advanced architecture and shows high potentials for various biomedical applications (Hinton et al., 2015). Other applicable technologies include development of bioprinted *in vitro* breast cancer co-cultured model (Leonard and Godin, 2016), bioprinting HeLa cells with gelatin/fibrinogen hydrogel to find differences of MMP expression vs. 2D models (Zhao et al., 2014), and even bioprinting a brain tumor model by extruding glioma stem cells-laden hydrogel (Dai et al., 2016). The inclusion of spatial control through 3DBP with key biochemical factors will have created a new and more precise form of the native-like breast cancer microenvironments. With the advancements presented here, future directions can be made in the field of personalized cancer research, specifically therapeutic drug testing and reproducing pre/post-metastatic microenvironments to effectively capture and expand CTCs.

## Outlook and summary

Research on breast cancer has advanced over the several decades and we reached several milestones in the application of therapeutics, such as chimeric antigen receptor (CAR) T-cell therapy. However, lacking is physiologically relevant and competent *in vitro* breast cancer models to expedite drug screening trials and to widen available therapeutics with improved predictable powers. Now, pitfalls of 2D culture is no longer questioned and efforts are focused on providing better defined, breast cancer microenvironments to test many novel hypotheses. ECM is at the center of this idea, allowing us to control breast cancer cell behavior by signaling directly via ECM proteins, GFs sequestered/tethered by ECM as well as biophysical properties of the ECM. Greater controls of the ECM structure of *in vitro* models could result in a higher sensitivity and specificity when testing hypotheses. Difficult questions that could only be partially answered—or not answered at all—with *in vivo* methods may be better answered using these new engineered model platforms. Furthermore, incorporation of stromal support cells and physiologically relevant ECM could provide accurate, reliable 3D breast cancer microenvironment models, allowing us to better understand how specific ECM molecules direct the expression and activation of immune components and therapies like PD-L1 inhibitors. Before these specific questions are answered, the central question is how we handle these multifactorial inputs with best engineering methods. Here, we proposed to use 3DBP of ECM proteins via EBB in the absence of toxic chemicals (as required in electrospinning) or radicals and phototoxicity (as required in photo cross-linking). Rich knowledge of synthetic, natural and composite biomaterials is accumulated, conferring flexibility of materials selection for 3DBP.

3DBP is an additive production of biomaterials, which emerges recently to fabricate tissues and organs. Printing resolution is at scales comparable to cell body, enabling modulation of extracellular signals to cells by continuous multi-material fabrication at micrometer scale. Evolving bioink formulations makes 3DBP a more accessible option to prepare an engineered breast cancer microenvironment for breast cancer research in comparison to organs-on-chips or specialized synthetic biomaterials (Katja et al., 2016). Post-crosslinking methods (Ouyang et al., 2016) or hybrid printing methods (Fattahi et al., 2017) complement the current limitation of 3DBP methods. The limitation of animal models or PDX can be avoided by seeding primary breast cancer or metastatic cells in the 3D bio-printed breast cancer microenvironments to capture initial cues for proliferation and migration of breast cancer cells (Papapetrou, 2016). With 3DBP, these efforts can advance reliable *in vitro* modeling to attain better cancer therapeutics.

## Author contributions

JJ and EM conceived the overall topics of discussion. CK, JBB, JJ, and EM wrote the section of breast cancer microenvironments. JAB and JJ wrote the section of 3D bioprinting. All authors read and approved the final manuscript.

### Conflict of interest statement

The authors declare that the research was conducted in the absence of any commercial or financial relationships that could be construed as a potential conflict of interest.

## References

[B1] Acerbi I. Cassereau L. Dean I. Shi Q. Au A. Park C.. (2015). Human breast cancer invasion and aggression correlates with ECM stiffening and immune cell infiltration. Integr. Biol. (Camb). 7, 1120–1134. 10.1039/C5IB00040H 25959051 PMC4593730

[B2] Ahmadzadeh H. Webster M. R. Behera R. Jimenez Valencia A. M. Wirtz D. Weeraratna A. T.. (2017). Modeling the two-way feedback between contractility and matrix realignment reveals a nonlinear mode of cancer cell invasion. Proc. Natl. Acad. Sci. U.S.A. 114, E1617–E1626. 10.1073/pnas.1617037114 28196892 PMC5338523

[B3] Akthar S. Patel D. F. Beale R. C. Peiro T. Xu X. Gaggar A.. (2015). Matrikines are key regulators in modulating the amplitude of lung inflammation in acute pulmonary infection. Nat. Commun. 6:8423. 10.1038/ncomms9423 26400771 PMC4595997

[B4] Almstedt K. Sicking I. Battista M. J. Huangfu S. Heimes A. S. Weyer-Elberich V.. (2017). Prognostic significance of focal adhesion kinase in node-negative breast cancer. Breast Care (Basel). 12, 329–333. 10.1159/000477895 29234254 PMC5704693

[B5] Arnold K. M. Opdenaker L. M. Flynn D. Sims-Mourtada J. (2015). Wound healing and cancer stem cells: inflammation as a driver of treatment resistance in breast cancer. Cancer Growth Metastasis 8, 1–13. 10.4137/CGM.S11286 25674014 PMC4315129

[B6] Asghar W. El Assal R. Shafiee H. Pitteri S. Paulmurugan R. Demirci U. (2015). Engineering cancer microenvironments for *in vitro* 3-D tumor models. Mater. Today (Kidlington). 18, 539–553. 10.1016/j.mattod.2015.05.002 28458612 PMC5407188

[B7] Atkinson R. L. Yang W. T. Rosen D. G. Landis M. D. Wong H. Lewis M. T.. (2013). Cancer stem cell markers are enriched in normal tissue adjacent to triple negative breast cancer and inversely correlated with DNA repair deficiency. Breast Cancer Res. 15:R77. 10.1186/bcr3471 24008095 PMC4053576

[B8] Baba F. Swartz K. Van Buren R. Eickhoff J. Zhang Y. Wolberg W.. (2006). Syndecan-1 and syndecan-4 are overexpressed in an estrogen receptor-negative, highly proliferative breast carcinoma subtype. Breast Cancer Res. Treat. 98, 91–98. 10.1007/s10549-005-9135-2 16636895

[B9] Bae Y. K. Kim A. Kim M. K. Choi J. E. Kang S. H. Lee S. J. (2013). Fibronectin expression in carcinoma cells correlates with tumor aggressiveness and poor clinical outcome in patients with invasive breast cancer. Hum. Pathol. 44, 2028–2037. 10.1016/j.humpath.2013.03.006 23684510

[B10] Balanis N. Wendt M. K. Schiemann B. J. Wang Z. Schiemann W. P. Carlin C. R. (2013). Epithelial to mesenchymal transition promotes breast cancer progression via a fibronectin-dependent STAT3 signaling pathway. J. Biol. Chem. 288, 17954–17967. 10.1074/jbc.M113.475277 23653350 PMC3689941

[B11] Barcus C. E. Holt E. C. Keely P. J. Eliceiri K. W. Schuler L. A. (2015). Dense collagen-I matrices enhance pro-tumorigenic estrogen-prolactin crosstalk in MCF-7 and T47D breast cancer cells. PLoS ONE 10:e0116891. 10.1371/journal.pone.0116891 25607819 PMC4301649

[B12] Barcus C. E. O'leary K. A. Brockman J. L. Rugowski D. E. Liu Y. Garcia N. (2017). Elevated collagen-I augments tumor progressive signals, intravasation and metastasis of prolactin-induced estrogen receptor alpha positive mammary tumor cells. Breast Cancer Res. 19:9 10.1186/s13058-017-0801-1 28103936 PMC5244528

[B13] Beauvais D. M. Rapraeger A. C. (2003). Syndecan-1-mediated cell spreading requires signaling by αvβ3 integrins in human breast carcinoma cells. Exp. Cell Res. 286, 219–232. 10.1016/S0014-4827(03)00126-5 12749851

[B14] Ben-David U. Ha G. Tseng Y. Y. Greenwald N. F. Oh C. Shih J.. (2017). Patient-derived xenografts undergo mouse-specific tumor evolution. Nat. Genet. 49, 1567–1575. 10.1038/ng.3967 28991255 PMC5659952

[B15] Benton G. Arnaoutova I. George J. Kleinman H. K. Koblinski J. (2014). Matrigel: from discovery and ECM mimicry to assays and models for cancer research. Adv. Drug Deliv. Rev. 79–80, 3–18. 10.1016/j.addr.2014.06.005 24997339

[B16] Bielli A. Scioli M. G. Gentile P. Agostinelli S. Tarquini C. Cervelli V.. (2014). Adult adipose-derived stem cells and breast cancer: a controversial relationship. Springerplus 3:345. 10.1186/2193-1801-3-345 25089245 PMC4117859

[B17] Boere K. W. M. Blokzijl M. M. Visser J. Linssen J. E. A. Malda J. Hennink W. E. (2015). Biofabrication of reinforced 3D-scaffolds using two-component hydrogels. J. Mater. Chem. B 3, 9067–9078. 10.1039/C5TB01645B PMC7116180 32263038

[B18] Boland T. Xu T. Damon B. Cui X. (2006). Application of inkjet printing to tissue engineering. Biotechnol. J. 1, 910–917. 10.1002/biot.200600081 16941443

[B19] Borthwick L. A. Wynn T. A. Fisher A. J. (2013). Cytokine mediated tissue fibrosis. Biochim. Biophys. Acta 1832, 1049–1060. 10.1016/j.bbadis.2012.09.014 23046809 PMC3787896

[B20] Boyd N. F. Li Q. Melnichouk O. Huszti E. Martin L. J. Gunasekara A.. (2014). Evidence that breast tissue stiffness is associated with risk of breast cancer. PLoS ONE 9:e100937. 10.1371/journal.pone.0100937 25010427 PMC4091939

[B21] Branco da Cunha C. Klumpers D. D. Li W. A. Koshy S. T. Weaver J. C. Chaudhuri O.. (2014). Influence of the stiffness of three-dimensional alginate/collagen-I interpenetrating networks on fibroblast biology. Biomaterials 35, 8927–8936. 10.1016/j.biomaterials.2014.06.047 25047628

[B22] Breslin S. O'Driscoll L. (2016). The relevance of using 3D cell cultures, in addition to 2D monolayer cultures, when evaluating breast cancer drug sensitivity and resistance. Oncotarget 7, 45745–45756. 10.18632/oncotarget.9935 27304190 PMC5216757

[B23] Buchanan C. Stigliano I. Garay-Malpartida H. M. Rodrigues Gomes L. Puricelli L. Sogayar M. C.. (2010). Glypican-3 reexpression regulates apoptosis in murine adenocarcinoma mammary cells modulating PI3K/Akt and p38MAPK signaling pathways. Breast Cancer Res. Treat. 119, 559–574. 10.1007/s10549-009-0362-9 19288189

[B24] Burks H. E. Phamduy T. B. Azimi M. S. Saksena J. Burow M. E. Collins-Burow B. M.. (2016). Laser direct-write onto live tissues: a novel model for studying cancer cell migration. J. Cell. Physiol. 231, 2333–2338. 10.1002/jcp.25363 26923437 PMC4946993

[B25] Bussard K. M. Mutkus L. Stumpf K. Gomez-Manzano C. Marini F. C. (2016). Tumor-associated stromal cells as key contributors to the tumor microenvironment. Breast Cancer Res. 18:84 10.1186/s13058-016-0740-2 27515302 PMC4982339

[B26] Camnitz W. Burdick M. D. Strieter R. M. Mehrad B. Keeley E. C. (2012). Dose-dependent effect of statin therapy on circulating CXCL12 levels in patients with hyperlipidemia. Clin. Transl. Med. 1:23. 10.1186/2001-1326-1-23 23369699 PMC3560987

[B27] Carey S. P. Goldblatt Z. E. Martin K. E. Romero B. Williams R. M. Reinhart-King C. A. (2016). Local extracellular matrix alignment directs cellular protrusion dynamics and migration through Rac1 and FAK. Integr. Biol. (Camb). 8, 821–835. 10.1039/C6IB00030D 27384462 PMC4980151

[B28] Carey S. P. Martin K. E. Reinhart-King C. A. (2017). Three-dimensional collagen matrix induces a mechanosensitive invasive epithelial phenotype. Sci. Rep. 7:42088. 10.1038/srep42088 28186196 PMC5301232

[B29] Casbas-Hernandez P. Sun X. Roman-Perez E. D'arcy M. Sandhu R. Hishida A.. (2015). Tumor intrinsic subtype is reflected in cancer-adjacent tissue. Cancer Epidemiol. Biomarkers Prev. 24, 406–414. 10.1158/1055-9965.EPI-14-0934 25465802 PMC4437571

[B30] Cassidy J. W. Caldas C. Bruna A. (2015). Maintaining tumor heterogeneity in patient-derived tumor xenografts. Cancer Res. 75, 2963–2968. 10.1158/0008-5472.CAN-15-0727 26180079 PMC4539570

[B31] Castano Z. Marsh T. Tadipatri R. Kuznetsov H. S. Al-Shahrour F. Paktinat M.. (2013). Stromal EGF and igf-I together modulate plasticity of disseminated triple-negative breast tumors. Cancer Discov. 3, 922–935. 10.1158/2159-8290.CD-13-0041 23689072 PMC3740018

[B32] Cavallo-Medved D. Rudy D. Blum G. Bogyo M. Caglic D. Sloane B. F. (2009). Live-cell imaging demonstrates extracellular matrix degradation in association with active cathepsin B in caveolae of endothelial cells during tube formation. Exp. Cell Res. 315, 1234–1246. 10.1016/j.yexcr.2009.01.021 19331819 PMC2677760

[B33] Chang C. C. Boland E. D. Williams S. K. Hoying J. B. (2011). Direct-write bioprinting three-dimensional biohybrid systems for future regenerative therapies. J. Biomed. Mater. Res. B Appl. Biomater. 98, 160–170. 10.1002/jbm.b.31831 21504055 PMC3772543

[B34] Chaudhuri O. Gu L. Klumpers D. Darnell M. Bencherif S. A. Weaver J. C.. (2016). Hydrogels with tunable stress relaxation regulate stem cell fate and activity. Nat. Mater. 15, 326–334. 10.1038/nmat4489 26618884 PMC4767627

[B35] Chaudhuri O. Koshy S. T. Branco Da Cunha C. Shin J. W. Verbeke C. S. Allison K. H.. (2014). Extracellular matrix stiffness and composition jointly regulate the induction of malignant phenotypes in mammary epithelium. Nat. Mater. 13, 970–978. 10.1038/nmat4009 24930031

[B36] Chen M. B. Whisler J. A. Jeon J. S. Kamm R. D. (2013). Mechanisms of tumor cell extravasation in an *in vitro* microvascular network platform. Integr. Biol. (Camb). 5, 1262–1271. 10.1039/c3ib40149a 23995847 PMC4038741

[B37] Chimene D. Lennox K. K. Kaunas R. R. Gaharwar A. K. (2016). Advanced bioinks for 3D printing: a materials science perspective. Ann. Biomed. Eng. 44, 2090–2102. 10.1007/s10439-016-1638-y 27184494

[B38] Cohen D. L. Malone E. Lipson H. Bonassar L. J. (2006). Direct freeform fabrication of seeded hydrogels in arbitrary geometries. Tissue Eng. 12, 1325–1335. 10.1089/ten.2006.12.1325 16771645

[B39] Conklin M. W. Eickhoff J. C. Riching K. M. Pehlke C. A. Eliceiri K. W. Provenzano P. P.. (2011). Aligned collagen is a prognostic signature for survival in human breast carcinoma. Am. J. Pathol. 178, 1221–1232. 10.1016/j.ajpath.2010.11.076 21356373 PMC3070581

[B40] Cox T. R. Erler J. T. (2011). Remodeling and homeostasis of the extracellular matrix: implications for fibrotic diseases and cancer. Dis. Model. Mech. 4, 165–178. 10.1242/dmm.004077 21324931 PMC3046088

[B41] Creighton C. J. (2012). The molecular profile of luminal B breast cancer. Biologics 6, 289–297. 10.2147/BTT.S29923 22956860 PMC3430090

[B42] Cross V. L. Zheng Y. Won Choi N. Verbridge S. S. Sutermaster B. A. Bonassar L. J.. (2010). Dense type I collagen matrices that support cellular remodeling and microfabrication for studies of tumor angiogenesis and vasculogenesis *in vitro*. Biomaterials 31, 8596–8607. 10.1016/j.biomaterials.2010.07.072 20727585 PMC2949514

[B43] Dai X. Ma C. Lan Q. Xu T. (2016). 3D bioprinted glioma stem cells for brain tumor model and applications of drug susceptibility. Biofabrication 8:045005. 10.1088/1758-5090/8/4/045005 27725343

[B44] Dechaphunkul A. Phukaoloun M. Kanjanapradit K. Graham K. Ghosh S. Santos C. (2012). Prognostic significance of tissue inhibitor of metalloproteinase-1 in breast cancer. Int. J. Breast Cancer 2012:290854 10.1155/2012/290854 22988515 PMC3440855

[B45] De Palma M. Biziato D. Petrova T. V. (2017). Microenvironmental regulation of tumour angiogenesis. Nat. Rev. Cancer 17, 457–474. 10.1038/nrc.2017.51 28706266

[B46] Dunne L. W. Huang Z. Meng W. Fan X. Zhang N. Zhang Q.. (2014). Human decellularized adipose tissue scaffold as a model for breast cancer cell growth and drug treatments. Biomaterials 35, 4940–4949. 10.1016/j.biomaterials.2014.03.003 24661550

[B47] Egeblad M. Rasch M. G. Weaver V. M. (2010). Dynamic interplay between the collagen scaffold and tumor evolution. Curr. Opin. Cell Biol. 22, 697–706. 10.1016/j.ceb.2010.08.015 20822891 PMC2948601

[B48] Elderbroom J. L. Huang J. J. Gatza C. E. Chen J. How T. Starr M.. (2014). Ectodomain shedding of TbetaRIII is required for TbetaRIII-mediated suppression of TGF-beta signaling and breast cancer migration and invasion. Mol. Biol. Cell 25, 2320–2332. 10.1091/mbc.e13-09-0524 24966170 PMC4142606

[B49] Elsdale T. Bard J. (1972). Collagen substrata for studies on cell behavior. J. Cell Biol. 54, 626–637. 10.1083/jcb.54.3.626 4339818 PMC2200288

[B50] Fattahi P. Dover J. T. Brown J. L. (2017). 3D Near-field electrospinning of biomaterial microfibers with potential for blended microfiber-cell-loaded gel composite structures. Adv. Healthc. Mater. 6:1700456. 10.1002/adhm.201700456 28661043 PMC5638707

[B51] Fenner J. Stacer A. C. Winterroth F. Johnson T. D. Luker K. E. Luker G. D. (2014). Macroscopic stiffness of breast tumors predicts metastasis. Sci. Rep. 4:5512. 10.1038/srep05512 24981707 PMC4076689

[B52] Fernandez-Garcia B. Eiro N. Marin L. Gonzalez-Reyes S. Gonzalez L. O. Lamelas M. L.. (2014). Expression and prognostic significance of fibronectin and matrix metalloproteases in breast cancer metastasis. Histopathology 64, 512–522. 10.1111/his.12300 24117661

[B53] Florczyk S. J. Liu G. Kievit F. M. Lewis A. M. Wu J. D. Zhang M. (2012). 3D porous chitosan-alginate scaffolds: a new matrix for studying prostate cancer cell-lymphocyte interactions *in vitro*. Adv. Healthc. Mater. 1, 590–599. 10.1002/adhm.201100054 23184794 PMC3682216

[B54] Florczyk S. J. Wang K. Jana S. Wood D. L. Sytsma S. K. Sham J.. (2013). Porous chitosan-hyaluronic acid scaffolds as a mimic of glioblastoma microenvironment ECM. Biomaterials 34, 10143–10150. 10.1016/j.biomaterials.2013.09.034 24075410 PMC3843957

[B55] Fong E. L. Lamhamedi-Cherradi S. E. Burdett E. Ramamoorthy V. Lazar A. J. Kasper F. K.. (2013). Modeling Ewing sarcoma tumors *in vitro* with 3D scaffolds. Proc. Natl. Acad. Sci. U.S.A. 110, 6500–6505. 10.1073/pnas.1221403110 23576741 PMC3631678

[B56] Fong E. L. Santoro M. Farach-Carson M. C. Kasper F. K. Mikos A. G. (2014). Tissue engineering perfusable cancer models. Curr. Opin. Chem. Eng. 3, 112–117. 10.1016/j.coche.2013.12.008 24634812 PMC3949682

[B57] Fullar A. Dudas J. Olah L. Hollosi P. Papp Z. Sobel G. (2015). Remodeling of extracellular matrix by normal and tumor-associated fibroblasts promotes cervical cancer progression. BMC Cancer 15:256 10.1186/s12885-015-1272-3 25885552 PMC4409756

[B58] Gajewski T. F. Schreiber H. Fu Y. X. (2013). Innate and adaptive immune cells in the tumor microenvironment. Nat. Immunol. 14, 1014–1022. 10.1038/ni.2703 24048123 PMC4118725

[B59] Gangadhara S. Smith C. Barrett-Lee P. Hiscox S. (2016). 3D culture of Her2+ breast cancer cells promotes AKT to MAPK switching and a loss of therapeutic response. BMC Cancer 16:345 10.1186/s12885-016-2377-z 27251376 PMC4888214

[B60] Gill B. J. Gibbons D. L. Roudsari L. C. Saik J. E. Rizvi Z. H. Roybal J. D.. (2012). A synthetic matrix with independently tunable biochemistry and mechanical properties to study epithelial morphogenesis and EMT in a lung adenocarcinoma model. Cancer Res. 72, 6013–6023. 10.1158/0008-5472.CAN-12-0895 22952217 PMC3632398

[B61] Gill B. J. West J. L. (2014). Modeling the tumor extracellular matrix: tissue engineering tools repurposed towards new frontiers in cancer biology. J. Biomech. 47, 1969–1978. 10.1016/j.jbiomech.2013.09.029 24300038

[B62] Gocheva V. Naba A. Bhutkar A. Guardia T. Miller K. M. Li C. M.. (2017). Quantitative proteomics identify Tenascin-C as a promoter of lung cancer progression and contributor to a signature prognostic of patient survival. Proc. Natl. Acad. Sci. U.S.A. 114, E5625–E5634. 10.1073/pnas.1707054114 28652369 PMC5514763

[B63] Gu L. Mooney D. J. (2016). Biomaterials and emerging anticancer therapeutics: engineering the microenvironment. Nat. Rev. Cancer 16, 56–66. 10.1038/nrc.2015.3 26694936 PMC4790726

[B64] Guillemot F. Guillotin B. Catros S. Souquet A. Mezel C. Keriquel V. (2010). High-throughput biological laser printing: droplet ejection mechanism, integration of a dedicated workstation, and bioprinting of cells and biomaterials, in Cell and Organ Printing, eds Ringeisen B. R. Spargo B. J. Wu P. K. (Dordrecht: Springer Netherlands), 95–113.

[B65] Guiro K. Patel S. A. Greco S. J. Rameshwar P. Arinzeh T. L. (2015). Investigating breast cancer cell behavior using tissue engineering scaffolds. PLoS ONE 10:e0118724. 10.1371/journal.pone.0118724 25837691 PMC4383476

[B66] Guller A. E. Grebenyuk P. N. Shekhter A. B. Zvyagin A. V. Deyev S. M. (2016). Bioreactor-based tumor tissue engineering. Acta Nat. 8, 44–58. 27795843 PMC5081698

[B67] Gurski L. A. Petrelli N. J. Jia X. Farach-Carson M. C. (2017). 3D matrices for anti-cancer drug testing and development. Oncol. Issues 25, 20–25. 10.1080/10463356.2010.11883480

[B68] Guzman A. Sanchez Alemany V. Nguyen Y. Zhang C. R. Kaufman L. J. (2017). A novel 3D *in vitro* metastasis model elucidates differential invasive strategies during and after breaching basement membrane. Biomaterials 115, 19–29. 10.1016/j.biomaterials.2016.11.014 27880891

[B69] Haabeth O. A. Tveita A. A. Fauskanger M. Schjesvold F. Lorvik K. B. Hofgaard P. O. (2014). How do CD4^+^ T cells detect and eliminate tumor cells that either lack or express MHC Class II molecules? Front. Immunol. 5:174 10.3389/fimmu.2014.00174 24782871 PMC3995058

[B70] Haage A. Schneider I. C. (2014). Cellular contractility and extracellular matrix stiffness regulate matrix metalloproteinase activity in pancreatic cancer cells. FASEB J. 28, 3589–3599. 10.1096/fj.13-245613 24784579

[B71] Haeger A. Krause M. Wolf K. Friedl P. (2014). Cell jamming: collective invasion of mesenchymal tumor cells imposed by tissue confinement. Biochim. Biophys. Acta 1840, 2386–2395. 10.1016/j.bbagen.2014.03.020 24721714

[B72] Hanker A. B. Estrada M. V. Bianchini G. Moore P. D. Zhao J. Cheng F.. (2017). Extracellular matrix/integrin signaling promotes resistance to combined inhibition of HER2 and PI3K in HER2^+^ breast cancer. Cancer Res. 77, 3280–3292. 10.1158/0008-5472.CAN-16-2808 28396358 PMC5482178

[B73] Hayashi M. Yamamoto Y. Ibusuki M. Fujiwara S. Yamamoto S. Tomita S.. (2012). Evaluation of tumor stiffness by elastography is predictive for pathologic complete response to neoadjuvant chemotherapy in patients with breast cancer. Ann. Surg. Oncol. 19, 3042–3049. 10.1245/s10434-012-2343-1 22476757

[B74] Hayes M. J. Thomas D. Emmons A. Giordano T. J. Kleer C. G. (2008). Genetic changes of Wnt pathway genes are common events in metaplastic carcinomas of the breast. Clin. Cancer Res. 14, 4038–4044. 10.1158/1078-0432.CCR-07-4379 18593979 PMC3060761

[B75] Hielscher A. C. Qiu C. Gerecht S. (2012). Breast cancer cell-derived matrix supports vascular morphogenesis. Am. J. Physiol. Cell Physiol. 302, C1243–C1256. 10.1152/ajpcell.00011.2012 22277754 PMC3774551

[B76] Hinderer S. Layland S. L. Schenke-Layland K. (2016). ECM and ECM-like materials-biomaterials for applications in regenerative medicine and cancer therapy. Adv. Drug Deliv. Rev. 97, 260–269. 10.1016/j.addr.2015.11.019 26658243

[B77] Hinderer S. Schesny M. Bayrak A. Ibold B. Hampel M. Walles T.. (2012). Engineering of fibrillar decorin matrices for a tissue-engineered trachea. Biomaterials 33, 5259–5266. 10.1016/j.biomaterials.2012.03.075 22521489

[B78] Hinton T. J. Jallerat Q. Palchesko R. N. Park J. H. Grodzicki M. S. Shue H. J.. (2015). Three-dimensional printing of complex biological structures by freeform reversible embedding of suspended hydrogels. Sci. Adv. 1:e1500758. 10.1126/sciadv.1500758 26601312 PMC4646826

[B79] Hirt C. Papadimitropoulos A. Mele V. Muraro M. G. Mengus C. Iezzi G. (2014). “*In vitro*” 3D models of tumor-immune system interaction. Adv. Drug Deliv. Rev. 79–80, 145–154. 10.1016/j.addr.2014.05.003 24819215

[B80] Hirt C. Papadimitropoulos A. Muraro M. G. Mele V. Panopoulos E. Cremonesi E.. (2015). Bioreactor-engineered cancer tissue-like structures mimic phenotypes, gene expression profiles and drug resistance patterns observed “*in vivo*.” Biomaterials 62, 138–146. 10.1016/j.biomaterials.2015.05.037 26051518

[B81] Hoy A. J. Balaban S. Saunders D. N. (2017). Adipocyte-tumor cell metabolic crosstalk in breast cancer. Trends Mol. Med. 23, 381–392. 10.1016/j.molmed.2017.02.009 28330687

[B82] Hoye A. M. Erler J. T. (2016). Structural ECM components in the premetastatic and metastatic niche. Am. J. Physiol. Cell Physiol. 310, C955–C967. 10.1152/ajpcell.00326.2015 27053524

[B83] Hribar K. C. Finlay D. Ma X. Qu X. Ondeck M. G. Chung P. H.. (2015). Nonlinear 3D projection printing of concave hydrogel microstructures for long-term multicellular spheroid and embryoid body culture. Lab. Chip. 15, 2412–2418. 10.1039/C5LC00159E 25900329 PMC4439309

[B84] Huang T. Q. Qu X. Liu J. Chen S. (2014). 3D printing of biomimetic microstructures for cancer cell migration. Biomed. Microdevices 16, 127–132. 10.1007/s10544-013-9812-6 24150602 PMC3945947

[B85] Imamura Y. Mukohara T. Shimono Y. Funakoshi Y. Chayahara N. Toyoda M.. (2015). Comparison of 2D- and 3D-culture models as drug-testing platforms in breast cancer. Oncol. Rep. 33, 1837–1843. 10.3892/or.2015.3767 25634491

[B86] Infanger D. W. Lynch M. E. Fischbach C. (2013). Engineered culture models for studies of tumor-microenvironment interactions. Annu. Rev. Biomed. Eng. 15, 29–53. 10.1146/annurev-bioeng-071811-150028 23642249 PMC11332991

[B87] Insua-Rodriguez J. Oskarsson T. (2016). The extracellular matrix in breast cancer. Adv. Drug Deliv. Rev. 97, 41–55. 10.1016/j.addr.2015.12.017 26743193

[B88] Jackson H. W. Defamie V. Waterhouse P. Khokha R. (2017). TIMPs: versatile extracellular regulators in cancer. Nat. Rev. Cancer 17, 38–53. 10.1038/nrc.2016.115 27932800

[B89] Jadalannagari S. Converse G. Mcfall C. Buse E. Filla M. Villar M. T.. (2017). Decellularized Wharton's Jelly from human umbilical cord as a novel 3D scaffolding material for tissue engineering applications. PLoS ONE 12:e0172098. 10.1371/journal.pone.0172098 28222169 PMC5319682

[B90] Jaganathan H. Gage J. Leonard F. Srinivasan S. Souza G. R. Dave B.. (2014). Three-dimensional *in vitro* co-culture model of breast tumor using magnetic levitation. Sci. Rep. 4:6468. 10.1038/srep06468 25270048 PMC4180823

[B91] Jain A. Betancur M. Patel G. D. Valmikinathan C. M. Mukhatyar V. J. Vakharia A.. (2014). Guiding intracortical brain tumour cells to an extracortical cytotoxic hydrogel using aligned polymeric nanofibres. Nat. Mater. 13, 308–316. 10.1038/nmat3878 24531400

[B92] Jeon J. S. Bersini S. Gilardi M. Dubini G. Charest J. L. Moretti M.. (2015). Human 3D vascularized organotypic microfluidic assays to study breast cancer cell extravasation. Proc. Natl. Acad. Sci. U.S.A. 112, 214–219. 10.1073/pnas.1417115112 25524628 PMC4291627

[B93] Jeong S. Y. Lee J. H. Shin Y. Chung S. Kuh H. J. (2016). Co-culture of tumor spheroids and fibroblasts in a collagen matrix-incorporated microfluidic chip mimics reciprocal activation in solid tumor microenvironment. PLoS ONE 11:e0159013. 10.1371/journal.pone.0159013 27391808 PMC4938568

[B94] Jovanovic B. Beeler J. S. Pickup M. W. Chytil A. Gorska A. E. Ashby W. J.. (2014). Transforming growth factor beta receptor type III is a tumor promoter in mesenchymal-stem like triple negative breast cancer. Breast Cancer Res. 16:R69. 10.1186/bcr3684 24985072 PMC4095685

[B95] Karamanou K. Franchi M. Piperigkou Z. Perreau C. Maquart F. X. Vynios D. H.. (2017). Lumican effectively regulates the estrogen receptors-associated functional properties of breast cancer cells, expression of matrix effectors and epithelial-to-mesenchymal transition. Sci. Rep. 7:45138. 10.1038/srep45138 28332606 PMC5362815

[B96] Karayiannakis A. J. Nakopoulou L. Gakiopoulou H. Keramopoulos A. Davaris P. S. Pignatelli M. (2001). Expression patterns of beta-catenin in in situ and invasive breast cancer. Eur. J. Surg. Oncol. 27, 31–36. 10.1053/ejso.1999.1017 11237489

[B97] Karousou E. D'angelo M. L. Kouvidi K. Vigetti D. Viola M. Nikitovic D. (2014). Collagen VI and hyaluronan: the common role in breast cancer. Biomed Res. Int. 2014:606458 10.1155/2014/606458 25126569 PMC4121998

[B98] Katja H. Shengmao L. Liesbeth T. Sandra Van V. Linxia G. Aleksandr O. (2016). Bioink properties before, during and after 3D bioprinting. Biofabrication 8:032002 10.1088/1758-5090/8/3/032002 27658612

[B99] Katt M. E. Placone A. L. Wong A. D. Xu Z. S. Searson P. C. (2016). *In vitro* tumor models: advantages, disadvantages, variables, and selecting the right platform. Front. Bioeng. Biotechnol. 4:12. 10.3389/fbioe.2016.00012 26904541 PMC4751256

[B100] Knowlton S. Onal S. Yu C. H. Zhao J. J. Tasoglu S. (2015). Bioprinting for cancer research. Trends Biotechnol. 33, 504–513. 10.1016/j.tibtech.2015.06.007 26216543

[B101] Kolesky D. B. Truby R. L. Gladman A. S. Busbee T. A. Homan K. A. Lewis J. A. (2014). 3D bioprinting of vascularized, heterogeneous cell-laden tissue constructs. Adv. Mater. Weinheim. 26, 3124–3130. 10.1002/adma.201305506 24550124

[B102] Kulkarni V. Bodas D. Paknikar K. (2018). Assessment of an integrative anticancer treatment using an *in vitro* perfusion-enabled 3D breast tumor model. ACS Biomater. Sci. Eng. 4, 1407–1417. 10.1021/acsbiomaterials.8b00153 33418670

[B103] Kwon S. Y. Chae S. W. Wilczynski S. P. Arain A. Carpenter Philip M. (2012). Laminin 332 expression in breast carcinoma. Appl. Immunohistochem. Mol. Morphol. 20, 159–164. 10.1097/PAI.0b013e3182329e8f 22427740 PMC3302204

[B104] Laronda M. M. Rutz A. L. Xiao S. Whelan K. A. Duncan F. E. Roth E. W.. (2017). A bioprosthetic ovary created using 3D printed microporous scaffolds restores ovarian function in sterilized mice. Nat. Commun. 8:15261. 10.1038/ncomms15261 28509899 PMC5440811

[B105] Lazaro G. Smith C. Goddard L. Jordan N. Mcclelland R. Barrett-Lee P.. (2013). Targeting focal adhesion kinase in ER+/HER2+ breast cancer improves trastuzumab response. Endocr. Relat. Cancer 20, 691–704. 10.1530/ERC-13-0019 23900794

[B106] Lee J. Y. Chaudhuri O. (2017). Regulation of breast cancer progression by extracellular matrix mechanics: insights from 3D culture models. ACS Biomater. Sci. Eng. 4, 302–313. 10.1021/acsbiomaterials.7b00071 33418725

[B107] Leggett S. E. Khoo A. S. Wong I. Y. (2017). Multicellular tumor invasion and plasticity in biomimetic materials. Biomater. Sci. 5, 1460–1479. 10.1039/C7BM00272F 28530743 PMC5531215

[B108] Leonard F. Godin B. (2016). 3D *In vitro* model for breast cancer research using magnetic levitation and bioprinting method. Methods Mol. Biol. 1406, 239–251. 10.1007/978-1-4939-3444-7_21 26820961 PMC4961210

[B109] Levental K. R. Yu H. Kass L. Lakins J. N. Egeblad M. Erler J. T.. (2009). Matrix crosslinking forces tumor progression by enhancing integrin signaling. Cell 139, 891–906. 10.1016/j.cell.2009.10.027 19931152 PMC2788004

[B110] Lewis D. M. Tang V. Jain N. Isser A. Xia Z. Gerecht S. (2017). Collagen fiber architecture regulates hypoxic sarcoma cell migration. ACS Biomater. Sci. Eng. 4, 400–409. 10.1021/acsbiomaterials.7b00056 33418732

[B111] Lin S. Y. Xia W. Wang J. C. Kwong K. Y. Spohn B. Wen Y.. (2000). Beta-catenin, a novel prognostic marker for breast cancer: its roles in cyclin D1 expression and cancer progression. Proc. Natl. Acad. Sci. U.S.A. 97, 4262–4266. 10.1073/pnas.060025397 10759547 PMC18221

[B112] Ling K. Huang G. Y. Liu J. C. Zhang X. H. Ma Y. F. Lu T. J. (2015). Bioprinting-based high-throughput fabrication of three-dimensional MCF-7 human breast cancer cellular spheroids. Engineering 1, 269–274. 10.15302/J-ENG-2015062

[B113] Liu J. Tan Y. Zhang H. Zhang Y. Xu P. Chen J.. (2012). Soft fibrin gels promote selection and growth of tumorigenic cells. Nat. Mater. 11, 734–741. 10.1038/nmat3361 22751180 PMC3405191

[B114] Liu T. Wang X. Karsdal M. A. Leeming D. J. Genovese F. (2012). Molecular serum markers of liver fibrosis. Biomark. Insights 7, 105–117. 10.4137/BMI.S10009 22872786 PMC3412619

[B115] Lo C. M. Wang H. B. Dembo M. Wang Y. L. (2000). Cell movement is guided by the rigidity of the substrate. Biophys. J. 79, 144–152. 10.1016/S0006-3495(00)76279-5 10866943 PMC1300921

[B116] Loozen L. D. Wegman F. Oner F. C. Dhert W. J. A. Alblas J. (2013). Porous bioprinted constructs in BMP-2 non-viral gene therapy for bone tissue engineering. J. Mater. Chem. B 1, 6619–6626. 10.1039/c3tb21093f 32261270

[B117] Loussouarn D. Campion L. Sagan C. Frenel J. S. Dravet F. Classe J. M.. (2008). Prognostic impact of syndecan-1 expression in invasive ductal breast carcinomas. Br. J. Cancer 98, 1993–1998. 10.1038/sj.bjc.6604400 18542065 PMC2441962

[B118] Lu W. D. Zhang L. Wu C. L. Liu Z. G. Lei G. Y. Liu J.. (2014). Development of an acellular tumor extracellular matrix as a three-dimensional scaffold for tumor engineering. PLoS ONE 9:e103672. 10.1371/journal.pone.0103672 25072252 PMC4114977

[B119] Ma L. Barker J. Zhou C. Li W. Zhang J. Lin B.. (2012). Towards personalized medicine with a three-dimensional micro-scale perfusion-based two-chamber tissue model system. Biomaterials 33, 4353–4361. 10.1016/j.biomaterials.2012.02.054 22429982 PMC3569495

[B120] Maeda T. Alexander C. M. Friedl A. (2004). Induction of syndecan-1 expression in stromal fibroblasts promotes proliferation of human breast cancer cells. Cancer Res. 64, 612–621. 10.1158/0008-5472.CAN-03-2439 14744776

[B121] Magdeldin T. Lopez-Davila V. Pape J. Cameron G. W. Emberton M. Loizidou M.. (2017). Engineering a vascularised 3D *in vitro* model of cancer progression. Sci. Rep. 7:44045. 10.1038/srep44045 28276469 PMC5343474

[B122] Majety M. Pradel L. P. Gies M. Ries C. H. (2015). Fibroblasts influence survival and therapeutic response in a 3D co-culture model. PLoS ONE 10:e0127948. 10.1371/journal.pone.0127948 26053043 PMC4460080

[B123] Malda J. Visser J. Melchels F. P. Jungst T. Hennink W. E. Dhert W. J.. (2013). 25th anniversary article: engineering hydrogels for biofabrication. Adv. Mater. Weinheim. 25, 5011–5028. 10.1002/adma.201302042 24038336

[B124] Mao Y. Keller E. T. Garfield D. H. Shen K. Wang J. (2013). Stromal cells in tumor microenvironment and breast cancer. Cancer Metastasis Rev. 32, 303–315. 10.1007/s10555-012-9415-3 23114846 PMC4432936

[B125] Martinez-Outschoorn U. E. Goldberg A. Lin Z. Ko Y. H. Flomenberg N. Wang C.. (2011). Anti-estrogen resistance in breast cancer is induced by the tumor microenvironment and can be overcome by inhibiting mitochondrial function in epithelial cancer cells. Cancer Biol. Ther. 12, 924–938. 10.4161/cbt.12.10.17780 22041887 PMC3280908

[B126] Maskarinec G. Woolcott C. G. Kolonel L. N. (2010). Mammographic density as a predictor of breast cancer outcome. Future Oncol. 6, 351–354. 10.2217/fon.10.3 20222792 PMC2928849

[B127] Mason B. N. Starchenko A. Williams R. M. Bonassar L. J. Reinhart-King C. A. (2013). Tuning three-dimensional collagen matrix stiffness independently of collagen concentration modulates endothelial cell behavior. Acta Biomater. 9, 4635–4644. 10.1016/j.actbio.2012.08.007 22902816 PMC3508162

[B128] McGrail D. J. Khambhati N. N. Qi M. X. Patel K. S. Ravikumar N. Brandenburg C. P.. (2015). Alterations in ovarian cancer cell adhesion drive taxol resistance by increasing microtubule dynamics in a FAK-dependent manner. Sci. Rep. 5:9529. 10.1038/srep09529 25886093 PMC4400875

[B129] McGrail D. J. Kieu Q. M. Dawson M. R. (2014). The malignancy of metastatic ovarian cancer cells is increased on soft matrices through a mechanosensitive Rho-ROCK pathway. J. Cell Sci. 127, 2621–2626. 10.1242/jcs.144378 24741068 PMC4058108

[B130] McKinnon D. D. Domaille D. W. Cha J. N. Anseth K. S. (2014). Biophysically defined and cytocompatible covalently adaptable networks as viscoelastic 3D cell culture systems. Adv. Mater. Weinheim. 26, 865–872. 10.1002/adma.201303680 24127293 PMC4582033

[B131] Mishra D. K. Creighton C. J. Zhang Y. Chen F. Thrall M. J. Kim M. P. (2015). *Ex vivo* four-dimensional lung cancer model mimics metastasis. Ann. Thorac. Surg. 99, 1149–1156. 10.1016/j.athoracsur.2014.08.085 25701100 PMC4387006

[B132] Mora-Solano C. Collier J. H. (2014). Engaging adaptive immunity with biomaterials. J Mater Chem B 2, 2409–2421. 10.1039/C3TB21549K 24729870 PMC3979482

[B133] Morgan M. M. Johnson B. P. Livingston M. K. Schuler L. A. Alarid E. T. Sung K. E.. (2016). Personalized *in vitro* cancer models to predict therapeutic response: challenges and a framework for improvement. Pharmacol. Ther. 165, 79–92. 10.1016/j.pharmthera.2016.05.007 27218886 PMC5439438

[B134] Morris B. A. Burkel B. Ponik S. M. Fan J. Condeelis J. S. Aguirre-Ghiso J. A.. (2016). Collagen matrix density drives the metabolic shift in breast cancer cells. EBioMedicine 13, 146–156. 10.1016/j.ebiom.2016.10.012 27743905 PMC5264313

[B135] Motz G. T. Coukos G. (2011). The parallel lives of angiogenesis and immunosuppression: cancer and other tales. Nat. Rev. Immunol. 11, 702–711. 10.1038/nri3064 21941296

[B136] Mouw J. K. Ou G. Weaver V. M. (2014). Extracellular matrix assembly: a multiscale deconstruction. Nat. Rev. Mol. Cell Biol. 15, 771–785. 10.1038/nrm3902 25370693 PMC4682873

[B137] Muhamed J. Rajan A. Surendran A. Jaleel A. Anilkumar T. V. (2017). Comparative profiling of extractable proteins in extracellular matrices of porcine cholecyst and jejunum intended for preparation of tissue engineering scaffolds. J. Biomed. Mater. Res. B Appl. Biomater. 105, 489–496. 10.1002/jbm.b.33567 26546090

[B138] Murphy S. V. Atala A. (2014). 3D bioprinting of tissues and organs. Nat. Biotechnol. 32, 773–785. 10.1038/nbt.2958 25093879

[B139] Naba A. Clauser K. R. Hoersch S. Liu H. Carr S. A. Hynes R. O. (2012). The matrisome: in silico definition and *in vivo* characterization by proteomics of normal and tumor extracellular matrices. Mol. Cell. Proteomics 11:M111.014647. 10.1074/mcp.M111.014647 22159717 PMC3322572

[B140] Naba A. Clauser K. R. Whittaker C. A. Carr S. A. Tanabe K. K. Hynes R. O. (2014). Extracellular matrix signatures of human primary metastatic colon cancers and their metastases to liver. BMC Cancer 14:518 10.1186/1471-2407-14-518 25037231 PMC4223627

[B141] Nguyen-Ngoc K. V. Cheung K. J. Brenot A. Shamir E. R. Gray R. S. Hines W. C.. (2012). ECM microenvironment regulates collective migration and local dissemination in normal and malignant mammary epithelium. Proc. Natl. Acad. Sci. U.S.A. 109, E2595–E2604. 10.1073/pnas.1212834109 22923691 PMC3465416

[B142] Odde D. J. Renn M. J. (1999). Laser-guided direct writing for applications in biotechnology. Trends Biotechnol. 17, 385–389. 10.1016/S0167-7799(99)01355-4 10481169

[B143] Odde D. J. Renn M. J. (2000). Laser-guided direct writing of living cells. Biotechnol. Bioeng. 67, 312–318. 10.1002/(SICI)1097-0290(20000205)67:3<312::AID-BIT7>3.0.CO;2-F 10620261

[B144] Ouyang L. L. Highley C. B. Rodell C. B. Sun W. Burdick J. A. (2016). 3D Printing of shear-thinning hyaluronic acid hydrogels with secondary cross-linking. ACS Biomater. Sci. Eng. 2, 1743–1751. 10.1021/acsbiomaterials.6b00158 33440472

[B145] Ozaki S. Ikeda S. Ishizaki Y. Kurihara T. Tokumoto N. Iseki M.. (2005). Alterations and correlations of the components in the Wnt signaling pathway and its target genes in breast cancer. Oncol. Rep. 14, 1437–1443. 10.3892/or.14.6.1437 16273236

[B146] Ozbolat I. T. Hospodiuk M. (2016). Current advances and future perspectives in extrusion-based bioprinting. Biomaterials 76, 321–343. 10.1016/j.biomaterials.2015.10.076 26561931

[B147] Ozbolat I. T. Moncal K. K. Gudapati H. (2017). Evaluation of bioprinter technologies. Addit. Manuf. 13, 179–200. 10.1016/j.addma.2016.10.003

[B148] Papapetrou E. P. (2016). Patient-derived induced pluripotent stem cells in cancer research and precision oncology. Nat. Med. 22, 1392–1401. 10.1038/nm.4238 27923030 PMC5233709

[B149] Paulsen S. J. Miller J. S. (2015). Tissue vascularization through 3D printing: will technology bring us flow? Dev. Dyn. 244, 629–640. 10.1002/dvdy.24254 25613150

[B150] Pence K. A. Mishra D. K. Thrall M. Dave B. Kim M. P. (2017). Breast cancer cells form primary tumors on *ex vivo* four-dimensional lung model. J. Surg. Res. 210, 181–187. 10.1016/j.jss.2016.11.019 28457326

[B151] Peng W. Unutmaz D. Ozbolat I. T. (2016). Bioprinting towards Physiologically Relevant Tissue Models for Pharmaceutics. Trends Biotechnol. 34, 722–732. 10.1016/j.tibtech.2016.05.013 27296078

[B152] Peters M. G. Farias E. Colombo L. Filmus J. Puricelli L. Bal De Kier Joffe E. (2003). Inhibition of invasion and metastasis by glypican-3 in a syngeneic breast cancer model. Breast Cancer Res. Treat. 80, 221–232. 10.1023/A:1024549729256 12908826

[B153] Phamduy T. B. Sweat R. S. Azimi M. S. Burow M. E. Murfee W. L. Chrisey D. B. (2015). Printing cancer cells into intact microvascular networks: a model for investigating cancer cell dynamics during angiogenesis. Integr. Biol. (Camb). 7, 1068–1078. 10.1039/C5IB00151J 26190039 PMC4785810

[B154] Pinto M. L. Rios E. Silva A. C. Neves S. C. Caires H. R. Pinto A. T.. (2017). Decellularized human colorectal cancer matrices polarize macrophages towards an anti-inflammatory phenotype promoting cancer cell invasion via CCL18. Biomaterials 124, 211–224. 10.1016/j.biomaterials.2017.02.004 28209528

[B155] Plodinec M. Loparic M. Monnier C. A. Obermann E. C. Zanetti-Dallenbach R. Oertle P.. (2012). The nanomechanical signature of breast cancer. Nat. Nanotechnol. 7, 757–765. 10.1038/nnano.2012.167 23085644

[B156] Pompili L. Porru M. Caruso C. Biroccio A. Leonetti C. (2016). Patient-derived xenografts: a relevant preclinical model for drug development. J. Exp. Clin. Cancer Res. 35:189 10.1186/s13046-016-0462-4 27919280 PMC5139018

[B157] Powell D. R. Huttenlocher A. (2016). Neutrophils in the tumor microenvironment. Trends Immunol. 37, 41–52. 10.1016/j.it.2015.11.008 26700397 PMC4707100

[B158] Pradhan S. Hassani I. Clary J. M. Lipke E. A. (2016). Polymeric biomaterials for *in vitro* cancer tissue engineering and drug testing applications. Tissue Eng. B Rev. 22, 470–484. 10.1089/ten.teb.2015.0567 27302080

[B159] Pradhan S. Hassani I. Seeto W. J. Lipke E. A. (2017). PEG-fibrinogen hydrogels for three-dimensional breast cancer cell culture. J. Biomed. Mater. Res. A 105, 236–252. 10.1002/jbm.a.35899 27615742

[B160] Prasad C. P. Mirza S. Sharma G. Prashad R. Dattagupta S. Rath G.. (2008). Epigenetic alterations of CDH1 and APC genes: relationship with activation of Wnt/beta-catenin pathway in invasive ductal carcinoma of breast. Life Sci. 83, 318–325. 10.1016/j.lfs.2008.06.019 18662704

[B161] Provenzano P. P. Inman D. R. Eliceiri K. W. Knittel J. G. Yan L. Rueden C. T.. (2008). Collagen density promotes mammary tumor initiation and progression. BMC Med. 6:11. 10.1186/1741-7015-6-11 18442412 PMC2386807

[B162] Provenzano P. P. Inman D. R. Eliceiri K. W. Keely P. J. (2009). Matrix density-induced mechanoregulation of breast cell phenotype, signaling and gene expression through a FAK-ERK linkage. Oncogene 28, 4326–4343. 10.1038/onc.2009.299 19826415 PMC2795025

[B163] Qazi T. H. Mooney D. J. Duda G. N. Geissler S. (2017). Biomaterials that promote cell-cell interactions enhance the paracrine function of MSCs. Biomaterials 140, 103–114. 10.1016/j.biomaterials.2017.06.019 28644976

[B164] Qian P. Zuo Z. Wu Z. Meng X. Li G. Wu Z.. (2011). Pivotal role of reduced let-7g expression in breast cancer invasion and metastasis. Cancer Res. 71, 6463–6474. 10.1158/0008-5472.CAN-11-1322 21868760

[B165] Raab-Westphal S. Marshall J. F. Goodman S. L. (2017). Integrins as therapeutic targets: successes and cancers. Cancers (Basel) 9:110. 10.3390/cancers9090110 28832494 PMC5615325

[B166] Raimondi C. Gradilone A. Naso G. Vincenzi B. Petracca A. Nicolazzo C.. (2011). Epithelial-mesenchymal transition and stemness features in circulating tumor cells from breast cancer patients. Breast Cancer Res. Treat. 130, 449–455. 10.1007/s10549-011-1373-x 21298334

[B167] Regier M. C. Alarid E. T. Beebe D. J. (2016). Progress towards understanding heterotypic interactions in multi-culture models of breast cancer. Integr. Biol. (Camb). 8, 684–692. 10.1039/C6IB00001K 27097801 PMC4993016

[B168] Rijal G. Li W. (2017). A versatile 3D tissue matrix scaffold system for tumor modeling and drug screening. Sci. Adv. 3:e1700764. 10.1126/sciadv.1700764 28924608 PMC5597314

[B169] Ringeisen B. R. Kim H. Barron J. A. Krizman D. B. Chrisey D. B. Jackman S.. (2004). Laser printing of pluripotent embryonal carcinoma cells. Tissue Eng. 10, 483–491. 10.1089/107632704323061843 15165465

[B170] Ringeisen B. R. Wu P. K. Kim H. Pique A. Auyeung R. Y. Young H. D.. (2002). Picoliter-scale protein microarrays by laser direct write. Biotechnol. Prog. 18, 1126–1129. 10.1021/bp015516g 12363367

[B171] Rizvi I. Celli J. P. Xu F. Evans C. L. Abu-Yousif A. O. Muzikansky A. (2011). Biologically relevant 3D tumor arrays: treatment response and the importance of stromal partners, in Proc. SPIE 7886, Optical Methods for Tumor Treatment and Detection: Mechanisms and Techniques in Photodynamic Therapy XX, 788609 (San Francisco, CA).

[B172] Roudsari L. C. West J. L. (2016). Studying the influence of angiogenesis in *in vitro* cancer model systems. Adv. Drug Deliv. Rev. 97, 250–259. 10.1016/j.addr.2015.11.004 26571106

[B173] Rozario T. DeSimone D. W. (2010). The extracellular matrix in development and morphogenesis: a dynamic view. Dev. Biol. 341, 126–140. 10.1016/j.ydbio.2009.10.026 19854168 PMC2854274

[B174] Rutz A. L. Hyland K. E. Jakus A. E. Burghardt W. R. Shah R. N. (2015). A multimaterial bioink method for 3D printing tunable, cell-compatible hydrogels. Adv. Mater. Weinheim. 27, 1607–1614. 10.1002/adma.201405076 25641220 PMC4476973

[B175] Ryo A. Nakamura M. Wulf G. Liou Y. C. Lu K. P. (2001). Pin1 regulates turnover and subcellular localization of beta-catenin by inhibiting its interaction with APC. Nat. Cell Biol. 3, 793–801. 10.1038/ncb0901-793 11533658

[B176] Sackmann E. K. Fulton A. L. Beebe D. J. (2014). The present and future role of microfluidics in biomedical research. Nature 507, 181–189. 10.1038/nature13118 24622198

[B177] Sadlonova A. Bowe D. B. Novak Z. Mukherjee S. Duncan V. E. Page G. P.. (2009). Identification of molecular distinctions between normal breast-associated fibroblasts and breast cancer-associated fibroblasts. Cancer Microenviron. 2, 9–21. 10.1007/s12307-008-0017-0 19308679 PMC2787925

[B178] Sakai M. Kato H. Sano A. Tanaka N. Inose T. Kimura H.. (2009). Expression of lysyl oxidase is correlated with lymph node metastasis and poor prognosis in esophageal squamous cell carcinoma. Ann. Surg. Oncol. 16, 2494–2501. 10.1245/s10434-009-0559-5 19526206

[B179] Salvador F. Martin A. Lopez-Menendez C. Moreno-Bueno G. Santos V. Vazquez-Naharro A.. (2017). Lysyl oxidase-like protein LOXL2 promotes lung metastasis of breast cancer. Cancer Res. 77, 5846–5859. 10.1158/0008-5472.CAN-16-3152 28720577 PMC5656180

[B180] Samavedi S. Joy N. (2017). 3D printing for the development of *in vitro* cancer models. Curr. Opin. Biomed. Eng. 2, 35–42. 10.1016/j.cobme.2017.06.003

[B181] Schedin P. Keely P. J. (2011). Mammary gland ECM remodeling, stiffness, and mechanosignaling in normal development and tumor progression. Cold Spring Harb. Perspect. Biol. 3:a003228. 10.1101/cshperspect.a003228 20980442 PMC3003460

[B182] Schrader J. Gordon-Walker T. T. Aucott R. L. Van Deemter M. Quaas A. Walsh S.. (2011). Matrix stiffness modulates proliferation, chemotherapeutic response, and dormancy in hepatocellular carcinoma cells. Hepatology 53, 1192–1205. 10.1002/hep.24108 21442631 PMC3076070

[B183] Seano G. Chiaverina G. Gagliardi P. A. Di Blasio L. Sessa R. Bussolino F.. (2013). Modeling human tumor angiogenesis in a three-dimensional culture system. Blood 121, e129–137. 10.1182/blood-2012-08-452292 23471306

[B184] Segreto F. Carotti S. Marangi G. F. Tosi D. Zingariello M. Pendolino A. L. (2017). The role of angiogenesis, inflammation and estrogen receptors in breast implant capsules development and remodeling. J. Plast. Reconstr. Aesthet. Surg. 12:3 10.1016/j.bjps.2017.12.003 29277501

[B185] Shi L. Wang L. Hou J. Zhu B. Min Z. Zhang M.. (2015). Targeting roles of inflammatory microenvironment in lung cancer and metastasis. Cancer Metastasis Rev. 34, 319–331. 10.1007/s10555-015-9570-4 26059063

[B186] Shiga K. Hara M. Nagasaki T. Sato T. Takahashi H. Takeyama H. (2015). Cancer-associated fibroblasts: their characteristics and their roles in tumor growth. Cancers (Basel). 7, 2443–2458. 10.3390/cancers7040902 26690480 PMC4695902

[B187] Shin J. W. Mooney D. J. (2016). Extracellular matrix stiffness causes systematic variations in proliferation and chemosensitivity in myeloid leukemias. Proc. Natl. Acad. Sci. U.S.A. 113, 12126–12131. 10.1073/pnas.1611338113 27790998 PMC5086998

[B188] Siefert S. A. Sarkar R. (2012). Matrix metalloproteinases in vascular physiology and disease. Vascular 20, 210–216. 10.1258/vasc.2011.201202 22896663

[B189] Skardal A. Zhang J. Mccoard L. Xu X. Oottamasathien S. Prestwich G. D. (2010). Photocrosslinkable hyaluronan-gelatin hydrogels for two-step bioprinting. Tissue Eng. A 16, 2675–2685. 10.1089/ten.tea.2009.0798 20387987 PMC3135254

[B190] Slater S. C. Beachley V. Hayes T. Zhang D. Welsh G. I. Saleem M. A.. (2011). An *in vitro* model of the glomerular capillary wall using electrospun collagen nanofibres in a bioartificial composite basement membrane. PLoS ONE 6:e20802. 10.1371/journal.pone.0020802 21731625 PMC3123297

[B191] Sobral J. M. Caridade S. G. Sousa R. A. Mano J. F. Reis R. L. (2011). Three-dimensional plotted scaffolds with controlled pore size gradients: effect of scaffold geometry on mechanical performance and cell seeding efficiency. Acta Biomater. 7, 1009–1018. 10.1016/j.actbio.2010.11.003 21056125

[B192] Soman P. Kelber J. A. Lee J. W. Wright T. N. Vecchio K. S. Klemke R. L.. (2012). Cancer cell migration within 3D layer-by-layer microfabricated photocrosslinked PEG scaffolds with tunable stiffness. Biomaterials 33, 7064–7070. 10.1016/j.biomaterials.2012.06.012 22809641 PMC3420339

[B193] Song H. H. Park K. M. Gerecht S. (2014). Hydrogels to model 3D *in vitro* microenvironment of tumor vascularization. Adv. Drug Deliv. Rev. 79–80, 19–29. 10.1016/j.addr.2014.06.002 PMC4258430 24969477

[B194] Sormunen R. T. Leong A. S. Vaaraniemi J. P. Fernando S. S. Eskelinen S. M. (1999). Immunolocalization of the fodrin, E-cadherin, and beta-catenin adhesion complex in infiltrating ductal carcinoma of the breast-comparison with an *in vitro* model. J Pathol 187, 416–423. 10.1002/(SICI)1096-9896(199903)187:4<416::AID-PATH255>3.0.CO;2-D 10398100

[B195] Sottile J. Hocking D. C. (2002). Fibronectin polymerization regulates the composition and stability of extracellular matrix fibrils and cell-matrix adhesions. Mol. Biol. Cell 13, 3546–3559. 10.1091/mbc.e02-01-0048 12388756 PMC129965

[B196] Sung K. E. Beebe D. J. (2014). Microfluidic 3D models of cancer. Adv. Drug Deliv. Rev. 79–80, 68–78. 10.1016/j.addr.2014.07.002 PMC4258433 25017040

[B197] Svensson G. Holtslag A. A. M. Kumar V. Mauritsen T. Steeneveld G. J. Angevine W. M. (2011). Evaluation of the diurnal cycle in the atmospheric boundary layer over land as represented by a variety of single-column models: the second GABLS experiment. Boundary Layer Meteorol. 140, 177–206. 10.1007/s10546-011-9611-7

[B198] Tasoglu S. Demirci U. (2013). Bioprinting for stem cell research. Trends Biotechnol. 31, 10–19. 10.1016/j.tibtech.2012.10.005 23260439 PMC3534918

[B199] Thoma C. R. Zimmermann M. Agarkova I. Kelm J. M. Krek W. (2014). 3D cell culture systems modeling tumor growth determinants in cancer target discovery. Adv. Drug Deliv. Rev. 69–70, 29–41. 10.1016/j.addr.2014.03.001 24636868

[B200] Thomas D. W. Burns J. Audette J. Carroll A. Dow-Hygelund C. Hay M. (2016). Clinical Development Success Rates 2006–2015. Washington, DC: Biotechnology Innovation Organization.

[B201] Trachtenberg J. E. Mountziaris P. M. Miller J. S. Wettergreen M. Kasper F. K. Mikos A. G. (2014). Open-source three-dimensional printing of biodegradable polymer scaffolds for tissue engineering. J. Biomed. Mater. Res. A 102, 4326–4335. 10.1002/jbm.a.35108 25493313 PMC4266185

[B202] Trachtenberg J. E. Santoro M. Williams C. Piard C. M. Smith B. T. Placone J. K. (2017). Effects of shear stress gradients on ewing sarcoma cells using 3D printed scaffolds and flow perfusion. ACS Biomater. Sci. Eng. 4, 347–356. 10.1021/acsbiomaterials.6b00641 33418729

[B203] Troup S. Njue C. Kliewer E. V. Parisien M. Roskelley C. Chakravarti S.. (2003). Reduced expression of the small leucine-rich proteoglycans, lumican, and decorin is associated with poor outcome in node-negative invasive breast cancer. Clin. Cancer Res. 9, 207–214. 12538471

[B204] Tsai H. F. Trubelja A. Shen A. Q. Bao G. (2017). Tumour-on-a-chip: microfluidic models of tumour morphology, growth and microenvironment. J. R. Soc. Interface 14:11 10.1098/rsif.2017.0137 PMC5493797 28637915

[B205] Verbridge S. S. Choi N. W. Zheng Y. Brooks D. J. Stroock A. D. Fischbach C. (2010). Oxygen-controlled three-dimensional cultures to analyze tumor angiogenesis. Tissue Eng. A 16, 2133–2141. 10.1089/ten.tea.2009.0670 20214469 PMC2947932

[B206] Wan L. Pantel K. Kang Y. (2013). Tumor metastasis: moving new biological insights into the clinic. Nat. Med. 19, 1450–1464. 10.1038/nm.3391 24202397

[B207] Wang C. Tang Z. Zhao Y. Yao R. Li L. Sun W. (2014). Three-dimensional *in vitro* cancer models: a short review. Biofabrication 6:022001. 10.1088/1758-5082/6/2/022001 24727833

[B208] Wei S. C. Fattet L. Tsai J. H. Guo Y. Pai V. H. Majeski H. E.. (2015). Matrix stiffness drives epithelial-mesenchymal transition and tumour metastasis through a TWIST1-G3BP2 mechanotransduction pathway. Nat. Cell Biol. 17, 678–688. 10.1038/ncb3157 25893917 PMC4452027

[B209] Williams C. B. Yeh E. S. Soloff A. C. (2016). Tumor-associated macrophages: unwitting accomplices in breast cancer malignancy. NPJ Breast Cancer 2:5. 10.1038/npjbcancer.2015.25 26998515 PMC4794275

[B210] Woo J. K. Jung H. J. Park J. Y. Kang J. H. Lee B. I. Shin D.. (2017). Daurinol blocks breast and lung cancer metastasis and development by inhibition of focal adhesion kinase (FAK). Oncotarget 8, 57058–57071. 10.18632/oncotarget.18983 28915654 PMC5593625

[B211] Wozniak M. A. Desai R. Solski P. A. Der C. J. Keely P. J. (2003). ROCK-generated contractility regulates breast epithelial cell differentiation in response to the physical properties of a three-dimensional collagen matrix. J. Cell Biol. 163, 583–595. 10.1083/jcb.200305010 14610060 PMC2173660

[B212] Wu J. Z. Yang T. J. Lu P. Ma W. (2014). Analysis of signaling pathways in recurrent breast cancer. Genet. Mol. Res. 13, 10097–10104. 10.4238/2014.December.4.4 25501221

[B213] Wyckoff J. B. Wang Y. Lin E. Y. Li J. F. Goswami S. Stanley E. R.. (2007). Direct visualization of macrophage-assisted tumor cell intravasation in mammary tumors. Cancer Res. 67, 2649–2656. 10.1158/0008-5472.CAN-06-1823 17363585

[B214] Xu F. Celli J. Rizvi I. Moon S. Hasan T. Demirci U. (2011). A three-dimensional *in vitro* ovarian cancer coculture model using a high-throughput cell patterning platform. Biotechnol. J. 6, 204–212. 10.1002/biot.201000340 21298805 PMC3780785

[B215] Ye J. Wu D. Wu P. Chen Z. Huang J. (2014). The cancer stem cell niche: cross talk between cancer stem cells and their microenvironment. Tumour Biol. 35, 3945–3951. 10.1007/s13277-013-1561-x 24420150

[B216] Yeh Y. C. Ling J. Y. Chen W. C. Lin H. H. Tang M. J. (2017). Mechanotransduction of matrix stiffness in regulation of focal adhesion size and number: reciprocal regulation of caveolin-1 and beta1 integrin. Sci. Rep. 7:15008. 10.1038/s41598-017-14932-6 29118431 PMC5678369

[B217] Yue X. Nguyen T. D. Zellmer V. Zhang S. Zorlutuna P. (2018). Stromal cell-laden 3D hydrogel microwell arrays as tumor microenvironment model for studying stiffness dependent stromal cell-cancer interactions. Biomaterials 170, 37–48. 10.1016/j.biomaterials.2018.04.001 29653286

[B218] Zahir N. Lakins J. N. Russell A. Ming W. Chatterjee C. Rozenberg G. I.. (2003). Autocrine laminin-5 ligates alpha6beta4 integrin and activates RAC and NFkappaB to mediate anchorage-independent survival of mammary tumors. J. Cell Biol. 163, 1397–1407. 10.1083/jcb.200302023 14691145 PMC2173718

[B219] Zervantonakis I. K. Hughes-Alford S. K. Charest J. L. Condeelis J. S. Gertler F. B. Kamm R. D. (2012). Three-dimensional microfluidic model for tumor cell intravasation and endothelial barrier function. Proc. Natl. Acad. Sci. U.S.A. 109, 13515–13520. 10.1073/pnas.1210182109 22869695 PMC3427099

[B220] Zhang Y. S. Duchamp M. Oklu R. Ellisen L. W. Langer R. Khademhosseini A. (2016). Bioprinting the Cancer Microenvironment. ACS Biomater Sci Eng 2, 1710–1721. 10.1021/acsbiomaterials.6b00246 28251176 PMC5328669

[B221] Zhao Y. Yao R. Ouyang L. Ding H. Zhang T. Zhang K.. (2014). Three-dimensional printing of Hela cells for cervical tumor model *in vitro*. Biofabrication 6:035001. 10.1088/1758-5082/6/3/035001 24722236

[B222] Zhou X. Zhu W. Nowicki M. Miao S. Cui H. Holmes B.. (2016). 3D Bioprinting a cell-laden bone matrix for breast cancer metastasis study. ACS Appl. Mater. Interfaces 8, 30017–30026. 10.1021/acsami.6b10673 27766838

