# Supplementary material for: Engineering Breast Cancer Microenvironments and 3D Bioprinting
Source: Front Bioeng Biotechnol. 2018 May 24;6:66. doi: 10.3389/fbioe.2018.00066 (PMC5978274; doi:10.3389/fbioe.2018.00066)
Supplement: Supplementary file 1 [file Data_Sheet_1.DOCX]

**Supplementary Information**

**Engineering breast cancer microenvironments and 3D bioprinting**

Jorge A. Belgodere^1^, Connor T. King^1^, Jacob B. Bursavich^1^, Matthew E. Burow^2^, Elizabeth C. Martin^1^, Jangwook P. Jung^1^

^1^ Department of Biological Engineering, Louisiana State University, Baton Rouge, LA, USA

^2^ Department of Medicine, Section Hematology/Oncology, Tulane University, New Orleans, LA, USA

**Supplementary References for Table 1.**

*Hallmark #1. Self-sufficiency in growth signals*

Akanuma, N., Hoshino, I., Akutsu, Y., Murakami, K., Isozaki, Y., Maruyama, T., Yusup, G., Qin, W., Toyozumi, T., and Takahashi, M. (2014). MicroRNA-133a regulates the mRNAs of two invadopodia-related proteins, FSCN1 and MMP14, in esophageal cancer. *Brt J Cancer* 110, 189-98.

Al-Alem, L.F., Mccord, L.A., Southard, R.C., Kilgore, M.W., and Curry Jr, T.E. (2013). Activation of the PKC pathway stimulates ovarian cancer cell proliferation, migration, and expression of MMP7 and MMP10. *Biol Reprod* 89, 73.

Alonso, S., Mayol, X., Nonell, L., Salvans, S., Pascual, M., and Pera, M. (2017). Peripheral blood leucocytes show differential expression of tumour progression‐related genes in colorectal cancer patients who have a postoperative intra‐abdominal infection: a prospective matched cohort study. *Colorectal Dis* 19. O115-125.

Bi, Q., Tang, S., Xia, L., Du, R., Fan, R., Gao, L., Jin, J., Liang, S., Chen, Z., and Xu, G. (2012). Ectopic expression of MiR-125a inhibits the proliferation and metastasis of hepatocellular carcinoma by targeting MMP11 and VEGF. *PLoS ONE* 7, e40169.

Canal, F., Anthony, E., Lescure, A., Del Nery, E., Camonis, J., Perez, F., Ragazzon, B., and Perret, C. (2015). A kinome siRNA screen identifies HGS as a potential target for liver cancers with oncogenic mutations in CTNNB1. *BMC Cancer* 15, 1020.

Cao, L., Chen, C., Zhu, H., Gu, X., Deng, D., Tian, X., Liu, J., and Xiao, Q. (2016). MMP16 is a marker of poor prognosis in gastric cancer promoting proliferation and invasion. *Oncotarget* 7, 51865-51874.

Cox, T.R., Bird, D., Baker, A.-M., Barker, H.E., Ho, M.W., Lang, G., and Erler, J.T. (2013). LOX-mediated collagen crosslinking is responsible for fibrosis-enhanced metastasis. *Cancer Res* 73, 1721-1732.

Eke, I., Deuse, Y., Hehlgans, S., Gurtner, K., Krause, M., Baumann, M., Shevchenko, A., Sandfort, V., and Cordes, N. (2012). β 1 Integrin/FAK/cortactin signaling is essential for human head and neck cancer resistance to radiotherapy. *J Clin Invest* 122, 1529-1540.

Gonzalez, M.E., Martin, E.E., Anwar, T., Arellano-Garcia, C., Medhora, N., Lama, A., Chen, Y.-C., Tanager, K.S., Yoon, E., and Kidwell, K.M. (2017). Mesenchymal stem cell-induced DDR2 mediates stromal-breast cancer interactions and metastasis growth. *Cell Rep* 18, 1215-1228.

Greish, K., Frandsen, J., Scharff, S., Gustafson, J., Cappello, J., Li, D., O'malley, B.W., and Ghandehari, H. (2010). Silk‐elastinlike protein polymers improve the efficacy of adenovirus thymidine kinase enzyme prodrug therapy of head and neck tumors. *J Gene Med* 12, 572-579.

Guedez, L., Jensen-Taubman, S., Bourboulia, D., Kwityn, C.J., Wei, B., Caterina, J., and Stetler-Stevenson, W.G. (2012). TIMP-2 targets tumor associated-myeloid suppressor cells with effects in cancer immune dysfunction and angiogenesis. *J Immunother* 35, 502-12.

Hou, T., Tong, C., Kazobinka, G., Zhang, W., Huang, X., Huang, Y., and Zhang, Y. (2016). Expression of COL6A1 predicts prognosis in cervical cancer patients. *Am J Transl Res* 8, 2838-44.

Huang, J., Zhang, J., Li, H., Lu, Z., Shan, W., Mercado-Uribe, I., and Liu, J. (2013). VCAM1 expression correlated with tumorigenesis and poor prognosis in high grade serous ovarian cancer. *Am J Transl Res* 5, 336-46.

Huang, M.-Y., Chang, H.-J., Chung, F.-Y., Yang, M.-J., Yang, Y.-H., Wang, J.-Y., and Lin, S.-R. (2010). MMP13 is a potential prognostic marker for colorectal cancer. *Oncol Rep* 24, 1241-1247.

Inagaki, Y., Shiraki, K., Sugimoto, K., Yada, T., Tameda, M., Ogura, S., Yamamoto, N., Takei, Y., and Ito, M. (2016). Epigenetic regulation of proliferation and invasion in hepatocellular carcinoma cells by CBP/p300 histone acetyltransferase activity. *Int J Oncol* 48, 533-540.

Jhunjhunwala, S., Jiang, Z., Stawiski, E.W., Gnad, F., Liu, J., Mayba, O., Du, P., Diao, J., Johnson, S., and Wong, K.-F. (2014). Diverse modes of genomic alteration in hepatocellular carcinoma. *Genome Biol* 15, 436.

Junnila, S., Kokkola, A., Mizuguchi, T., Hirata, K., Karjalainen‐Lindsberg, M.L., Puolakkainen, P., and Monni, O. (2010). Gene expression analysis identifies over‐expression of CXCL1, SPARC, SPP1, and SULF1 in gastric cancer. *Genes Chromosomes Cancer* 49, 28-39.

Keightley, M.C., Sales, K.J., and Jabbour, H.N. (2010). PGF 2α-F-prostanoid receptor signalling via ADAMTS1 modulates epithelial cell invasion and endothelial cell function in endometrial cancer. *BMC Cancer* 10, 488.

Koizume, S., Ito, S., Nakamura, Y., Yoshihara, M., Furuya, M., Yamada, R., Miyagi, E., Hirahara, F., Takano, Y., and Miyagi, Y. (2015). Lipid starvation and hypoxia synergistically activate ICAM1 and multiple genes in an Sp1-dependent manner to promote the growth of ovarian cancer. *Mol Cancer* 14, 77.

Kou, Y., Zhang, S., Zhao, B., Ding, R., Liu, H., and Li, S. (2013). Knockdown of MMP11 inhibits proliferation and invasion of gastric cancer cells. *Int J Immunopath* 26, 361-370.

Lv, F., Wang, J., Wu, Y., Chen, H., and Shen, X. (2015). Knockdown of MMP12 inhibits the growth and invasion of lung adenocarcinoma cells. *Int J Immunopathol Pharmacol* 28, 77-84.

Ma, Z.-H., Ma, J.-H., Jia, L., and Zhao, Y.-F. (2012). Effect of enhanced expression of COL8A1 on lymphatic metastasis of hepatocellular carcinoma in mice. *Exp Ther Med*  4, 621-626.

Maller, O., Hansen, K.C., Lyons, T.R., Acerbi, I., Weaver, V.M., Prekeris, R., Tan, A.-C., and Schedin, P. (2013). Collagen architecture in pregnancy-induced protection from breast cancer. *J Cell Sci* 126, 4108-4110.

Maxwell, E.G., Colquhoun, I.J., Chau, H.K., Hotchkiss, A.T., Waldron, K.W., Morris, V.J., and Belshaw, N.J. (2015). Rhamnogalacturonan I containing homogalacturonan inhibits colon cancer cell proliferation by decreasing ICAM1 expression. *Carbohydr Polym* 132, 546-553.

Meng, X., Chen, X., Lu, P., Ma, W., Yue, D., Song, L., and Fan, Q. (2016). MicroRNA-202 inhibits tumor progression by targeting LAMA1 in esophageal squamous cell carcinoma. *Biochem Biophys Res Comm* 473, 821-827.

Misawa, K., Kanazawa, T., Misawa, Y., Imai, A., Endo, S., Hakamada, K., and Mineta, H. (2012). Hypermethylation of collagen α2 (I) gene (COL1A2) is an independent predictor of survival in head and neck cancer. *Cancer Biomark* 10, 135-144.

Nishikawa, R., Goto, Y., Kojima, S., Enokida, H., Chiyomaru, T., Kinoshita, T., Sakamoto, S., Fuse, M., Nakagawa, M., and Naya, Y. (2014). Tumor-suppressive microRNA-29s inhibit cancer cell migration and invasion via targeting LAMC1 in prostate cancer. *Int J Oncol*  45, 401-410.

Sathyanarayana, U.G., Maruyama, R., Padar, A., Suzuki, M., Bondaruk, J., Sagalowsky, A., Minna, J.D., Frenkel, E.P., Grossman, H.B., and Czerniak, B. (2004). Molecular detection of noninvasive and invasive bladder tumor tissues and exfoliated cells by aberrant promoter methylation of laminin-5 encoding genes. *Cancer Res* 64, 1425-1430.

Sathyanarayana, U.G., Padar, A., Huang, C.X., Suzuki, M., Shigematsu, H., Bekele, B.N., and Gazdar, A.F. (2003). Aberrant promoter methylation and silencing of laminin-5-encoding genes in breast carcinoma. *Clin Cancer Res* 9, 6389-6394.

Shah, M., Huang, D., Blick, T., Connor, A., Reiter, L.A., Hardink, J.R., Lynch, C.C., Waltham, M., and Thompson, E.W. (2012). An MMP13-selective inhibitor delays primary tumor growth and the onset of tumor-associated osteolytic lesions in experimental models of breast cancer. *PLoS One* 7, e29615.

Shi, X., Chen, Z., Hu, X., Luo, M., Sun, Z., Li, J., Shi, S., Feng, X., Zhou, C., and Li, Z. (2016). AJUBA promotes the migration and invasion of esophageal squamous cell carcinoma cells through upregulation of MMP10 and MMP13 expression. *Oncotarget* 7, 36407.

Shukla, R., Chanda, N., Zambre, A., Upendran, A., Katti, K., Kulkarni, R.R., Nune, S.K., Casteel, S.W., Smith, C.J., and Vimal, J. (2012). Laminin receptor specific therapeutic gold nanoparticles (198AuNP-EGCg) show efficacy in treating prostate cancer. *Proc Natl Acad Sci USA*  109, 12426-12431.

Tang, J., Li, B., Hong, S., Liu, C., Min, J., Hu, M., Li, Y., Liu, Y., and Hong, L. (2017). Punicalagin suppresses the proliferation and invasion of cervical cancer cells through inhibition of the β-catenin pathway. *Mol Med Rep* 16, 1439-1444.

Wang, P.-C., Weng, C.-C., Hou, Y.-S., Jian, S.-F., Fang, K.-T., Hou, M.-F., and Cheng, K.-H. (2014). Activation of VCAM-1 and its associated molecule CD44 leads to increased malignant potential of breast cancer cells. *Int J Mol Sci* 15, 3560-3579.

Weng, T.-Y., Wang, C.-Y., Hung, Y.-H., Chen, W.-C., Chen, Y.-L., and Lai, M.-D. (2016). Differential expression pattern of THBS1 and THBS2 in lung cancer: clinical outcome and a systematic-analysis of microarray databases. *PLoS One* 11, e0161007.

Zhao, Y., Jia, L., Mao, X., Xu, H., Wang, B., and Liu, Y. (2009). siRNA‐targeted COL8A1 inhibits proliferation, reduces invasion and enhances sensitivity to D‐limonence treatment in hepatocarcinoma cells. *IUBMB Life* 61, 74-79.

Zhu, M., Zhang, N., He, S., Lui, Y., Lu, G., and Zhao, L. (2014). MicroRNA‐106a targets TIMP2 to regulate invasion and metastasis of gastric cancer. *FEBS Lett* 588, 600-607.

*Hallmark #2. Insensitivity to anti-growth signals*

Al-Alem, L.F., Mccord, L.A., Southard, R.C., Kilgore, M.W., and Curry Jr, T.E. (2013). Activation of the PKC pathway stimulates ovarian cancer cell proliferation, migration, and expression of MMP7 and MMP10. *Bio Repord* 89, 73.

Alonso, S., Mayol, X., Nonell, L., Salvans, S., Pascual, M., and Pera, M. (2017). Peripheral blood leucocytes show differential expression of tumour progression‐related genes in colorectal cancer patients who have a postoperative intra‐abdominal infection: a prospective matched cohort study. *Colorectal Dis* 19, O115-125.

Bi, Q., Tang, S., Xia, L., Du, R., Fan, R., Gao, L., Jin, J., Liang, S., Chen, Z., and Xu, G. (2012). Ectopic expression of MiR-125a inhibits the proliferation and metastasis of hepatocellular carcinoma by targeting MMP11 and VEGF. *PLoS One* 7, e40169.

Eke, I., Deuse, Y., Hehlgans, S., Gurtner, K., Krause, M., Baumann, M., Shevchenko, A., Sandfort, V., and Cordes, N. (2012). β 1 Integrin/FAK/cortactin signaling is essential for human head and neck cancer resistance to radiotherapy. *J Clin Invest* 122, 1529-1540.

Gan, R., Yang, Y., Yang, X., Zhao, L., Lu, J., and Meng, Q. (2014). Downregulation of miR-221/222 enhances sensitivity of breast cancer cells to tamoxifen through upregulation of TIMP 3. *Cancer Gene Ther* 21, 290-6.

Greish, K., Frandsen, J., Scharff, S., Gustafson, J., Cappello, J., Li, D., O'malley, B.W., and Ghandehari, H. (2010). Silk‐elastinlike protein polymers improve the efficacy of adenovirus thymidine kinase enzyme prodrug therapy of head and neck tumors. *J Gene Med* 12, 572-579.

Guedez, L., Jensen-Taubman, S., Bourboulia, D., Kwityn, C.J., Wei, B., Caterina, J., and Stetler-Stevenson, W.G. (2012). TIMP-2 targets tumor associated-myeloid suppressor cells with effects in cancer immune dysfunction and angiogenesis. *J Immunother* 35, 502-12.

Hou, T., Tong, C., Kazobinka, G., Zhang, W., Huang, X., Huang, Y., and Zhang, Y. (2016). Expression of COL6A1 predicts prognosis in cervical cancer patients. *Am J Transl Res* 8, 2838-44.

Huang, J., Zhang, J., Li, H., Lu, Z., Shan, W., Mercado-Uribe, I., and Liu, J. (2013). VCAM1 expression correlated with tumorigenesis and poor prognosis in high grade serous ovarian cancer. *Am J Transl Res* 5, 336-46.

Huang, M.-Y., Chang, H.-J., Chung, F.-Y., Yang, M.-J., Yang, Y.-H., Wang, J.-Y., and Lin, S.-R. (2010). MMP13 is a potential prognostic marker for colorectal cancer. *Oncol Rep* 24, 1241-1247.

Keightley, M.C., Sales, K.J., and Jabbour, H.N. (2010). PGF 2α-F-prostanoid receptor signalling via ADAMTS1 modulates epithelial cell invasion and endothelial cell function in endometrial cancer. *BMC Cancer* 10, 488.

Koizume, S., Ito, S., Nakamura, Y., Yoshihara, M., Furuya, M., Yamada, R., Miyagi, E., Hirahara, F., Takano, Y., and Miyagi, Y. (2015). Lipid starvation and hypoxia synergistically activate ICAM1 and multiple genes in an Sp1-dependent manner to promote the growth of ovarian cancer. *Mol Cancer* 14, 77.

Lv, F., Wang, J., Wu, Y., Chen, H., and Shen, X. (2015). Knockdown of MMP12 inhibits the growth and invasion of lung adenocarcinoma cells. *Int J Immunopathol Pharmacol* 28, 77-84.

Maxwell, E.G., Colquhoun, I.J., Chau, H.K., Hotchkiss, A.T., Waldron, K.W., Morris, V.J., and Belshaw, N.J. (2015). Rhamnogalacturonan I containing homogalacturonan inhibits colon cancer cell proliferation by decreasing ICAM1 expression. *Carbohydra Polym* 132, 546-553.

Sathyanarayana, U.G., Maruyama, R., Padar, A., Suzuki, M., Bondaruk, J., Sagalowsky, A., Minna, J.D., Frenkel, E.P., Grossman, H.B., and Czerniak, B. (2004). Molecular detection of noninvasive and invasive bladder tumor tissues and exfoliated cells by aberrant promoter methylation of laminin-5 encoding genes. *Cancer Res* 64, 1425-1430.

Sathyanarayana, U.G., Padar, A., Huang, C.X., Suzuki, M., Shigematsu, H., Bekele, B.N., and Gazdar, A.F. (2003). Aberrant promoter methylation and silencing of laminin-5-encoding genes in breast carcinoma. *Clin Cancer Res* 9, 6389-6394.

Shah, M., Huang, D., Blick, T., Connor, A., Reiter, L.A., Hardink, J.R., Lynch, C.C., Waltham, M., and Thompson, E.W. (2012). An MMP13-selective inhibitor delays primary tumor growth and the onset of tumor-associated osteolytic lesions in experimental models of breast cancer. *PLoS One* 7, e29615.

Shi, X., Chen, Z., Hu, X., Luo, M., Sun, Z., Li, J., Shi, S., Feng, X., Zhou, C., and Li, Z. (2016). AJUBA promotes the migration and invasion of esophageal squamous cell carcinoma cells through upregulation of MMP10 and MMP13 expression. *Oncotarget* 7, 36407-18.

Wang, P.-C., Weng, C.-C., Hou, Y.-S., Jian, S.-F., Fang, K.-T., Hou, M.-F., and Cheng, K.-H. (2014). Activation of VCAM-1 and its associated molecule CD44 leads to increased malignant potential of breast cancer cells. *Int J Mol Sci* 15, 3560-3579.

Weng, T.-Y., Wang, C.-Y., Hung, Y.-H., Chen, W.-C., Chen, Y.-L., and Lai, M.-D. (2016). Differential expression pattern of THBS1 and THBS2 in lung cancer: clinical outcome and a systematic-analysis of microarray databases. *PLoS one* 11, e0161007.

*Hallmark #3. Evading programmed cell death*

Brown, C.W., Brodsky, A.S., and Freiman, R.N. (2015). Notch3 overexpression promotes anoikis resistance in epithelial ovarian cancer via upregulation of COL4A2. *Mol Cancer Res* 13, 78-85.

Canal, F., Anthony, E., Lescure, A., Del Nery, E., Camonis, J., Perez, F., Ragazzon, B., and Perret, C. (2015). A kinome siRNA screen identifies HGS as a potential target for liver cancers with oncogenic mutations in CTNNB1. *BMC cancer* 15, 1020.

Chen, Q., Zhang, X.H.-F., and Massagué, J. (2011). Macrophage binding to receptor VCAM-1 transmits survival signals in breast cancer cells that invade the lungs. *Cancer Cell* 20, 538-549.

Chen, X., Zhu, H., Wu, X., Xie, X., Huang, G., Xu, X., Li, S., and Xing, C. (2016). Downregulated pseudogene CTNNAP1 promote tumor growth in human cancer by downregulating its cognate gene CTNNA1 expression. *Oncotarget* 7, 55518-28.

Choi, G.C., Li, J., Wang, Y., Li, L., Zhong, L., Ma, B., Su, X., Ying, J., Xiang, T., and Rha, S.Y. (2014). The metalloprotease ADAMTS8 displays antitumor properties through antagonizing EGFR–MEK–ERK signaling and is silenced in carcinomas by CpG methylation. *Mol Cancer Res* 12, 228-238.

Inagaki, Y., Shiraki, K., Sugimoto, K., Yada, T., Tameda, M., Ogura, S., Yamamoto, N., Takei, Y., and Ito, M. (2016). Epigenetic regulation of proliferation and invasion in hepatocellular carcinoma cells by CBP/p300 histone acetyltransferase activity. *Int J Oncol* 48, 533-540.

Jingsong, H., Hong, G., Yang, J., Duo, Z., Li, F., Weicai, C., Xueying, L., Yousheng, M., Yiwen, O., and Yue, P. (2017). siRNA-Mediated suppression of collagen type iv alpha 2 (COL4A2) mRNA inhibits triple-negative breast cancer cell proliferation and migration. *Oncotarget* 8, 2585-93.

Joshi, P., Jeon, Y.-J., Laganà, A., Middleton, J., Secchiero, P., Garofalo, M., and Croce, C.M. (2015). MicroRNA-148a reduces tumorigenesis and increases TRAIL-induced apoptosis in NSCLC. *Proc Natl Acad Sci USA* 112, 8650-8655.

Kumazoe, M., Sugihara, K., Tsukamoto, S., Huang, Y., Tsurudome, Y., Suzuki, T., Suemasu, Y., Ueda, N., Yamashita, S., and Kim, Y. (2013). 67-kDa laminin receptor increases cGMP to induce cancer-selective apoptosis. *J Clin Invest* 123, 787-99

Meng, X., Chen, X., Lu, P., Ma, W., Yue, D., Song, L., and Fan, Q. (2016). MicroRNA-202 inhibits tumor progression by targeting LAMA1 in esophageal squamous cell carcinoma. *Biochem Biophys Res Comm* 473, 821-827.

Pasqualini, L., Bu, H., Puhr, M., Narisu, N., Rainer, J., Schlick, B., Schäfer, G., Angelova, M., Trajanoski, Z., and Börno, S.T. (2015). miR-22 and miR-29a are members of the androgen receptor cistrome modulating LAMC1 and Mcl-1 in prostate cancer. *Mol Endocrinol* 29, 1037-1054.

Ryu, J.S., Mikecin, A.-M., and Raucher, D. (2013). The effects of a cell cycle inhibitory peptide fused to elastin-like polypeptide on pancreatic cancer cells with hyperthermia and its combination with gemcitabine. *Cancer Res*, 73, 2062 (Supplement)

Shi, X., Chen, Z., Hu, X., Luo, M., Sun, Z., Li, J., Shi, S., Feng, X., Zhou, C., and Li, Z. (2016). AJUBA promotes the migration and invasion of esophageal squamous cell carcinoma cells through upregulation of MMP10 and MMP13 expression. *Oncotarget* 7, 36407-18.

Tang, J., Li, B., Hong, S., Liu, C., Min, J., Hu, M., Li, Y., Liu, Y., and Hong, L. (2017). Punicalagin suppresses the proliferation and invasion of cervical cancer cells through inhibition of the β-catenin pathway. *Mol Med Rep* 16, 1439-1444.

Xiao, X.-Y., Wang, X.-D., and Zang, D.-Y. (2012). MMP1-1607 1G/2G polymorphism and lung cancer risk: a meta-analysis. *Tumor Biol* 33, 2385-2392.

Zhu, Y.-P., Wan, F.-N., Shen, Y.-J., Wang, H.-K., Zhang, G.-M., and Ye, D.-W. (2015). Reactive stroma component COL6A1 is upregulated in castration-resistant prostate cancer and promotes tumor growth. *Oncotarget* 6, 14488-96.

*Hallmark #4. Limitless replicative potential*

Zhu, Y.-P., Wan, F.-N., Shen, Y.-J., Wang, H.-K., Zhang, G.-M., and Ye, D.-W. (2015). Reactive stroma component COL6A1 is upregulated in castration-resistant prostate cancer and promotes tumor growth. *Oncotarget* 6, 14488-96.

*Hallmark #5. Sustained angiogenesis*

Chen, J., Zhang, J., Li, X., Zhang, C., Zhang, H., Jin, J., and Dai, D. (2016). Downregulation of ADAMTS8 by DNA Hypermethylation in Gastric Cancer and Its Clinical Significance. *BioMed Res Int* 2016, 5083841

Chen, J., Zhi, Y., Chang, X., Zhang, S., and Dai, D. (2013). Expression of ADAMTS1 and its correlation with angiogenesis in primary gastric cancer and lymph node metastasis. *Dig Dis Sci* 58, 405-413.

Chen, L., Xiao, Z., Meng, Y., Zhao, Y., Han, J., Su, G., Chen, B., and Dai, J. (2012). The enhancement of cancer stem cell properties of MCF-7 cells in 3D collagen scaffolds for modeling of cancer and anti-cancer drugs. *Biomaterials* 33, 1437-1444.

Chen, Q., Lu, G., Cai, Y., Li, Y., Xu, R., Ke, Y., and Zhang, S. (2014). MiR-124-5p inhibits the growth of high-grade gliomas through posttranscriptional regulation of LAMB1. *Neuro Oncol* 16, 637-651.

Choi, G.C., Li, J., Wang, Y., Li, L., Zhong, L., Ma, B., Su, X., Ying, J., Xiang, T., and Rha, S.Y. (2014). The metalloprotease ADAMTS8 displays antitumor properties through antagonizing EGFR–MEK–ERK signaling and is silenced in carcinomas by CpG methylation. *Mol Cancer Res* 12, 228-238.

Choi, J.E., Kim, D.S., Kim, E.J., Chae, M.H., Cha, S.I., Kim, C.H., Jheon, S., Jung, T.H., and Park, J.Y. (2008). Aberrant methylation of ADAMTS1 in non-small cell lung cancer. *Cancer Genet Cytogenet* 187, 80-84.

Dai, J., Lin, Y., Duan, Y., Li, Z., Zhou, D., Chen, W., Wang, L., and Zhang, Q.-Q. (2017). Andrographolide Inhibits Angiogenesis by Inhibiting the Mir-21-5p/TIMP3 Signaling Pathway. *Int J Biol Sci* 13, 660-668.

Dredge, K., Hammond, E., Davis, K., Li, C.P., Liu, L., Johnstone, K., Handley, P., Wimmer, N., Gonda, T., and Gautam, A. (2010). The PG500 series: novel heparan sulfate mimetics as potent angiogenesis and heparanase inhibitors for cancer therapy. *Invest New Drugs* 28, 276-283.

Guedez, L., Jensen-Taubman, S., Bourboulia, D., Kwityn, C.J., Wei, B., Caterina, J., and Stetler-Stevenson, W.G. (2012). TIMP-2 targets tumor associated-myeloid suppressor cells with effects in cancer immune dysfunction and angiogenesis. *J Immunother* 35, 502-512.

Guo, X., Zhu, X., Zhao, L., Li, X., Cheng, D., and Feng, K. (2017). Tumor-associated calcium signal transducer 2 regulates neovascularization of non-small-cell lung cancer via activating ERK1/2 signaling pathway. *Tumor Biol* 39, 1010428317694324.

Kimura, K., Nakayama, M., Naito, I., Komiyama, T., Ichimura, K., Asano, H., Tsukuda, K., Ohtsuka, A., Oohashi, T., and Miyoshi, S. (2016). Human collagen XV is a prominent histopathological component of sinusoidal capillarization in hepatocellular carcinogenesis. *Int J Clin Oncol* 21, 302-309.

Kumar, S., Rao, N., and Ge, R. (2012). Emerging roles of ADAMTSs in angiogenesis and cancer. *Cancers* 4, 1252-1299.

Lee, Y.-J., Koch, M., Karl, D., Torres-Collado, A.X., Fernando, N.T., Rothrock, C., Kuruppu, D., Ryeom, S., Iruela-Arispe, M.L., and Yoon, S.S. (2010). Variable inhibition of thrombospondin 1 against liver and lung metastases through differential activation of metalloproteinase ADAMTS1. *Cancer Res* 70, 948-956.

Liang, J.-F., Wang, H.-K., Xiao, H., Li, N., Cheng, C.-X., Zhao, Y.-Z., Ma, Y.-B., Gao, J.-Z., Bai, R.-B., and Zheng, H.-X. (2010). Relationship and prognostic significance of SPARC and VEGF protein expression in colon cancer. *J Exp Clin Cancer Res* 29, 71.

Musumeci, G., Castorina, A., Magro, G., Cardile, V., Castorina, S., and Ribatti, D. (2015). Enhanced expression of CD31/platelet endothelial cell adhesion molecule 1 (PECAM1) correlates with hypoxia inducible factor-1 alpha (HIF-1α) in human glioblastoma multiforme. *Exp Cell Res* 339, 407-416.

Xiao, X.-Y., Wang, X.-D., and Zang, D.-Y. (2012). MMP1-1607 1G/2G polymorphism and lung cancer risk: a meta-analysis. *Tumor Biol* 33, 2385-2392.

Yeung, T.-L., Ghosh, S., Wong, K.-K., Birrer, M.J., and Mok, S.C. (2011). Up-regulation of stromal versican in advanced stage serous ovarian cancer modifies tumor microenvironment and promotes cancer invasion. *Cancer Res* 71, 420 (Supplement).

*Hallmark #6. Tissue invasion and metastasis*

Akanuma, N., Hoshino, I., Akutsu, Y., Murakami, K., Isozaki, Y., Maruyama, T., Yusup, G., Qin, W., Toyozumi, T., and Takahashi, M. (2014). MicroRNA-133a regulates the mRNAs of two invadopodia-related proteins, FSCN1 and MMP14, in esophageal cancer. *Br J Cancer* 110, 189-98.

Al-Alem, L.F., Mccord, L.A., Southard, R.C., Kilgore, M.W., and Curry Jr, T.E. (2013). Activation of the PKC pathway stimulates ovarian cancer cell proliferation, migration, and expression of MMP7 and MMP10. *Biol Reprod* 89, 73.

Alonso, S., Mayol, X., Nonell, L., Salvans, S., Pascual, M., and Pera, M. (2017). Peripheral blood leucocytes show differential expression of tumour progression‐related genes in colorectal cancer patients who have a postoperative intra‐abdominal infection: a prospective matched cohort study. *Colorectal Dis* 19. O115-125.

Bae, G.-Y., Choi, S.-J., Lee, J.-S., Jo, J., Lee, J., Kim, J., and Cha, H.-J. (2013). Loss of E-cadherin activates EGFR-MEK/ERK signaling, which promotes invasion via the ZEB1/MMP2 axis in non-small cell lung cancer. *Oncotarget* 4, 2512-22.

Bauer, R., Ratzinger, S., Wales, L., Bosserhoff, A., Senner, V., Grifka, J., and Grässel, S. (2011). Inhibition of collagen XVI expression reduces glioma cell invasiveness. *Cell Physiol Biochem* 27, 217-226.

Bi, Q., Tang, S., Xia, L., Du, R., Fan, R., Gao, L., Jin, J., Liang, S., Chen, Z., and Xu, G. (2012). Ectopic expression of MiR-125a inhibits the proliferation and metastasis of hepatocellular carcinoma by targeting MMP11 and VEGF. *PLoS One* 7, e40169.

Bolignano, D., Donato, V., Lacquaniti, A., Fazio, M.R., Bono, C., Coppolino, G., and Buemi, M. (2010). Neutrophil gelatinase-associated lipocalin (NGAL) in human neoplasias: a new protein enters the scene. *Cancer Lett* 288, 10-16.

Cao, L., Chen, C., Zhu, H., Gu, X., Deng, D., Tian, X., Liu, J., and Xiao, Q. (2016). MMP16 is a marker of poor prognosis in gastric cancer promoting proliferation and invasion. *Oncotarget* 7, 51865-51874.

Chakravarthi, B.V., Pathi, S.S., Goswami, M.T., Cieślik, M., Zheng, H., Nallasivam, S., Arekapudi, S.R., Jing, X., Siddiqui, J., and Athanikar, J. (2014). The miR-124-prolyl hydroxylase P4HA1-MMP1 axis plays a critical role in prostate cancer progression. *Oncotarget* 5, 6654-69.

Chen, A., Beetham, H., Black, M.A., Priya, R., Telford, B.J., Guest, J., Wiggins, G.A., Godwin, T.D., and Guilford, P.J. (2014). E-cadherin loss alters cytoskeletal organization and adhesion in non-malignant breast cells but is insufficient to induce an epithelial-mesenchymal transition. *BMC Cancer* 14, 552.

Chen, B., Huang, Z., Zhang, Y., Chen, Y., and Li, Z. (2015). MicroRNA-145 suppresses osteosarcoma metastasis via targeting MMP16. *Cell Physiol Biochem* 37, 2183-2193.

Chen, D., Bhat-Nakshatri, P., Goswami, C., Badve, S., and Nakshatri, H. (2013). ANTXR1, a stem cell-enriched functional biomarker, connects collagen signaling to cancer stem-like cells and metastasis in breast cancer. *Cancer Res* 73, 5821-5833.

Chen, J.-S., Huang, X.-H., Wang, Q., Huang, J.-Q., Zhang, L.-J., Chen, X.-L., Lei, J., and Cheng, Z.-X. (2012). Sonic hedgehog signaling pathway induces cell migration and invasion through focal adhesion kinase/AKT signaling-mediated activation of matrix metalloproteinase (MMP)-2 and MMP-9 in liver cancer. *Carcinogenesis* 34, 10-19.

Chimal-Ramírez, G., Espinoza-Sánchez, N., Utrera-Barillas, D., Benítez-Bribiesca, L., Velázquez, J., Arriaga-Pizano, L., Monroy-García, A., Reyes-Maldonado, E., Dominguez-Lopez, M.L., and Piña-Sánchez, P. (2013). MMP1, MMP9, and COX2 expressions in promonocytes are induced by breast cancer cells and correlate with collagen degradation, transformation-like morphological changes in MCF-10A acini, and tumor aggressiveness. *Biomed Res Int*  2013, 279505.

Choi, G.C., Li, J., Wang, Y., Li, L., Zhong, L., Ma, B., Su, X., Ying, J., Xiang, T., and Rha, S.Y. (2014). The metalloprotease ADAMTS8 displays antitumor properties through antagonizing EGFR–MEK–ERK signaling and is silenced in carcinomas by CpG methylation. *Mol Cancer Res* 12, 228-238.

Datar, I., Feng, J., Qiu, X., Lewandowski, J., Yeung, M., Ren, G., Aras, S., Al-Mulla, F., Cui, H., and Trumbly, R. (2015). RKIP inhibits local breast cancer invasion by antagonizing the transcriptional activation of MMP13. *PLoS One* 10, e0134494.

Desai, K., Nair, M.G., Prabhu, J.S., Vinod, A., Korlimarla, A., Rajarajan, S., Aiyappa, R., Kaluve, R.S., Alexander, A., and Hari, P. (2016). High expression of integrin β6 in association with the Rho–Rac pathway identifies a poor prognostic subgroup within HER2 amplified breast cancers. *Cancer Med* 5, 2000-2011.

Drake, J.M., Barnes, J.M., Madsen, J.M., Domann, F.E., Stipp, C.S., and Henry, M.D. (2010). ZEB1 coordinately regulates laminin-332 and β4 integrin expression altering the invasive phenotype of prostate cancer cells. *J Biol Chem* 285, 33940-33948.

Fernandez‐Garcia, B., Eiró, N., Marín, L., González‐Reyes, S., González, L.O., Lamelas, M.L., and Vizoso, F.J. (2014). Expression and prognostic significance of fibronectin and matrix metalloproteases in breast cancer metastasis. *Histopathology* 64, 512-522.

Fletcher, S.J., Sacca, P.A., Pistone-Creydt, M., Coló, F.A., Serra, M.F., Santino, F.E., Sasso, C.V., Lopez-Fontana, C.M., Carón, R.W., and Calvo, J.C. (2017). Human breast adipose tissue: characterization of factors that change during tumor progression in human breast cancer. *J Exp Clin Cancer Res* 36, 26.

Gilkes, D.M., Chaturvedi, P., Bajpai, S., Wong, C.C., Wei, H., Pitcairn, S., Hubbi, M.E., Wirtz, D., and Semenza, G.L. (2013). Collagen prolyl hydroxylases are essential for breast cancer metastasis. *Cancer Res* 73, 3285-3296.

Giudice, F.S., Pinto Jr, D.S., Nör, J.E., Squarize, C.H., and Castilho, R.M. (2013). Inhibition of histone deacetylase impacts cancer stem cells and induces epithelial-mesenchyme transition of head and neck cancer. *PLoS One* 8, e58672.

Gonzalez, M.E., Martin, E.E., Anwar, T., Arellano-Garcia, C., Medhora, N., Lama, A., Chen, Y.-C., Tanager, K.S., Yoon, E., and Kidwell, K.M. (2017). Mesenchymal stem cell-induced DDR2 mediates stromal-breast cancer interactions and metastasis growth. *Cell Rep* 18, 1215-1228.

Han, H.B., Gu, J., Zuo, H.J., Chen, Z.G., Zhao, W., Li, M., Ji, D.B., Lu, Y.Y., and Zhang, Z.Q. (2012). Let‐7c functions as a metastasis suppressor by targeting MMP11 and PBX3 in colorectal cancer. *J Pathol* 226, 544-555.

Hart, K., Landvik, N.E., Lind, H., Skaug, V., Haugen, A., and Zienolddiny, S. (2011). A combination of functional polymorphisms in the CASP8, MMP1, IL10 and SEPS1 genes affects risk of non-small cell lung cancer. *Lung Cancer* 71, 123-129.

Hou, T., Tong, C., Kazobinka, G., Zhang, W., Huang, X., Huang, Y., and Zhang, Y. (2016). Expression of COL6A1 predicts prognosis in cervical cancer patients. *Am J Transl Res* 8, 2838-44.

Huang, M.-Y., Chang, H.-J., Chung, F.-Y., Yang, M.-J., Yang, Y.-H., Wang, J.-Y., and Lin, S.-R. (2010). MMP13 is a potential prognostic marker for colorectal cancer. *Oncology Rep* 24, 1241-1247.

Hung, W.-C., Tseng, W.-L., Shiea, J., and Chang, H.-C. (2010). Skp2 overexpression increases the expression of MMP-2 and MMP-9 and invasion of lung cancer cells. *Cancer Lett* 288, 156-161.

Inagaki, Y., Shiraki, K., Sugimoto, K., Yada, T., Tameda, M., Ogura, S., Yamamoto, N., Takei, Y., and Ito, M. (2016). Epigenetic regulation of proliferation and invasion in hepatocellular carcinoma cells by CBP/p300 histone acetyltransferase activity. *Int J Oncol* 48, 533-540.

Jia, L.-F., Wei, S.-B., Mitchelson, K., Gao, Y., Zheng, Y.-F., Meng, Z., Gan, Y.-H., and Yu, G.-Y. (2014). miR-34a inhibits migration and invasion of tongue squamous cell carcinoma via targeting MMP9 and MMP14. *PLoS One* 9, e108435.

Jingsong, H., Hong, G., Yang, J., Duo, Z., Li, F., Weicai, C., Xueying, L., Yousheng, M., Yiwen, O., and Yue, P. (2017). siRNA-Mediated suppression of collagen type iv alpha 2 (COL4A2) mRNA inhibits triple-negative breast cancer cell proliferation and migration. *Oncotarget* 8, 2585-93.

Junnila, S., Kokkola, A., Mizuguchi, T., Hirata, K., Karjalainen‐Lindsberg, M.L., Puolakkainen, P., and Monni, O. (2010). Gene expression analysis identifies over‐expression of CXCL1, SPARC, SPP1, and SULF1 in gastric cancer. *Genes Chromosomes Cancer* 49, 28-39.

Karagiannis, G.S., Petraki, C., Prassas, I., Saraon, P., Musrap, N., Dimitromanolakis, A., and Diamandis, E.P. (2012). Proteomic signatures of the desmoplastic invasion front reveal collagen type XII as a marker of myofibroblastic differentiation during colorectal cancer metastasis. *Oncotarget* 3, 267-85.

Kashima, H., Wu, R.-C., Wang, Y., Sinno, A.K., Miyamoto, T., Shiozawa, T., Wang, T.-L., Fader, A.N., and Shih, I.-M. (2015). Laminin C1 expression by uterine carcinoma cells is associated with tumor progression. *Gynecol Oncol* 139, 338-344.

Keightley, M.C., Sales, K.J., and Jabbour, H.N. (2010). PGF 2α-F-prostanoid receptor signalling via ADAMTS1 modulates epithelial cell invasion and endothelial cell function in endometrial cancer. *BMC Cancer* 10, 488.

Kinoshita, T., Hanazawa, T., Nohata, N., Kikkawa, N., Enokida, H., Yoshino, H., Yamasaki, T., Hidaka, H., Nakagawa, M., and Okamoto, Y. (2012). Tumor suppressive microRNA-218 inhibits cancer cell migration and invasion through targeting laminin-332 in head and neck squamous cell carcinoma. *Oncotarget* 3, 1386-400.

Klupp, F., Neumann, L., Kahlert, C., Diers, J., Halama, N., Franz, C., Schmidt, T., Koch, M., Weitz, J., and Schneider, M. (2016). Serum MMP7, MMP10 and MMP12 level as negative prognostic markers in colon cancer patients. *BMC Cancer* 16, 494.

Koskimaki, J.E., Karagiannis, E.D., Tang, B.C., Hammers, H., Watkins, D.N., Pili, R., and Popel, A.S. (2010). Pentastatin-1, a collagen IV derived 20-mer peptide, suppresses tumor growth in a small cell lung cancer xenograft model. *BMC Cancer* 10, 29.

Kou, Y., Zhang, S., Zhao, B., Ding, R., Liu, H., and Li, S. (2013). Knockdown of MMP11 inhibits proliferation and invasion of gastric cancer cells. *Int J Immunopathol Pharmacol* 26, 361-370.

Lee, J., Jin, H., Lee, W.S., Nagappan, A., Choi, Y., Kim, G., Jung, J., Ryu, C.H., Shin, S.C., and Hong, S.C. (2016). Morin, a Flavonoid from Moraceae, Inhibits Cancer Cell Adhesion to Endothelial Cells and EMT by Downregulating VCAM1 and Ncadherin. *Asian Pacific J Cancer Prev* 17, 3071-3075.

Li, R., Ochs, M.F., Ahn, S.M., Hennessey, P., Tan, M., Soudry, E., Gaykalova, D.A., Uemura, M., Brait, M., and Shao, C. (2014). Expression microarray analysis reveals alternative splicing of LAMA3 and DST genes in head and neck squamous cell carcinoma. *PLoS One* 9, e91263.

Li, T., Xie, J., Shen, C., Cheng, D., Shi, Y., Wu, Z., Zhan, Q., Deng, X., Chen, H., and Shen, B. (2014). miR-150-5p inhibits hepatoma cell migration and invasion by targeting MMP14. *PLoS One* 9, e115577.

Lin, C., Lin, W., Yeh, S., Li, L., and Chang, C. (2015). Infiltrating neutrophils increase bladder cancer cell invasion via modulation of androgen receptor (AR)/MMP13 signals. *Oncotarget* 6, 43081-9.

Lin, F., Wang, X., Jie, Z., Hong, X., Li, X., Wang, M., and Yu, Y. (2011). Inhibitory effects of miR-146b-5p on cell migration and invasion of pancreatic cancer by targeting MMP16. *J Huazhong Univ Sci Technolog Med Sci* 31, 509-14.

Liu, G., Jiang, C., Li, D., Wang, R., and Wang, W. (2014). MiRNA-34a inhibits EGFR-signaling-dependent MMP7 activation in gastric cancer. *Tumor Biol* 35, 9801-9806.

Liu, L., Sun, L., Zhao, P., Yao, L., Jin, H., Liang, S., Wang, Y., Zhang, D., Pang, Y., and Shi, Y. (2010). Hypoxia promotes metastasis in human gastric cancer by up‐regulating the 67‐kDa laminin receptor. *Cancer Sci* 101, 1653-1660.

Liu, Y., Xu, Y., Guo, S., and Chen, H. (2016). T cell factor-4 functions as a co-activator to promote NF-κB-dependent MMP-15 expression in lung carcinoma cells. *Sci Rep* 6, 24025.

Lu, H., Hu, L., Yu, L., Wang, X., Urvalek, A.M., Li, T., Shen, C., Mukherjee, D., Lahiri, S.K., and Wason, M.S. (2014). KLF8 and FAK cooperatively enrich the active MMP14 on the cell surface required for the metastatic progression of breast cancer. *Oncogene* 33, 2909-17.

Lu, X., Mu, E., Wei, Y., Riethdorf, S., Yang, Q., Yuan, M., Yan, J., Hua, Y., Tiede, B.J., and Lu, X. (2011). VCAM-1 promotes osteolytic expansion of indolent bone micrometastasis of breast cancer by engaging α4β1-positive osteoclast progenitors. *Cancer Cell* 20, 701-714.

Lv, F., Wang, J., Wu, Y., Chen, H., and Shen, X. (2015). Knockdown of MMP12 inhibits the growth and invasion of lung adenocarcinoma cells. *Int J Immunopathol Pharmacol* 28, 77-84.

Ma, Z.-H., Ma, J.-H., Jia, L., and Zhao, Y.-F. (2012). Effect of enhanced expression of COL8A1 on lymphatic metastasis of hepatocellular carcinoma in mice. *Exp Ther Med* 4, 621-626.

Maller, O., Hansen, K.C., Lyons, T.R., Acerbi, I., Weaver, V.M., Prekeris, R., Tan, A.-C., and Schedin, P. (2013). Collagen architecture in pregnancy-induced protection from breast cancer. *J Cell Sci* 126, 4108-4110.

Misawa, K., Kanazawa, T., Misawa, Y., Imai, A., Endo, S., Hakamada, K., and Mineta, H. (2012). Hypermethylation of collagen α2 (I) gene (COL1A2) is an independent predictor of survival in head and neck cancer. *Cancer Biomark* 10, 135-144.

Mitsui, Y., Shiina, H., Kato, T., Maekawa, S., Hashimoto, Y., Shiina, M., Imai-Sumida, M., Kulkarni, P., Dasgupta, P., and Wong, R.K. (2017). Versican promotes tumor progression, metastasis and predicts poor prognosis in renal carcinoma. *Mol Cancer Res* 15, 884-895.

Murray, M.Y., Birkland, T.P., Howe, J.D., Rowan, A.D., Fidock, M., Parks, W.C., and Gavrilovic, J. (2013). Macrophage migration and invasion is regulated by MMP10 expression. *PLoS One* 8, e63555.

Nishikawa, R., Goto, Y., Kojima, S., Enokida, H., Chiyomaru, T., Kinoshita, T., Sakamoto, S., Fuse, M., Nakagawa, M., and Naya, Y. (2014). Tumor-suppressive microRNA-29s inhibit cancer cell migration and invasion via targeting LAMC1 in prostate cancer. *Int J Oncol* 45, 401-410.

Park, E.H., Kim, S., Jo, J.Y., Kim, S.J., Hwang, Y., Kim, J.-M., Song, S.Y., Lee, D.-K., and Koh, S.S. (2012). Collagen triple helix repeat containing-1 promotes pancreatic cancer progression by regulating migration and adhesion of tumor cells. *Carcinogenesis* 34, 694-702.

Pasqualini, L., Bu, H., Puhr, M., Narisu, N., Rainer, J., Schlick, B., Schäfer, G., Angelova, M., Trajanoski, Z., and Börno, S.T. (2015). miR-22 and miR-29a are members of the androgen receptor cistrome modulating LAMC1 and Mcl-1 in prostate cancer. *Mol Endocrinol* 29, 1037-1054.

Peng, L., Yanjiao, M., Ai-Guo, W., Pengtao, G., Jianhua, L., Ju, Y., Hongsheng, O., and Xichen, Z. (2011). A fine balance between CCNL1 and TIMP1 contributes to the development of breast cancer cells. *Biochem Biophys Res Comm* 409, 344-349.

Rider, L., Oladimeji, P., and Diakonova, M. (2013). PAK1 regulates breast cancer cell invasion through secretion of matrix metalloproteinases in response to prolactin and three-dimensional collagen IV. *Mol Endocrinol* 27, 1048-1064.

Roscilli, G., Cappelletti, M., De Vitis, C., Ciliberto, G., Di Napoli, A., Ruco, L., Mancini, R., and Aurisicchio, L. (2014). Circulating MMP11 and specific antibody immune response in breast and prostate cancer patients. *J Transl Med* 12, 54.

Rosette, C., Roth, R.B., Oeth, P., Braun, A., Kammerer, S., Ekblom, J., and Denissenko, M.F. (2005). Role of ICAM1 in invasion of human breast cancer cells. *Carcinogenesis* 26, 943-950.

Sakamoto, N., Naito, Y., Oue, N., Sentani, K., Uraoka, N., Zarni Oo, H., Yanagihara, K., Aoyagi, K., Sasaki, H., and Yasui, W. (2014). MicroRNA‐148a is downregulated in gastric cancer, targets MMP7, and indicates tumor invasiveness and poor prognosis. *Cancer Sci* 105, 236-243.

Sathyanarayana, U.G., Maruyama, R., Padar, A., Suzuki, M., Bondaruk, J., Sagalowsky, A., Minna, J.D., Frenkel, E.P., Grossman, H.B., and Czerniak, B. (2004). Molecular detection of noninvasive and invasive bladder tumor tissues and exfoliated cells by aberrant promoter methylation of laminin-5 encoding genes. *Cancer Research* 64, 1425-1430.

Shi, X., Chen, Z., Hu, X., Luo, M., Sun, Z., Li, J., Shi, S., Feng, X., Zhou, C., and Li, Z. (2016). AJUBA promotes the migration and invasion of esophageal squamous cell carcinoma cells through upregulation of MMP10 and MMP13 expression. Oncotarget 7, 36407-18.

Shields, M.A., Dangi-Garimella, S., Krantz, S.B., Bentrem, D.J., and Munshi, H.G. (2011). Pancreatic cancer cells respond to type I collagen by inducing snail expression to promote membrane type 1 matrix metalloproteinase-dependent collagen invasion. *J Biol Chem* 286, 10495-10504.

Shields, M.A., Dangi-Garimella, S., Redig, A.J., and Munshi, H.G. (2012). Biochemical role of the collagen-rich tumour microenvironment in pancreatic cancer progression. *Biochem J* 441, 541-552.

Shoshan, E., Braeuer, R.R., Kamiya, T., Mobley, A.K., Huang, L., Vasquez, M.E., Velazquez-Torres, G., Chakravarti, N., Ivan, C., and Prieto, V. (2016). NFAT1 directly regulates IL8 and MMP3 to promote melanoma tumor growth and metastasis. *Cancer Research* 76, 3145-3155.

Sizemore, S.T., and Keri, R.A. (2012). The forkhead box transcription factor FOXC1 promotes breast cancer invasion by inducing matrix metalloprotease 7 (MMP7) expression. *J Biol Chem* 287, 24631-24640.

Sounni, N.E., Rozanov, D.V., Remacle, A.G., Golubkov, V.S., Noel, A., and Strongin, A.Y. (2010). Timp‐2 binding with cellular MT1‐MMP stimulates invasion‐promoting MEK/ERK signaling in cancer cells. *Int J Cancer* 126, 1067-1078.

Sroka, I.C., Anderson, T.A., Mcdaniel, K.M., Nagle, R.B., Gretzer, M.B., and Cress, A.E. (2010). The laminin binding integrin α6β1 in prostate cancer perineural invasion. *J Cell Physiol* 224, 283-288.

Tan, M.-Y., Mu, X.-Y., Liu, B., Wang, Y., Bao, E.-D., Qiu, J.-X., and Fan, Y. (2013). SUMO-specific protease 2 suppresses cell migration and invasion through inhibiting the expression of MMP13 in bladder cancer cells. *Cell Physiol Biochem* 32, 542-548.

Tang, J., Li, B., Hong, S., Liu, C., Min, J., Hu, M., Li, Y., Liu, Y., and Hong, L. (2017). Punicalagin suppresses the proliferation and invasion of cervical cancer cells through inhibition of the β-catenin pathway. *Mol Med Rep* 16, 1439-1444.

Tiainen, S., Oikari, S., Tammi, M., Rilla, K., Hämäläinen, K., Tammi, R., Kosma, V.-M., and Auvinen, P. (2016). High extent of O-GlcNAcylation in breast cancer cells correlates with the levels of HAS enzymes, accumulation of hyaluronan, and poor outcome. *Breast Cancer Res Treat* 160, 237-247.

Toupance, S., Brassart, B., Rabenoelina, F., Ghoneim, C., Vallar, L., Polette, M., Debelle, L., and Birembaut, P. (2012). Elastin-derived peptides increase invasive capacities of lung cancer cells by post-transcriptional regulation of MMP-2 and uPA. *Clin Exp Metastasis* 29, 511-522.

Tsai, S.-T., Wang, P.-J., Liou, N.-J., Lin, P.-S., Chen, C.-H., and Chang, W.-C. (2015). ICAM1 is a potential cancer stem cell marker of esophageal squamous cell carcinoma. *PLoS One* 10, e0142834.

Tyan, S.-W., Hsu, C.-H., Peng, K.-L., Chen, C.-C., Kuo, W.-H., Eva, Y.-H.L., Shew, J.-Y., Chang, K.-J., Juan, L.-J., and Lee, W.-H. (2012). Breast cancer cells induce stromal fibroblasts to secrete ADAMTS1 for cancer invasion through an epigenetic change. *PLoS One* 7, e35128.

Wan, X., Pu, H., Huang, W., Yang, S., Zhang, Y., Kong, Z., Yang, Z., Zhao, P., Li, A., and Li, T. (2016). Androgen-induced miR-135a acts as a tumor suppressor through downregulating RBAK and MMP11, and mediates resistance to androgen deprivation therapy. *Oncotarget* 7, 51284-300.

Wang, C., Gao, C., Zhuang, J.-L., Ding, C., and Wang, Y. (2012). A combined approach identifies three mRNAs that are down-regulated by microRNA-29b and promote invasion ability in the breast cancer cell line MCF-7. *J Cancer Res Clin Oncol* 138, 2127-2136.

Wang, H., Zhu, Y., Zhao, M., Wu, C., Zhang, P., Tang, L., Zhang, H., Chen, X., Yang, Y., and Liu, G. (2013). miRNA-29c suppresses lung cancer cell adhesion to extracellular matrix and metastasis by targeting integrin β1 and matrix metalloproteinase2 (MMP2). *PLoS One* 8, e70192.

Wang, P.-C., Weng, C.-C., Hou, Y.-S., Jian, S.-F., Fang, K.-T., Hou, M.-F., and Cheng, K.-H. (2014). Activation of VCAM-1 and its associated molecule CD44 leads to increased malignant potential of breast cancer cells. *Int J Mol Sci* 15, 3560-3579.

Wang, X., Lu, H., Urvalek, A.M., Li, T., Yu, L., Lamar, J., Dipersio, C.M., Feustel, P.J., and Zhao, J. (2011). KLF8 promotes human breast cancer cell invasion and metastasis by transcriptional activation of MMP9. *Oncogene* 30, 1901-11.

Willis, C.M., and Klüppel, M. (2014). Chondroitin sulfate-E is a negative regulator of a pro-tumorigenic Wnt/beta-catenin-Collagen 1 axis in breast cancer cells. *PLoS One* 9, e103966.

Willumsen, N., Genovese, F., Hogaboam, C., Sand, J., Martinez, F., Han, M., Skjot-Arkil, H., Karsdal, M., Bay-Jensen, A., and Leeming, D. (2013). Serological detection of matrix metalloproteinase generated fragments of elastin and type IV collagen may be biomarkers of lung metastatic breast cancer. *Ann Oncol* 24, iii35-36.

Wong, J.C., Chan, S.K., Schaeffer, D.F., Sagaert, X., Lim, H.J., Kennecke, H., Owen, D.A., Suh, K.W., Kim, Y.-B., and Tai, I.T. (2011). Absence of MMP2 expression correlates with poor clinical outcomes in rectal cancer, and is distinct from MMP1-related outcomes in colon cancer. *Clin Cancer Res* 17, 4167-4176.

Wu, S., Ma, C., Shan, S., Zhou, L., and Li, W. (2017). High expression of matrix metalloproteinases 16 is associated with the aggressive malignant behavior and poor survival outcome in colorectal carcinoma. *Sci Rep* 7, 46531.

Xiao, X.-Y., Wang, X.-D., and Zang, D.-Y. (2012). MMP1-1607 1G/2G polymorphism and lung cancer risk: a meta-analysis. *Tumor Biol* 33, 2385-2392.

Yeung, T.-L., Ghosh, S., Wong, K.-K., Birrer, M.J., and Mok, S.C. (2011). Up-regulation of stromal versican in advanced stage serous ovarian cancer modifies tumor microenvironment and promotes cancer invasion. *Cancer Res* 71, 420 (Supplement).

Yeung, T.-L., Leung, C.S., Wong, K.-K., Samimi, G., Thompson, M.S., Liu, J., Zaid, T.M., Ghosh, S., Birrer, M.J., and Mok, S.C. (2013). TGF-β modulates ovarian cancer invasion by upregulating CAF-derived versican in the tumor microenvironment. *Cancer Res* 73, 5016-5028.

Yoneda, M., Hirokawa, Y.S., Ohashi, A., Uchida, K., Kami, D., Watanabe, M., Yokoi, T., Shiraishi, T., and Wakusawa, S. (2010). RhoB enhances migration and MMP1 expression of prostate cancer DU145. *Exp Mol Pathol* 88, 90-95.

Zhang, H., Hao, C., Wang, Y., Ji, S., Zhang, X., Zhang, W., Zhao, Q., Sun, J., and Hao, J. (2016). Sohlh2 inhibits human ovarian cancer cell invasion and metastasis by transcriptional inactivation of MMP9. *Mol Carcinog* 55, 1127-1137.

Zhang, J.-J., Zhu, Y., Xie, K.-L., Peng, Y.-P., Tao, J.-Q., Tang, J., Li, Z., Xu, Z.-K., Dai, C.-C., and Qian, Z.-Y. (2014). Yin Yang-1 suppresses invasion and metastasis of pancreatic ductal adenocarcinoma by downregulating MMP10 in a MUC4/ErbB2/p38/MEF2C-dependent mechanism. *Mol Cacner* 13, 130.

Zhang, K., Corsa, C.A., Ponik, S.M., Prior, J.L., Piwnica-Worms, D., Eliceiri, K.W., Keely, P.J., and Longmore, G.D. (2013). The collagen receptor discoidin domain receptor 2 stabilizes SNAIL1 to facilitate breast cancer metastasis. *Nat Cell Biol* 15, 677-87.

Zhao, Y., Jia, L., Mao, X., Xu, H., Wang, B., and Liu, Y. (2009). siRNA‐targeted COL8A1 inhibits proliferation, reduces invasion and enhances sensitivity to D‐limonence treatment in hepatocarcinoma cells. *IUBMB Life* 61, 74-79.

Zhao, Z.-S., Wang, Y.-Y., Chu, Y.-Q., Ye, Z.-Y., and Tao, H.-Q. (2010). SPARC is associated with gastric cancer progression and poor survival of patients. *Clin Cancer Res* 16, 260-268.

Zhou, Y., Hofstetter, W.L., He, Y., Hu, W., Pataer, A., Wang, L., Wang, J., Zhou, Y., Yu, L., and Fang, B. (2010). KLF4 inhibition of lung cancer cell invasion by suppression of SPARC expression. *Cancer Biol Ther* 9, 507-513.

Zhu, M., Zhang, N., He, S., Lui, Y., Lu, G., and Zhao, L. (2014). MicroRNA‐106a targets TIMP2 to regulate invasion and metastasis of gastric cancer. *FEBS Lett* 588, 600-607.

Zu, C., Liu, T., and Zhang, G. (2016). MicroRNA-506 inhibits malignancy of colorectal carcinoma cells by targeting LAMC1. *Ann Clin Lab Sci* 46, 666-674.
